# The Effects of Digital Health Interventions on Motor Symptoms, Nonmotor Symptoms, and Quality of Life in Patients With Parkinson Disease: Systematic Review and Meta-Analysis of Randomized Controlled Trials

**DOI:** 10.2196/79935

**Published:** 2026-03-12

**Authors:** Ruwen Liu, Sirui Zhang, Yi Xiao, Yangfan Cheng, Huifang Shang

**Affiliations:** 1 Department of Neurology, Laboratory of Neurodegenerative Disorders, National Clinical Research Center for Geriatric West China Hospital of Sichuan University Chengdu China; 2 Rare Disease Center West China Hospital of Sichuan University Chengdu China

**Keywords:** digital, eHealth, meta-analysis, motor symptoms, non-motor symptoms, Parkinson disease, Parkinson's disease, quality of life

## Abstract

**Background:**

Parkinson disease (PD) is a progressive neurodegenerative disorder with increasing global prevalence, necessitating innovative management. Digital health interventions (DHIs) offer potential advantages for PD care; yet, a comprehensive systematic review and synthesis across all DHI types and core outcomes is still lacking.

**Objective:**

This review aimed to assess the effectiveness of DHIs for improving motor symptoms, nonmotor symptoms, and quality of life in patients with PD and to summarize the reach, uptake, and feasibility.

**Methods:**

We searched PubMed, Ovid Embase, Web of Science, CINAHL, Cochrane Central Register of Controlled Trials, and APA PsycINFO up to November 2025. Pooled standardized mean differences (SMDs) were calculated using random-effects models. We calculated 95% prediction intervals (PIs) to estimate the true effects. The revised Cochrane Risk of Bias 2 tool was used to assess risk of bias. Heterogeneity was assessed using *I*^2^, τ^2^, and 95% PI. Subgroup analyses, meta-regression, and sensitivity analyses were conducted to address heterogeneity and potential bias. The quality of evidence was assessed using GRADE (Grading of Recommendations Assessment, Development, and Evaluation).

**Results:**

The review included 112 randomized controlled trials involving 5594 participants. Significant postintervention improvements were identified in motor symptoms (SMD=–0.39, 95% CI –0.60 to –0.18, 95% PI –1.75 to 0.99; *I*^2^=80.3%) and overall nonmotor symptoms (SMD=–0.26, 95% CI –0.49 to –0.03, 95% PI –0.56 to 0.03; *I*^2^=13.8%), including cognitive function (SMD=0.47, 95% CI 0.22 to 0.72, 95% PI –0.41 to 1.35; *I*^2^=63.5%) and psychiatric symptoms (SMD=–0.42, 95% CI –0.74 to –0.09, 95% PI –1.82 to –0.99; *I*^2^=85.4%); however, there was no significant enhancement in quality of life (SMD=–0.19, 95% CI –0.47 to 0.09, 95% PI –1.50 to 1.12; *I*^2^=81.2%). The certainty of evidence was very low for quality of life, motor, and psychiatric symptoms and low for cognitive function and overall nonmotor symptoms. Improvements in motor symptoms and cognitive function remained stable at follow-up. Meta-regression analysis indicated that age, percentage of female participants, and supervision mode were possible sources of heterogeneity. Overall, 94 studies reported reach (median 37.5%), 38 reported fidelity (95.7%), and 105 reported dropout rates (9.1%).

**Conclusions:**

In contrast to previous reviews focused on single technologies or outcomes, this review provided the first comprehensive synthesis across all DHI types on multiple outcomes and indicated their potential as nonpharmacological interventions for PD management. However, current evidence is of low to very low certainty, and wide 95% PIs, together with high risk of bias and substantial heterogeneity, indicate considerable uncertainty regarding the true effect in future implementations. Therefore, findings should be interpreted with caution. These findings provide integrated evidence to guide the design and prioritization of future research. The results have important real-world implications, supporting cautious implementation while underscoring the need for more robust trials, particularly in resource-limited settings.

**Trial Registration:**

PROSPERO CRD42023492123; https://www.crd.york.ac.uk/PROSPERO/view/CRD42023492123

## Introduction

Parkinson disease (PD) is the most common serious movement disorder, characterized clinically by bradykinesia, rest tremor, rigidity, and postural and gait abnormalities [1,2]. As the global population ages, the global prevalence of PD is projected to exceed 12 million by 2040 [3,4], representing a significant public health challenge. In addition to motor symptoms, nearly all patients experience complex nonmotor manifestations, including hyposmia, autonomic dysfunction, psychiatric symptoms, cognitive decline, and sleep disorders [1,5,6], which also impair the quality of life of patients with PD [7]. PD management mainly relies on dopamine replacement therapy and incorporates a range of medical and surgical treatments for complex symptoms [5].

However, PD management still faces many obstacles that must be overcome. It remains largely limited to symptomatic treatment and is challenged by long-term motor complications as well as many nonmotor symptoms, which increase the complexity of medication regimens. Crucially, there are still no clinically established disease-modifying therapies to slow or halt neurodegeneration [2,8]. Consequently, long-term, individualized pharmacological optimization and multidisciplinary care are essential for people with PD; yet, delivering sustained, high-quality care in routine outpatient and community settings remains challenging. This challenge may underscore the potential value of digital health approaches.

In addition, nonpharmacological interventions such as physical and cognitive training have shown promise for improving both motor and nonmotor symptoms of PD [5,9], but access to these interventions can be challenging for patients. Patients often need to visit medical institutions in person to receive rehabilitation training, which requires sufficient time and financial resources. Furthermore, the disease may make it difficult or cause reluctance for patients to go out. Moreover, in some economically underdeveloped regions, few medical institutions offer such rehabilitation training. In addition, patient adherence also poses a significant challenge. The combination of high prevalence, multifaceted symptomatology, and absence of disease-modifying treatments makes PD management particularly challenging; therefore, strategies that improve symptoms and quality of life are essential to reduce the burden on patients and health care systems.

Digital technology is now revolutionizing PD management, creating new possibilities across the entire spectrum of patient care, from disease identification and diagnosis [10,11] to treatment and prognosis [12,13]. It offers opportunities to enhance and extend nonpharmacological interventions [14,15]. Emerging evidence highlights the potential of digital health interventions (DHIs) in the long-term management of PD [16]. DHIs, defined as the “discrete functionality of digital technology that is applied to achieve health objectives” [17-19], may be especially valuable for long-term PD management.

A growing number of randomized controlled trials (RCTs) have demonstrated that various types of DHIs, including computer-based cognitive training [20] and telerehabilitation [21], may benefit patients with PD. These digital interventions use diverse digital tools, spanning from wearable sensors [22] to smartphone apps [23], to improve both motor and nonmotor outcomes. DHIs offer several potential advantages (1) they may reduce the frequency of in-person visits and thus relieve pressure on medical services and regional resource imbalances in medical resources through information and communication technology [24], (2) they may augment and enhance conventional rehabilitation and pharmacotherapy to improve engagement and outcomes [25], and (3) they may address patients’ individualized demands in real time by leveraging adaptive systems that analyze real-time health data [26].

Current DHIs for PD management include robot-assisted physical therapy [27], exergaming [28], videoconferencing [29], smartphone apps [30], wearable sensors [31], and others. Although these studies demonstrate the considerable potential of DHIs in PD care, no study has summarized current evidence systematically and comprehensively in this field, as previous reviews primarily focused on single digital intervention types (eg, virtual reality) [32] or specific outcomes (eg, motor symptoms) [33]. A comprehensive synthesis across DHI modalities and health-related outcomes in PD is still lacking.

To fill this gap, we performed a large-scale, updated systematic review and meta-analysis to evaluate the effectiveness of diverse DHIs across multiple health outcomes in patients with PD. We additionally summarized the reported reach, fidelity, and feasibility of these interventions. Our findings aim to inform clinicians in selecting management strategies for PD and to guide priorities for future research.

## Methods

### Protocol and Registration

This systematic review and meta-analysis were preregistered on PROSPERO (CRD42023492123) and were conducted according to the PRISMA (Preferred Reporting Items for Systematic Reviews and Meta-Analyses) guidelines for transparent and comprehensive reporting of methodology and results [34]. The PRISMA 2020 checklist and the PRISMA-S (Preferred Reporting Items for Systematic Reviews and Meta-Analyses Literature Search Extension) checklist are provided in Multimedia Appendices 1 and 2, respectively. Any deviations from the preregistered protocol are outlined and explained in Multimedia Appendix 3.

### Search Strategy and Study Selection

Searches were first performed in PubMed (via NCBI), Ovid Embase (via Ovid), Web of Science (via Clarivate), CINAHL (via EBSCOhost), Cochrane Central Register of Controlled Trials (via Wiley), and APA PsycINFO (via EBSCOhost) from inception to November 21, 2023. Updated database searches were performed in the above databases via the same platforms from November 21, 2023, to January 5, 2025. Final database searches were conducted on November 22, 2025, for articles published in 2025, and email alerts were maintained to capture newly published studies until final data analysis. No study registries, websites, or other non–peer-reviewed online resources were systematically searched to ensure the reliability of the included data. The reference lists of included articles and other relevant systematic reviews were examined. Authors of potentially eligible studies were contacted via email to inquire about additional data or unpublished results.

Three groups of search terms were used: terms related to PD, terms related to DHIs (eg, digital, technology, telemedicine, internet, robotics, virtual reality, and computers), and terms related to RCTs. Complete search strategies and search limits are detailed in Multimedia Appendix 4. Search results were imported into EndNote 20 (Clarivate), and duplicates were removed using the software’s automatic deduplication feature, followed by manual review. The titles and abstracts of the involved studies were first screened, and then the full texts of potentially eligible studies were reviewed according to the inclusion criteria.

The inclusion criteria were developed based on the PICOS (Population, Intervention, Comparison, Outcomes, and Study Type) framework. First, for population, we included studies involving patients with a confirmed diagnosis of PD. Second, for intervention, interventions provided through the computer, smartphone, tablet, virtual reality, wearable sensor, robot, or any other digital technology for physical rehabilitation, care aid, or cognitive training were defined as DHIs in this review. DHIs were eligible regardless of setting (eg, used in medical institution vs at home), mode of interaction and supervision, intervention content, or intervention duration. Although digital technology also contributes to the application of deep brain stimulation, transcranial direct current stimulation, and repetitive transcranial magnetic stimulation, these interventions with specific therapeutic aims were not in the scope of this review. DHIs solely used for monitoring parameters related to the disease symptoms were not included. Third, for comparison, comparators were categorized into passive control and active control. Passive control refers to waitlist control or no intervention. Active control included traditional, nontechnological caregiving methods, such as conventional physical rehabilitation, standard care aid, or drug treatments. Studies comparing the effectiveness of different modes of DHIs were excluded. Fourth, for outcome, we considered any outcomes related to motor symptoms, nonmotor symptoms, and quality of life in patients with PD as eligible to provide a comprehensive summary. Studies that only reported the reach, fidelity, or feasibility of the DHIs in PD were also included. Fifth, for study type, only RCT studies were included in this review. Studies with fewer than 5 participants in each group were excluded from the quantitative meta-analysis to ensure the robustness of the results.

The entire process of study selection was performed by 2 researchers independently, and disagreements were resolved with the involvement of a third researcher. Interrater reliability was assessed using Cohen κ [35], which was 0.831 for full-text review, indicating a high agreement between the 2 researchers.

### Data Extraction

One reviewer extracted data using a comprehensive data extraction form: publication and author details (title, year, first author’s name, and country), study details (study design, study setting, sample size, dropout, and follow-up duration), participants’ characteristics (age, sex, and disease severity), intervention information (type of intervention, purpose of the intervention, intervention duration, and supervision mode), comparison information (type of comparator), and outcomes (scales used for assessing outcomes, reach, fidelity, and feasibility of interventions). The extracted data were checked by a second reviewer. Discrepancies in data extraction between the 2 researchers were resolved through discussion with a third reviewer.

### Types of DHIs

DHIs were classified into three categories: (1) digital databases, (2) online classes, and (3) technology-based rehabilitation devices, based on the interaction modalities influenced by the technical implementation and the level of supervision inherent to the intervention, following the classification framework recommended by a previous review [36].

Digital databases are platforms that store health- or disease-related information in the form of videos or textual materials and offer self-paced access to predesigned resources, characterized by asynchronous interaction and a minimal level of supervision. Online classes involve health management and telerehabilitation sessions delivered in real time through telephone or video conferencing, offering synchronous interaction and direct supervision by professionals remotely and allowing for immediate feedback and guidance. Technology-based rehabilitation devices encompass a broad range of technologies, including exergaming, virtual reality, robotics, and mobile apps, which support physical rehabilitation or cognitive training. These devices involve close interactions with users, who respond to guidance and instructions provided by the DHIs, which in turn provide cues and feedback based on the users’ actions. The level of supervision in these interventions varies depending on the technical features of the devices (eg, sensor integration) and the study design (eg, use under professional supervision).

Additionally, DHIs were categorized into physical rehabilitation, cognitive training, and care aid according to the intervention purpose.

### Outcome Measures

The primary outcomes of the review were changes in symptom severity, including motor symptoms, cognitive function, psychiatric symptoms, overall nonmotor symptoms, and quality of life from baseline to postintervention and last follow-up. The scales used for assessing the severity of each symptom and quality of life depended on the original study. When one study utilized multiple assessment scales for one type of syndrome (eg, Parkinson’s Disease Questionnaire-8 and Parkinson’s Disease Questionnaire-39 for quality of life), the most frequently used one among all included studies was selected for analysis to reduce potential heterogeneity between studies. The assessment scales for each outcome used in each study are summarized in Multimedia Appendix 5.

The secondary outcomes included percentage reach, fidelity, dropout rate, and feasibility. According to the Medical Research Council process evaluation framework and Proctor implementation outcomes, we used the more common term reach to replace penetration, which is defined as the integration of a practice within a service setting and its subsystems [37,38]; therefore, we calculated the proportion of people who come into contact with the intervention. Fidelity was defined as the degree to which an intervention was implemented as prescribed in the original protocol or as intended by the program developers [38], and we extracted the fulfillment of the intervention. Feasibility was defined as the extent to which a new treatment or innovation can be successfully used or carried out within a given agency or setting [38] and can be measured by retention, acceptability, adherence, satisfaction, preference, safety, and cost-effectiveness [39]. Feasibility was summarized according to the definition of each study, and the aforementioned items were extracted even if the study did not discuss feasibility.

### Quality Assessment

The revised Cochrane Risk of Bias 2 tool [40] was used to assess study quality across the following domains: randomization process, deviations from intended interventions, missing outcome data, measurement of the outcome, and selection of the reported result. Studies were categorized as low risk, some concerns, or high risk. We used the GRADE (Grading of Recommendations Assessment, Development, and Evaluation) criteria [41] to assess the certainty of the overall evidence, which incorporates 5 key considerations: study limitations, inconsistency of effects, indirectness, imprecision, and publication bias. Any deviation across these 5 domains resulted in downgrading of the quality of evidence. The overall certainty of evidence was classified as high, moderate, low, or very low. All quality assessments were independently conducted by 2 reviewers, and any discrepancies were resolved through discussion.

### Meta-Analysis Methods

Pairwise meta-analyses were performed to evaluate the effect of DHIs on the primary outcomes when more than 5 effect estimates were available in the analysis. The means and SDs of within-group differences from baseline to postintervention and follow-up assessments were used. If means and SDs were not reported in a study, the corresponding author was contacted via email to obtain the data. Otherwise, means and SDs were calculated based on available data using recommended formulas [42,43].

Standardized mean differences (SMDs; Hedges g) at postintervention and the last follow-up assessment were used as the effect measure, allowing comparison of data from different assessment scales. In studies with more than 2 intervention groups, the intervention group demonstrating the most favorable effect was selected for the main analysis as it represented the most well-designed DHIs. In studies featuring multiple control groups, the most active control group was chosen to adopt a more conservative analytical approach.

Considering that different assessment scales may reflect symptom severity in opposite directions, data from the original studies for the same outcome were standardized based on the most commonly used scale. For instance, while higher scores on the Unified Parkinson’s Disease Rating Scale Part 3 indicate more severe motor symptoms, higher scores on the 6-Minute Walk Test suggest milder symptoms. Therefore, the results of the 6-Minute Walk Test were inverted (multiplied by –1) to align with the Unified Parkinson’s Disease Rating Scale Part 3 for the analysis.

Due to the presence of potential heterogeneity, the random-effects model and the DerSimonian-Laird estimator were applied to calculate pooled estimates across studies. The Hartung-Knapp-Sidik-Jonkman method was applied to increase the robustness of the results [44]. Forest plots were generated to visually display the effect size and 95% CIs for individual studies and the overall pooled estimate. To present the expected range of true effects in similar studies, the 95% prediction intervals (PIs) were also calculated and displayed in the forest plot [45]. When the number of studies was fewer than 10, the method proposed by Nagashima et al [46] was applied to adjust the 95% PI. The 95% CI reflects the precision of the estimated overall average effect, whereas the 95% PI estimates the distribution of effects within which the true effect size is expected to fall in future similar studies. These 2 intervals complement each other to provide comprehensive information for result interpretation.

Subgroup analyses were conducted based on the intervention type (technology-based rehabilitation devices, online classes, and digital databases), considering the different inherent characteristics of various DHIs. Pairwise comparisons between subgroups were performed using random-effects meta-analysis with Hartung-Knapp adjustment and were limited to subgroups with 3 or more studies. Funnel plots and Egger regression intercept were used to assess small-study effects, which may indicate the potential publication bias. A sensitivity analysis was conducted to evaluate whether the overall results were statistically and significantly affected by 1 individual study using the leave-one-out analysis method. In addition, another sensitivity analysis restricting the synthesis to studies with a low risk of bias was performed for outcomes for which at least 10 such studies were available. All meta-analyses were conducted using R (version 4.2.3; R Foundation for Statistical Computing).

### Heterogeneity and Meta-Regression

The presence of heterogeneity among studies was assessed using Cochran Q test and the *I*^2^ statistic, whereas 95% PI, τ, and τ^2^ were calculated to evaluate the magnitude of heterogeneity [45]. By estimating the expected range of true effects for future studies, the 95% PI directly evaluates the variability of the intervention effect across diverse settings [47]. τ represents the estimated SD of the between-study effects, and τ² denotes the variance, which quantifies the spread of the true study effects around the mean [48].

Because substantial heterogeneity was anticipated to result from various types of DHIs in the analysis, univariate meta-regression analyses were performed using the mixed-effects model with the DerSimonian-Laird estimator for between-study variance and the Hartung-Knapp-Sidik-Jonkman method for SE adjustment to identify potential moderators that could account for heterogeneity. These potential moderators included individual-level variables (mean age, sex, and disease severity) and study-level variables (publication years, country income level, intervention type, intervention purpose, intervention setting, type of control, intervention duration, and supervision mode). In addition, bubble plots were provided as visual representation of the meta-regressions.

## Results

### Study Search and Selection

The search initially yielded 30,918 related study records from 6 databases in November 2023, among which 12,909 duplicate records were first removed during screening. The titles and abstracts of the remaining 18,009 records were screened, and the full texts of 284 studies were further assessed. A total of 90 studies from 91 articles evaluating the effect of DHI in patients with PD were included.

A supplementary search was conducted on January 5, 2025, and 4179 new publications were identified using the same keywords and databases as the initial search. After removing duplicates, a total of 3019 unique records were screened based on title and abstract, and the full texts of 66 potentially eligible studies were then reviewed. A total of 11 studies meeting the inclusion criteria were included.

The final search was performed on November 22, 2025, to ensure the inclusion of the most recent evidence and identified 3980 new publications. Following deduplication, a total of 2481 unique records were screened by title and abstract. Subsequently, a total of 44 studies underwent full-text review, resulting in the inclusion of 11 studies that met the eligibility criteria. During the full-text screening process, no studies were identified that appeared to meet the inclusion criteria but were ultimately excluded; all exclusions were straightforward decisions based on eligibility criteria.

**Figure 1:**
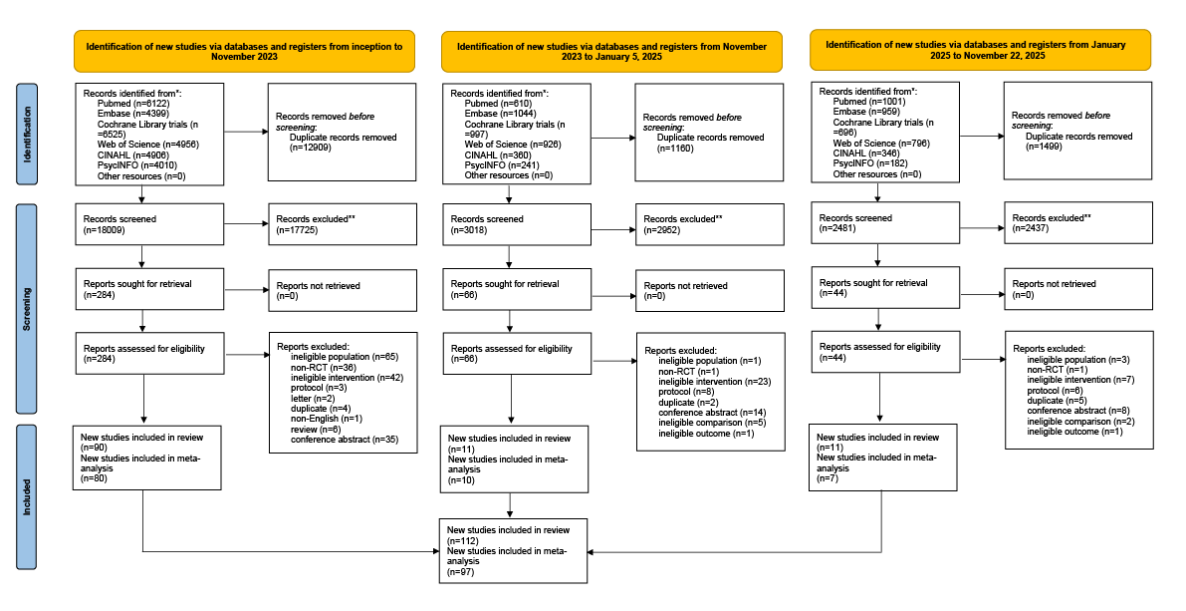
The PRISMA (Preferred Reporting Items for Systematic Reviews and Meta-Analyses) flow diagram for study selection.

Ultimately, a total of 112 unique studies from 115 articles [27,30,31,49-158] were eligible for the systematic review, and 97 unique studies from 98 articles were included in the quantitative meta-analysis. Of these 112 studies, 73 evaluated motor symptoms, 8 evaluated overall nonmotor symptoms, 26 evaluated cognitive function, 30 evaluated psychiatric symptoms, and 41 evaluated quality of life. The PRISMA flow diagram of the study search and selection is presented in Figure 1.

### Study Characteristics

A total of 112 included studies comprising 5594 participants were identified, among which 84 studies (4658 participants) were performed in high-income countries, whereas only 28 studies (936 participants) were performed in low- and middle-income countries (LMICs). According to the predetermined classification criteria, the DHIs used in 91 studies were categorized as technology-based rehabilitation devices, 17 as online classes, and 8 as digital databases. The intervention purposes of 74 studies were physical rehabilitation, 28 were cognitive training, and 17 were care aid.

Active comparators (eg, traditional and nontechnological caregiving methods) were used in 91 trials, whereas others used passive comparators (eg, waitlist control or no intervention). The study setting of 51 studies was at home, and that of 55 studies was in medical institutions. Participants in the study by Halpern et al [88] first received intervention in the clinic for 9 sessions and then at home.

Regarding delivery mode, most interventions were delivered through computers and virtual reality devices; other modes included wearable sensors, robots, tablets, smartphones, and others. Most interventions lasted for at least 4 weeks, whereas 4 did not [51,54,92,115]. Among the 39 (34.8%) studies that reported follow-up assessments, the follow-up duration ranged from 4 to 24 weeks in 38 studies, except for the study by Yang et al [143], which implemented a shorter 2-week follow-up period.

A total of 97 of 112 studies were included in the meta-analysis, with the remaining 15 retained in the systematic review because of unavailable outcome data. A comprehensive summary of the characteristics of the included studies is presented in Multimedia Appendix 6.

### Quality of Studies

**Figure 2:**
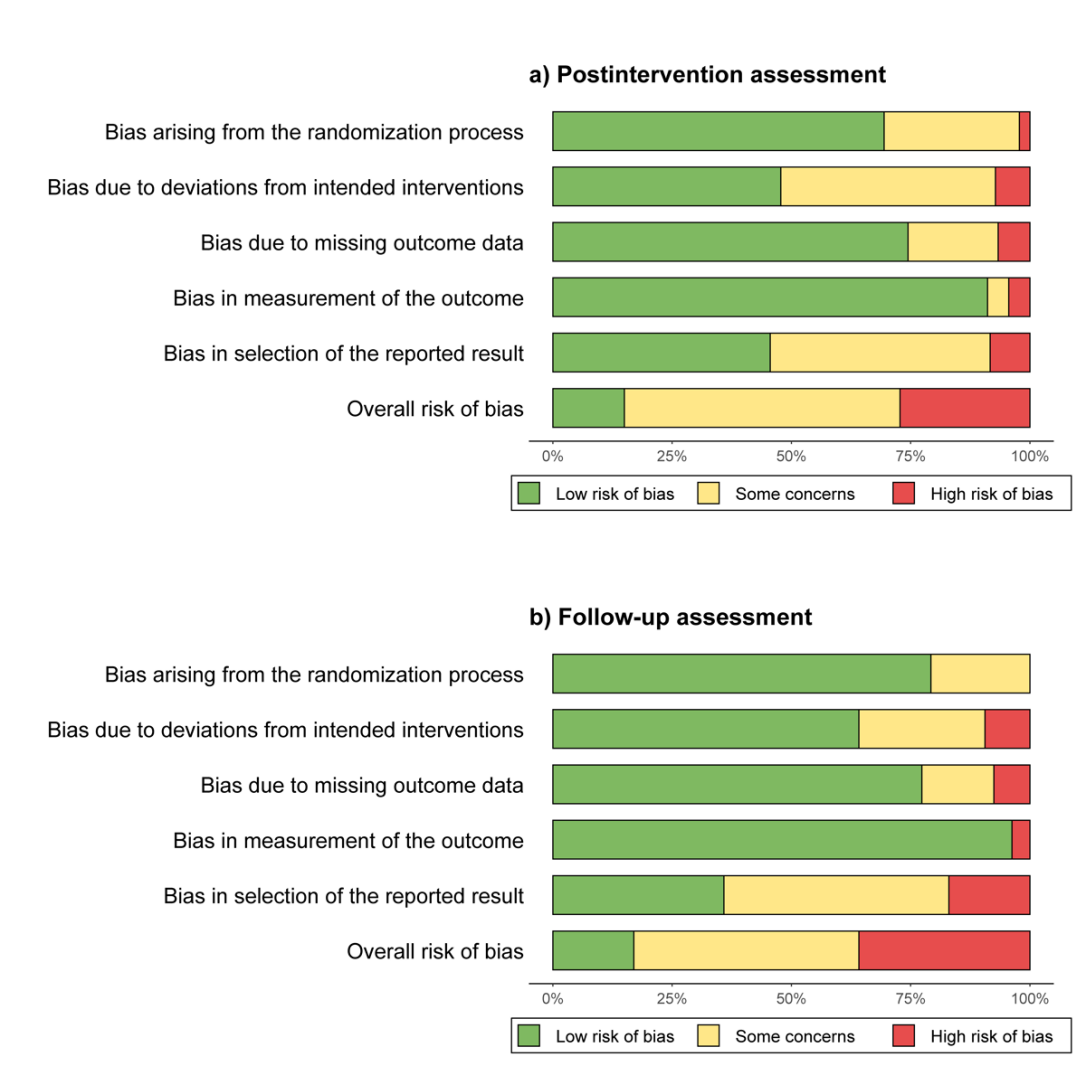
Risk of bias across effect estimates at postintervention and follow-up assessments based on the Cochrane Risk of Bias 2 tool for randomized trials.

The methodological limitations assessed by the Cochrane Risk of Bias 2 tool revealed that ≥20% of the included studies exhibited some concerns or high risk in the following domains (Figure 2): randomization process (postintervention: 53/180, 29% and follow-up: 11/55, 20%), deviations from intended interventions (postintervention: 95/180, 53% and follow-up: 19/55, 34%), missing outcome data (postintervention: 46/180, 26% and follow-up: 13/55, 24%), and selection of reported results (postintervention: 96/180, 53% and follow-up: 33/55, 60%). The main sources of bias were inadequate reporting of randomization methods (eg, lack of explicit allocation concealment) and failure to blind participants or caregivers in trials. Regarding motor symptoms, 12 studies were assessed as having a low risk of bias, 43 studies as having some concerns, and 18 studies as having a high risk of bias. Regarding cognitive function, 6 studies were assessed as having a low risk of bias, 16 studies as having some concerns, and 4 studies as having a high risk of bias. Regarding psychiatric symptoms, 4 studies were assessed as having a low risk of bias, 15 studies as having some concerns, and 10 studies as having a high risk of bias. Regarding overall nonmotor symptoms, 5 studies were assessed as having some concerns and 3 as having a high risk of bias. Regarding quality of life, 5 studies were assessed as having a low risk of bias, 22 studies as having some concerns, and 14 studies as having a high risk of bias (Multimedia Appendix 7).

### Effect of DHIs on PD Across Different Symptoms

#### Motor Symptoms

**Figure 3:**
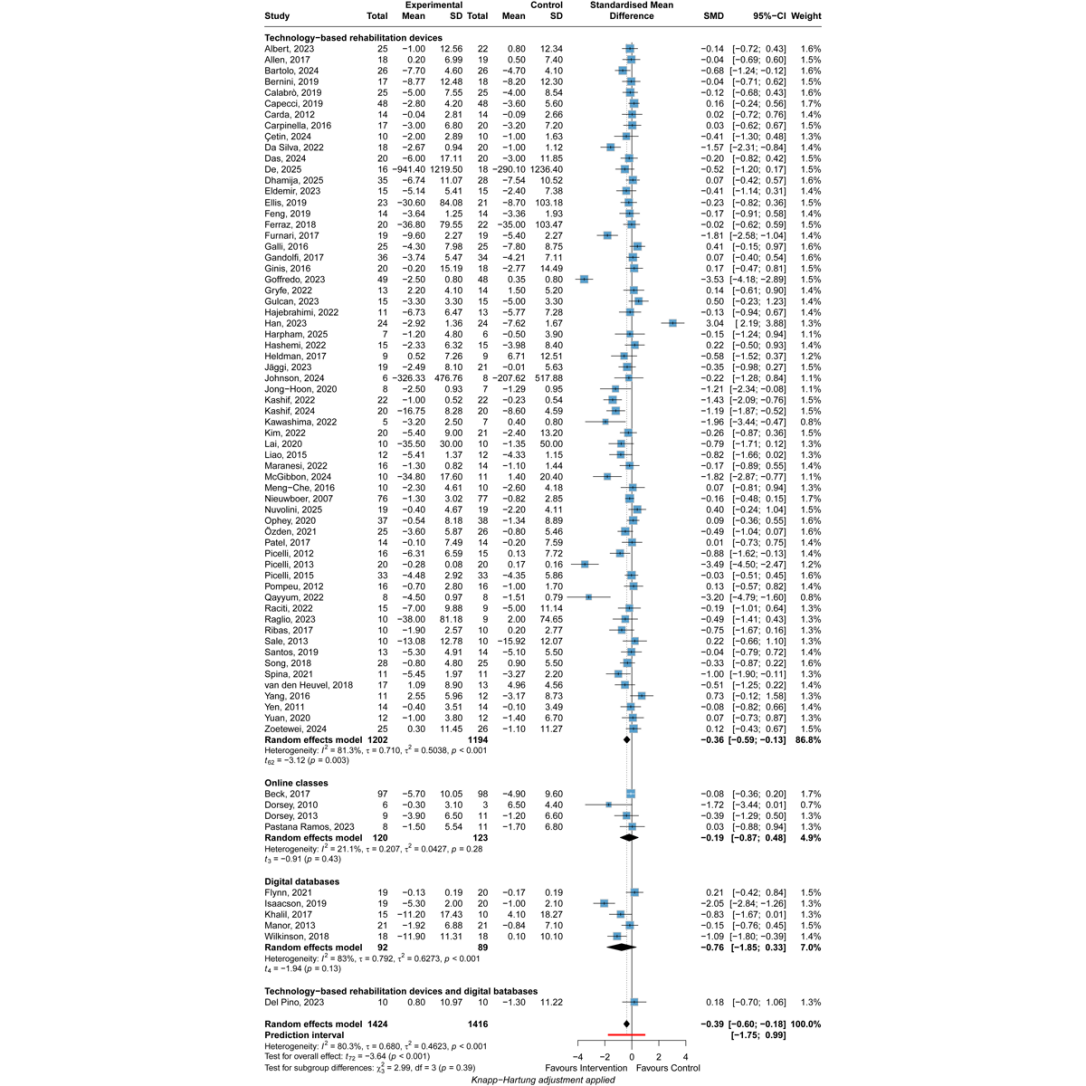
Forest plot of the meta-analysis of digital health interventions’ efficacy on postintervention motor symptoms [27,31,49-53,56-59,61,62,65,68,69,71,72,74-79,82,84-87,89-97,99,100,103,106,110-113,115-117,119,120,122-124,126-132,135,136,141-146,151-153,156]. Standardized mean differences (SMDs) with 95% CIs were calculated using a random-effects model. Negative SMD values favor the experimental intervention. Subgroup analyses were performed by intervention type (technology-based rehabilitation devices, online classes, digital databases). The square represents the individual study estimate. The rhombus shape represents the pooled estimates of the lowest accuracy in all studies. *P* values are shown as p.

**Figure 4:**
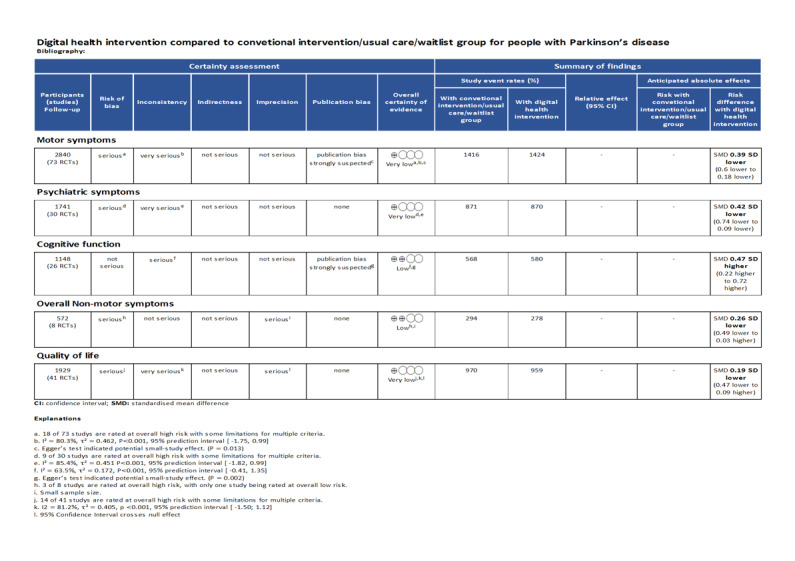
GRADE (Grading of Recommendations Assessment, Development, and Evaluation) ratings at postintervention assessments.

DHIs significantly improve motor symptoms (SMD=–0.39, 95% CI –0.60 to –0.18), while the 95% PI (–1.75 to 0.99) indicates that the true effect is uncertain and could range from a substantial benefit to being inferior to the control condition in future implementations (Figure 3 [27,31,49-53,56-59,61,62,65,68,69,71,72,74-79,82,84-87, 89-97,99,100,103,106,110-113,115-117,119,120, 122-124,126-132,135,136,141-146,151-153,156]). Although the intervention group had fewer symptoms on average (SMD=–0.39), the PI suggests considerable variability in future outcomes. DHIs may improve symptoms (SMD=–1.75) in some populations, while in other populations DHIs may be less effective than control groups (SMD=0.99). Also, DHIs may not affect other populations since the PI contains the 0 value. However, only 1 of 73 included studies suggested that DHIs were inferior to standard training, while both led to improvements in motor symptoms [89]. Subgroup analyses revealed no statistically significant differences in pairwise comparisons among the 3 intervention types (Multimedia Appendix 8). Only technology-based rehabilitation devices significantly improve motor symptoms (SMD=–0.36, 95% CI –0.59 to –0.13, 95% PI –1.80 to 1.07). Neither online classes nor digital databases exhibited favorable effects, with SMD=–0.19 (95% CI –0.87 to 0.48) for online classes, and SMD=–0.76 (95% CI –1.85 to 0.33) for digital databases. However, the subgroup analysis revealed no statistically significant difference in effects across intervention types (*P*=.39). Significant heterogeneity was found (*I*^2^=80.3%, *P*<.001), and the heterogeneity was substantial (τ=0.680 and τ^2^=0.4623). The intervention types did not completely explain the significant heterogeneity, with *I*^2^=81.3% for technology-based rehabilitation devices (*P*<.001) and *I*^2^=83% for digital databases (*P*<.001). In the follow-up analysis, DHIs also exhibited significant improvement in the motor symptoms of patients with PD (Multimedia Appendix 9). The leave-one-out analysis obtained a consistent result. The sensitivity analysis of 12 low-risk-of-bias studies showed a significant but modest benefit of DHIs on motor symptoms (SMD=–0.22, 95% CI –0.40 to –0.04), with negligible heterogeneity (*I*^2^=0.6%; τ=0.025; τ^2^=0.0006; 95% PI –0.43 to –0.01). The 95% PI indicated a high certainty that the intervention will yield at least a small beneficial effect in future similar study settings. GRADE ratings indicated that the certainty of the evidence for the effect of DHIs on motor symptoms of patients with PD was very low owing to the high risk of bias of included studies, high heterogeneity, and potential publication bias (Figure 4).

#### Overall Nonmotor Symptoms

**Figure 5:**
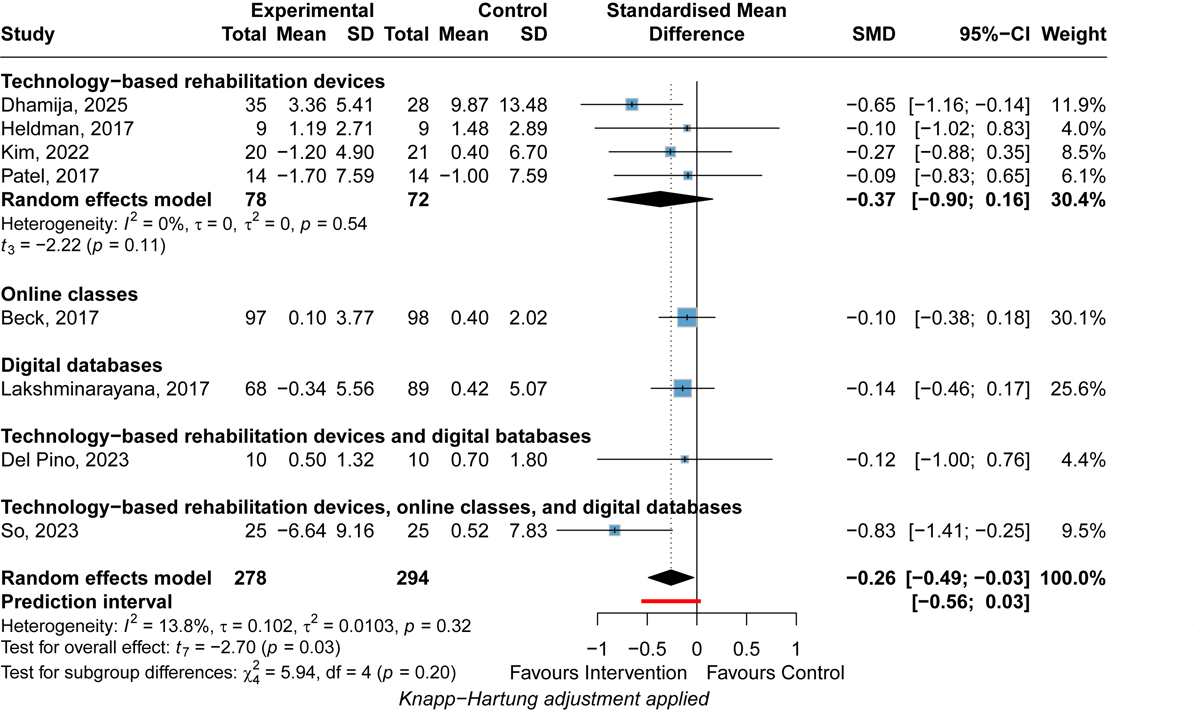
Forest plot of the meta-analysis of digital health interventions’ efficacy on postintervention overall nonmotor symptoms [30,52,65,91,100,120,134,152]. Standardized mean differences (SMDs) with 95% CIs were calculated using a random-effects model. Negative SMD values favor the experimental intervention. Subgroup analyses were performed by intervention type (technology-based rehabilitation devices, online classes, digital databases). The square represents the individual study estimate. The rhombus shape represents the pooled estimates of the lowest accuracy in all studies. *P* values are shown as p.

DHIs significantly improved overall nonmotor symptoms (SMD=–0.26, 95% CI –0.49 to –0.03, 95% PI –0.56 to 0.03; *I*^2^=0; Figure 5 [30,52,65,91,100,120,134,152]). No significant heterogeneity was observed for the analysis (*I*^2^=13.8%; *P*=.32). The leave-one-out analysis did not obtain a consistent result after omitting the studies by Dhamija et al [152], and So et al [134], suggesting the instability of this finding. GRADE ratings indicated that the certainty of the evidence for the effect of DHIs on overall nonmotor symptoms of patients with PD was low owing to the high risk of bias of included studies and serious imprecision (Figure 4).

#### Psychiatric Symptoms

**Figure 6:**
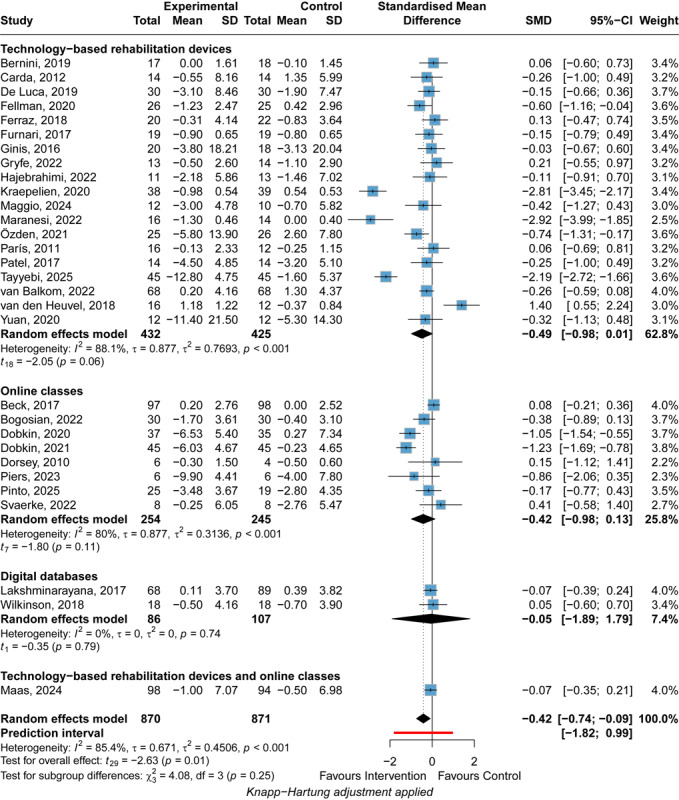
Forest plot of the meta-analysis of digital health interventions’ efficacy on postintervention psychiatric symptoms [30,52,53,55,57,63,66-68,73,75,77,82,85,87,102,107,109,111,117,118,120,125,137,139,141,142,145,157,158]. Standardized mean differences (SMDs) with 95% CIs were calculated using a random-effects model. Negative SMD values favor the experimental intervention. Subgroup analyses were performed by intervention type (technology-based rehabilitation devices, online classes, digital databases). The square represents the individual study estimate. The rhombus shape represents the pooled estimates of the lowest accuracy in all studies. *P* values are shown as p.

DHIs significantly improved psychiatric symptoms (SMD=–0.42, 95% CI –0.74 to –0.09), while the 95% PI (–1.82 to 0.99) indicates that the true effect is uncertain and could range from a substantial benefit to being inferior to the control condition in future implementations (Figure 6 [30,52,53,55,57,63,66-68,73,75,77,82,85,87,102,107, 109,111,117,118,120,125,137,139,141,142,145,157,158]). Although the intervention group had fewer symptoms on average (SMD=–0.42), the PI suggests considerable variability in future outcomes. DHIs may improve symptoms (SMD=–1.82) in some populations, while in other populations DHIs may be less effective than control groups (SMD=0.99). Also, DHIs may not affect other populations since the PI contains the 0 value. However, only 1 of 30 included studies suggested that DHIs were significantly harmful to psychiatric symptoms. Subgroup analyses revealed no statistically significant differences in pairwise comparisons among the intervention types (Multimedia Appendix 8). No types of DHIs exhibited significantly favorable effects in the subgroup analysis, with SMD=–0.49 (95% CI –0.98 to 0.01) for technology-based rehabilitation devices, SMD=–0.05 (95% CI –1.89 to 1.79) for digital databases, and SMD=–0.42 (95% CI –0.98 to 0.13) for online classes. Significant heterogeneity was found (*I*^2^=85.4%; *P*<.001), and the heterogeneity was substantial (τ=0.671; τ^2^=0.4506; 95% PI –1.82 to 0.99). The intervention types could not completely explain the significant heterogeneity, with *I*^2^=88.1% for technology-based rehabilitation devices (*P*<.001) and *I*^2^=80% for online classes (*P*<.001). In the follow-up analysis, DHIs did not exhibit significant improvement in the psychiatric symptoms of patients with PD (Multimedia Appendix 9). The leave-one-out analysis obtained a consistent result. GRADE ratings indicated that the certainty of the evidence for the effect of DHIs on psychiatric symptoms of patients with PD was very low owing to the high risk of bias of included studies and high heterogeneity (Figure 4).

#### Cognitive Function

**Figure 7:**
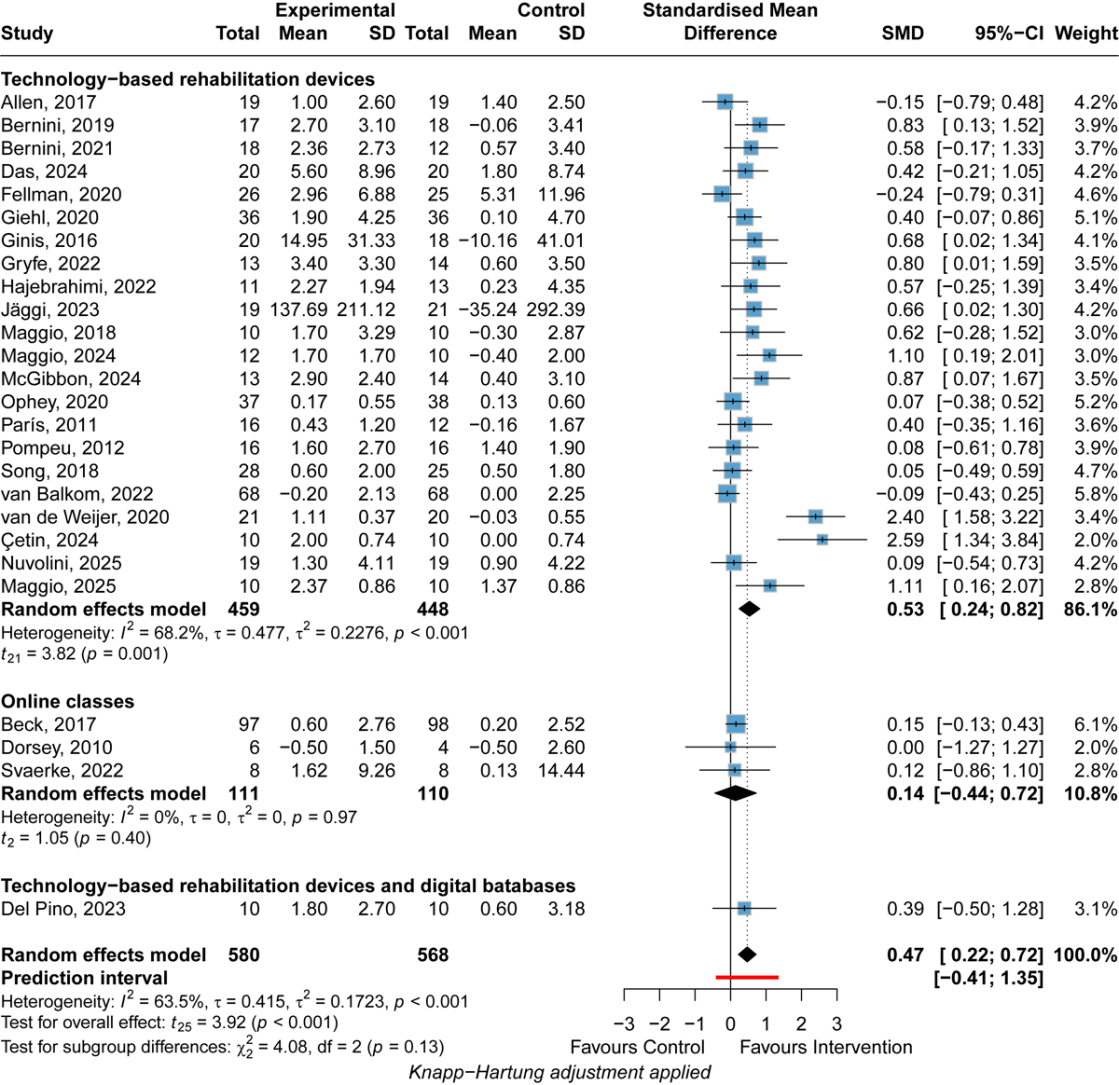
Forest plot of the meta-analysis of digital health interventions’ efficacy on postintervention cognitive function [50,52-54,59,62,65,68,73,80,82,85,87,92,108,109,112,116,118,126,135,137,139,140,155,156]. Standardized mean differences (SMDs) with 95% CIs were calculated using a random-effects model. Negative SMD values favor the experimental intervention. Subgroup analyses were performed by intervention type (technology-based rehabilitation devices, online classes, digital databases). The square represents the individual study estimate. The rhombus shape represents the pooled estimates of the lowest accuracy in all studies. *P* values are shown as p.

DHIs significantly improve cognitive function (SMD=0.47, 95% CI 0.22 to 0.72), while the 95% PI (–0.41 to 1.35) indicates that the true effect is uncertain and could range from a substantial benefit to being inferior to the control condition in future implementations (Figure 7 [50,52-54,59,62,65,68,73,80,82,85,87,92,108, 109,112,116,118,126,135,137,139,140,155,156]). Although the intervention group had fewer symptoms on average (SMD=0.47), the PI suggests considerable variability in future outcomes. DHIs may improve symptoms (SMD=1.35) in some populations, while in other populations DHIs may be less effective than control groups (SMD=–0.41). Also, DHIs may not affect other populations since the PI contains the 0 value. However, no included studies suggested that DHIs were significantly harmful to cognitive function. Technology-based rehabilitation devices demonstrated significantly greater improvement in cognitive function compared with online classes (SMD=0.39, 95% CI 0.01 to 0.77; *P*=.049; Multimedia Appendix 8). Technology-based rehabilitation devices exhibited significant improvement in cognitive functions of patients with PD (SMD=0.53, 95% CI 0.24 to 0.82), but not online classes (SMD=0.14, 95% CI –0.44 to 0.72). Significant heterogeneity was found (*I*^2^=63.5%; *P*<.001), and the heterogeneity was substantial (τ=0.415 and τ^2^=0.1723). The intervention types could not explain the significant heterogeneity, with *I*^2^=68.2% for technology-based rehabilitation devices (*P*<.001). In the follow-up assessment, DHIs also exhibited significant improvement in the cognitive function of patients with PD (Multimedia Appendix 9). The leave-one-out analysis obtained a consistent result. GRADE ratings indicated that the certainty of the evidence for the effect of DHIs on the cognitive function of patients with PD was low owing to high heterogeneity and potential publication bias (Figure 4).

#### Quality of Life

**Figure 8:**
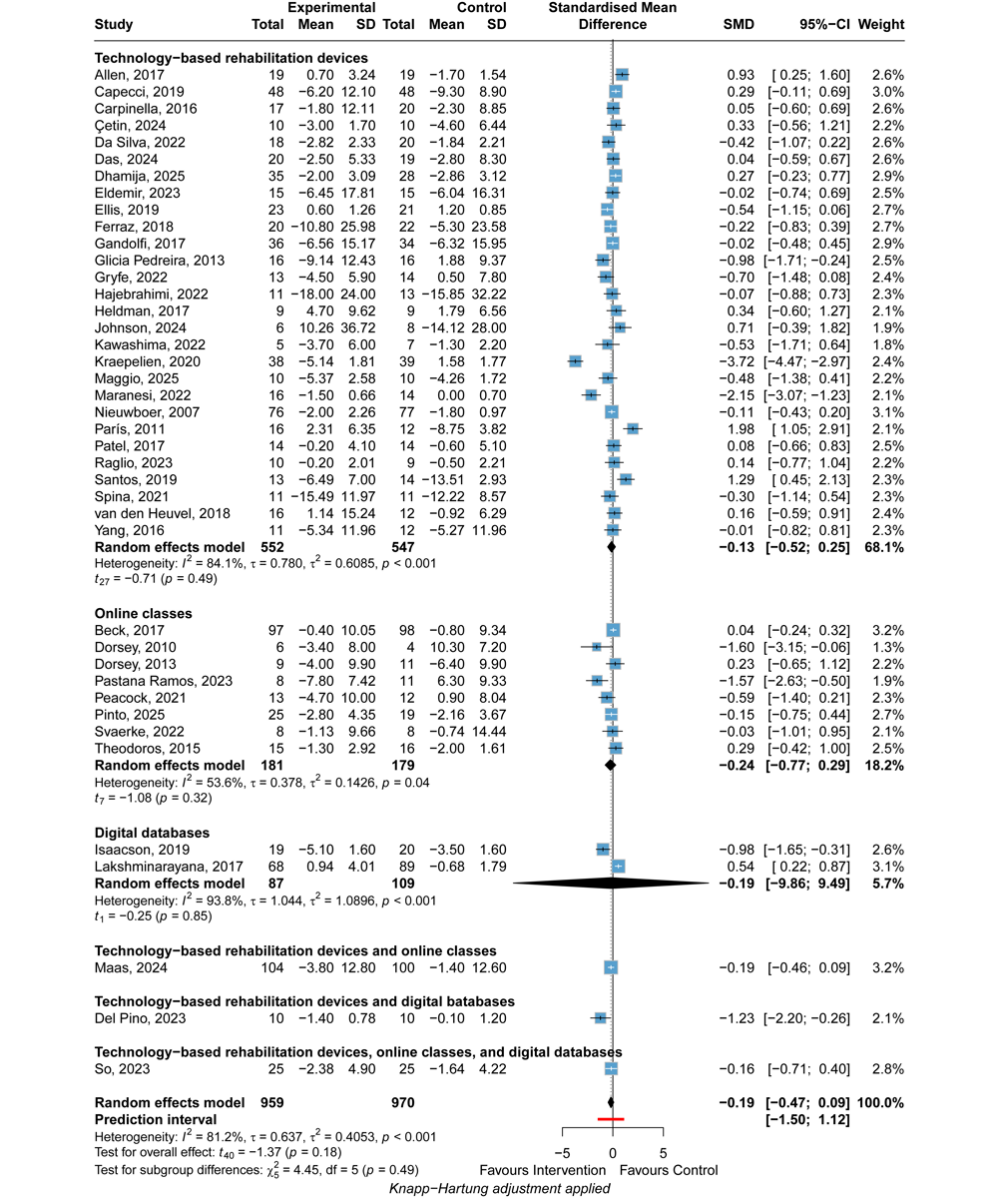
Forest plot of the meta-analysis of digital health interventions’ efficacy on postintervention quality of life [27,30,31,50,52,58,59,61,62,65,68,69,71,72,75,79,83,85,87,91,93,97,102,107,111,115,118-121,129,132,134,136-138,141,143,152,155,157]. Standardized mean differences (SMDs) with 95% CIs were calculated using a random-effects model. Negative SMD values favor the experimental intervention. Subgroup analyses were performed by intervention type (technology-based rehabilitation devices, online classes, digital databases). The square represents the individual study estimate. The rhombus shape represents the pooled estimates of the lowest accuracy in all studies. *P* values are shown as p.

DHIs did not improve quality of life in patients with PD (SMD=–0.19, 95% CI –0.47 to 0.09), and the 95% PI (–1.50 to 1.12) also indicates the uncertain true effect (Figure 8 [27,30,31,50,52,58,59,61,62,65,68,69,71,72,75,79,83,85,87, 91,93,97,102,107,111,115,118-121,129,132,134,136-138,141,143,152,155,157]). Subgroup analyses revealed no statistically significant differences in pairwise comparisons among the intervention types (Multimedia Appendix 8). The analysis of each intervention type obtained a consistent result, with SMD=–0.13 (95% CI –0.52 to 0.25) for technology-based rehabilitation devices, SMD=–0.24 (95% CI –0.77 to 0.29) for online classes, and SMD=–0.19 (95% CI –9.86 to 9.49) for digital databases. Significant heterogeneity was found (*I*^2^=81.2%; *P*<.001), and the heterogeneity was substantial (τ=0.634; τ^2^=0.4053; 95% PI –1.50 to 1.12). The intervention types could not completely explain the heterogeneity, with *I*^2^=84.1% for technology-based rehabilitation devices (*P*<.001) and *I*^2^=53.6% for online classes (*P*=.04). The follow-up analysis indicated a consistent result (Multimedia Appendix 9). The leave-one-out analysis obtained a consistent result. GRADE ratings indicated that the certainty of the evidence for the effect of DHIs on quality of life of patients with PD was very low owing to the high risk of bias of included studies, high heterogeneity, and serious imprecision (Figure 4).

### Publication Bias

Small-study effect was assessed via visual inspection of a funnel plot and Egger test (Multimedia Appendix 10). The Egger test indicated a significant small-study effect in the analysis of the effect of DHIs on motor (intercept=–2.07, 95% CI –3.69 to –0.45; *P*=.02) and cognitive function (intercept=2.54, 95% CI –1.12 to 3.97; *P*=.002) of patients with PD. Further, trim-and-fill analysis (Multimedia Appendix 10) was performed, and we observed that the effects were no longer significant for both motor symptoms (SMD=–0.04, 95% CI –0.29 to 0.21) and cognitive function (SMD=0.16, 95% CI –0.14 to 0.47).

### Meta-Regressions

To explore potential sources of significant heterogeneity, we performed univariate meta-regression analyses examining 3 individual-level and 8 study-level variables (Multimedia Appendix 11), and we also present bubble plots to visualize the relationship involving the continuous variables (Multimedia Appendix 12). We conducted multivariable meta-regressions using the variables mentioned above as predictors of between-study variance when 2 or more variables accounted for *R*^2^. In the analysis of cognitive functions, 2 of these study variables accounted for *R*^2^: mean age of participants (model Q statistic [QM]=0.08; *P*=.78; *R*^2^=0.49%) and intervention setting (QM=1.16; *P*=.29; *R*^2^=8.88%), with a total *R*^2^=1.38% (*P*=.53). There are 4 variables associated with the improvement of psychiatric symptoms with a total *R*^2^=9.83% (*P*=.35): the percentage of female participants (QM=2.73; *P*=.11; *R*^2^=7.42%), publication year (QM=3.39; *P*=.08; *R*^2^=4.93%), country income level (QM=0.38; *P*=.54; *R*^2^=2.64%), and intervention purpose (QM=1.44; *P*=.25; *R*^2^=11.19%). As for motor symptoms, mean age of participants (QM=4.22; *P*=.04; *R*^2^=2.96%) and supervision mode (QM=1.50; *P*=.22; *R*^2^=3.96%) may explain part of the heterogeneity, with a total *R*^2^=9.42% (*P*=.04). Supervision mode (QM=1.41; *P*=.25; *R*^2^=10.38%) and mean age (QM=0.90; *P*=.35; *R*^2^=.64%) moderated the effect of DHIs on quality of life with a total *R*^2^ of 14.56% (*P*=.14). These meta-regression results should be interpreted as exploratory. The low *R*^2^ values indicate that the factors account for only a small proportion of the total heterogeneity. It reminds us that the variability in intervention effects is likely due to a complex combination of clinical and methodological factors not fully captured here. Nevertheless, these findings generate valuable hypotheses for future research.

### Reach, Fidelity, and Feasibility of DHIs

Most studies reported their eligible population and participant enrollment (Multimedia Appendix 13). A total of 94 studies reported on intervention reach with a median reach of 37.5% (range 4.5%-94.7%), and all included studies reported the randomly assigned populations, with a median of 50% (range 30.6%-100%) randomly assigned to the intervention groups. A total of 38 studies reported intervention fidelity, revealing a high degree of fidelity across a diverse range, of which 34 reported percentage fidelity with a median of 95.7% (range 26.7%-100%), and another 4 studies provided relevant information regarding fidelity. Dropout rates were reported in 105 studies, with a median of 9.1% (range 0%-61.1%).

A total of 32 studies reported that the interventions were feasible to deliver with high satisfaction, adoption, preference for interventions, high interest, high retention rates, convenience, high acceptance, reliability, safety, comfort, flexibility, high adherence, cost-effectiveness, user-friendliness, high recruitment, and high fidelity. Only 5 of these reports were from LMICs. A total of 35 studies did not mention feasibility directly, but they reported a high rate of usability, no serious adverse events, favorable satisfaction ratings, willingness to recommend to others, low cost, high participant interactivity, and low dropout rate. The remaining 44 studies did not report any data on feasibility.

## Discussion

In contrast to previous reviews focusing on single DHI modalities or specific symptoms, this is the first systematic review and meta-analysis to comprehensively evaluate the effectiveness of all existing DHIs across motor symptoms, nonmotor symptoms, and quality of life in patients with PD, while providing a concurrent analysis of implementation feasibility. In this review, we systematically evaluated the effectiveness and implementation of currently reported DHIs in patients with PD, collecting data from 97 RCTs, including 4404 participants from 24 countries. Favorable postintervention effects of DHIs were found over control groups on motor symptoms and specific nonmotor domains (cognitive function, psychiatric symptoms, and overall burden), with the improvement of motor symptoms and cognitive function stable at follow-up assessments. However, substantial and unexplained heterogeneity was detected, and studies with a moderate to high risk of bias accounted for a high proportion; hence, the GRADE approach rated the overall certainty of evidence as low to very low. The wide 95% PIs indicate substantial heterogeneity, suggesting that the true effect remains uncertain. Future implementations might find DHIs produce strong benefits in some populations, yet be ineffective or even worse than control in others, with the results spanning from no effect to a clear positive or negative outcome. In addition, a high proportion of the included studies were assessed as having moderate to high risk of bias, which calls for more rigorously designed studies in the future. Therefore, clinicians should interpret these findings with caution. Additionally, we summarized the reach, fidelity, and feasibility of DHIs, which is valuable for the design of future trials as well as clinical decision-making regarding the application of DHIs. This review brings to the field an updated and integrative evidence that can inform clinical decision-making and guide the design of future DHIs. In real-world implications, our findings highlight that while DHIs hold promise for scalable and accessible PD management, their clinical application should be cautious, personalized, and supported by further high-quality evidence, especially in underserved regions.

Previously reported meta-analyses of the use of DHIs in PD mostly focus on the effects of virtual reality technology [159-167], and others focused on telemedicine [168-170], computerized cognitive training [20], robot-assisted training [112,171-174], mobile app [109,175], and wearable technology [51,176,177]. A recent umbrella review including 8 meta-analyses showed that virtual reality training significantly improved motor performance, mainly including balance ability and stride length in patients with PD [178]. A meta-analysis found that robot-assisted gait training was helpful for the improvement of motor function and balance function [179], while another showed that the efficacy was very uncertain [180]. All of the above meta-analyses were based on a specific type of technology, and their outcomes focused primarily on improvements in motor symptoms. A recent network meta-analysis compared 4 technology-based interventions (internet-based, proprioceptive, robot, and virtual reality) and found that the virtual reality–based intervention fares the best in terms of improving motor symptoms and quality of life [181]. Notably, no study in the field of PD has included all RCTs that met the definition of DHIs and conducted a systematic synthesis. With technological advancements leading to a rapid expansion of DHI applications in PD, a comprehensive analysis of existing DHI modalities in PD is essential to establish an evidence-based framework for guiding future development.

Our review included all types of DHIs, systematically categorizing them by type, purpose, and implementation characteristics while focusing on the 5 key health outcomes in PD. Regarding motor symptoms and cognitive function, our findings are consistent with most previous meta-analyses that DHIs are effective compared with control groups. In addition, we identified significant small-study effects, a critical issue that has not been reported in prior studies. This may reflect publication bias, whereby smaller studies with null results remain unpublished; genuine heterogeneity between smaller and larger trials is also one of the important sources of small-study effects. We used the trim-and-fill method to estimate and adjust for the number and outcomes of missing studies, and the postintervention effects on both motor symptoms and cognitive function became statistically insignificant. This implies that small-study effects might have an impact on these outcomes, and the wide 95% PIs emphasize the uncertainty of the benefits of DHIs. These results highlight the need for further high-quality studies with larger sample sizes and rigorous designs to provide more definitive evidence for updated meta-analyses. For psychiatric symptoms, although the result was statistically significant, it must be interpreted with caution due to the very low certainty of evidence according to the GRADE framework. This low rating may be driven by several factors: the wide 95% PIs, substantial heterogeneity, and a high risk of bias across the included studies.

As for overall nonmotor symptoms, although the results were statistically significant, they should be considered with caution due to the small sample size. Notably, So et al [134] and Dhamija et al [152] were the only 2 trials among 8 to report significant improvements in nonmotor symptoms, and omitting either of them would result in a null effect of the result. It is potentially attributable to its multimodal design (face-to-face education, telecounseling, and smartphone or wearable tools) and early-stage PD cohort. Hence, we did not use the results as a point of reference. In terms of quality of life, unlike previous studies, we did not find a significant improvement. It may be because the majority of scales measuring quality of life are self-reported, such as the most widely used Parkinson’s Disease Questionnaire-39. It lacks high sensitivity for detecting mild to moderate improvements in quality of life, particularly for patients with PD with milder symptoms (Hoehn and Yahr stage ≤3) [182]. In addition, most studies tend to consider quality of life as a secondary outcome, which may affect the design of DHIs. Moreover, complex or long-term interventions may impose an additional burden on patients. Standardized quality of life instruments evaluate a multidimensional construct including emotional well-being, social support, and communication abilities, but the most widely discussed applications of DHIs in PD were motor or cognitive training. Therefore, improvements in isolated domains (eg, mobility or cognitive function) may not substantially enhance overall quality of life. Future DHIs should adopt a user-centered design approach to improve accessibility, reduce treatment-related burdens, and optimize patient experience.

Of the included studies, the majority reported reach or fidelity, and some researchers further reported the feasibility of the intervention. Nearly half of the researchers mentioned that the intervention was feasible and provided quantitative data and qualitative interpretation. This implies that researchers are consciously valuing the feasibility of their interventions. However, the existing problem is the lack of a widely accepted framework for evaluating them, which makes comparing the feasibility of different DHIs across studies challenging. In this review, when analyzing feasibility, we extracted data on reach, fidelity, and dropout rates and summarized the authors’ definitions and explanations related to the feasibility of DHIs as mentioned in the study. For those not directly mentioning feasibility, we also extracted information that may be relevant, including satisfaction, adverse effects, user-friendliness, and so on. Some studies reported high safety with few adverse events [61,85,114,119,133,149], as DHIs that provide real-time surveillance of patients can detect unexpected conditions promptly. Furthermore, physical therapy that incorporates digital technology can help prevent falls and freezing of gait [22] during the training process, such as through the use of robot-assisted gait training [179,183]. The reasons for patients dropping out may include the complexity of the intervention system, and face-to-face guidance before DHIs may increase adherence [184]. Future studies should take into account the aforementioned factors to design more feasible intervention protocols.

The included studies exhibited substantial heterogeneity. The primary source of heterogeneity may be that all DHIs were comprehensively summarized rather than a specific type. The types of DHIs range from the simplest intervention designs of only watching relevant videos to more complex designs involving specifically developed robotic devices and even using multiple digital tools simultaneously. Therefore, we conducted subgroup analyses according to the intervention type, yet substantial heterogeneity persisted within subgroups. Although only the technology-based rehabilitation devices subgroup of the 3 subgroups showed a statistically significant pooled effect on motor symptoms, the test for subgroup differences was not significant; therefore, these findings should not be interpreted as evidence that this subgroup is superior to the other 2 subgroups. Moreover, while the original RCTs may demonstrate that a specifically designed DHI is superior to the control group, they do not imply that individuals using different DHIs across trials were randomized. Therefore, such cross-trial comparisons are invalid for establishing causal relationships between different types of DHIs. Consequently, observed subgroup effects may reflect differences in study populations, intervention intensity, comparators, durations, or outcome instruments across studies, rather than genuine differences between the DHIs themselves. Meta-regression revealed that the percentage of female participants, mean age, intervention setting, publication year, country income level, intervention purpose, and supervision mode might explain a modest portion of the *I*^2^ heterogeneity. While we cannot fully account for all heterogeneity, this finding suggests that the effectiveness of DHIs may vary depending on individual patient characteristics and supervision mode. Therefore, comprehensive consideration of these factors is essential when selecting DHIs for patients. Moreover, the 95% PIs for every primary outcome were wide and consistently crossed the null value. This finding shows substantial heterogeneity and indicates that the effect of DHIs in future settings could range from being less effective than the control condition to being clearly beneficial due to this clinical and methodological diversity. It reminds us that the same intervention may not work equally well for every patient. Hence, clinicians should consider patient responses individually, and future research could aim to identify which types of DHIs are most suitable for specific patient groups.

Clinicians should interpret our findings and their applicability to local settings with caution, as 75% of participants were recruited from high-income countries. The effectiveness and feasibility of these DHIs may differ substantially in LMICs due to variations in resources, infrastructure, and cultural context. Future research in LMICs is critically needed to provide evidence that is generalizable to these settings. As a majority of the world’s population has access to mobile technologies [185], the implementation of digital technology in LMICs could potentially help to close the treatment gap [186,187].

Strengths of this review include that only RCTs were included, which are of high evidence quality levels; the review was conducted strictly by the methodology outlined in the Cochrane guidelines; a large number of studies were included, enrolling a large population from 24 countries; the review encompassed a broad range of outcomes, including motor symptoms, overall nonmotor symptoms, cognitive functions, psychiatric symptoms, and quality of life; the Hartung-Knapp-Sidik-Jonkman method was applied to obtain more conservative results; and the 95% PI was provided to estimate the potential true effect.

This review also has limitations. First, this review comprehensively included various types of DHIs for analysis, leading to high heterogeneity in the analysis. We explored heterogeneity through prespecified subgroup analyses and meta-regression and identified 3 variables accounting for the heterogeneity significantly, although to a small degree. Second, we included a large number of studies, which resulted in a longer period from literature search to manuscript preparation. Therefore, secondary and third screenings were conducted to ensure that as many recent studies as possible were comprehensively included. In addition, we observed possible publication bias in the analyses; more rigorous studies are needed to confirm these findings.

In summary, this systematic review provided the first comprehensive synthesis of evidence across all DHI types for PD, which distinguishes it from previous reviews that focused on single technologies or specific outcomes. DHIs were associated with improvement in motor symptoms, overall nonmotor symptoms, cognitive function, and psychiatric symptoms, but not with quality of life, compared with the non-DHI group. This study supports the potential of DHIs as a promising nonpharmacological intervention for PD. It brings to the field an integrated evidence base that can guide the design of future research and inform the careful implementation of DHIs in clinical practice. However, in real-world implications, the conclusions should be interpreted with caution given the wide 95% PIs, relatively high risk of bias, small-study effects, and the very-low GRADE ratings. Therefore, more well-designed DHIs and high-quality research are urgently needed, especially in underresourced regions.

## Data Availability

The datasets generated or analyzed during this study are available from the corresponding author on reasonable request.

## Appendix

Multimedia Appendix 1 PRISMA 2020 checklist.

Multimedia Appendix 2 PRISMA-S checklist.

Multimedia Appendix 3 Deviations from the original protocol in the final review.

Multimedia Appendix 4 Search strategies per database.

Multimedia Appendix 5 Outcome measurement tools utilized in individual studies.

Multimedia Appendix 6 Characteristics of included studies.

Multimedia Appendix 7 Risk of bias for included effect estimates based on the Cochrane risk-of-bias tool for randomized trials (RoB2).

Multimedia Appendix 8 Pairwise Comparisons of Intervention Type Subgroups.

Multimedia Appendix 9 Meta-analysis of digital health interventions' efficacy on follow-up motor symptoms, psychiatric symptoms, cognitive function, and quality of life.

Multimedia Appendix 10 Publication bias analyses – Funnel plots and trim and fill analyses at post-intervention assessments on motor symptoms, psychiatric symptoms, cognitive function, overall non-motor symptoms and quality of life.

Multimedia Appendix 11 Meta-regression analyses of the effect on motor symptoms, quality of life, cognitive function and psychiatric symptoms.

Multimedia Appendix 12 Bubble plots for the continuous variables in meta-regression.

Multimedia Appendix 13 Reach, uptake, dropout rate, and feasibility of included interventions.

## Abbreviations

DHI: digital health intervention
GRADE: Grading of Recommendations Assessment, Development, and Evaluation
LMIC: low- and middle-income country
PD: Parkinson disease
PI: prediction interval
PICOS: Population, Intervention, Comparison, Outcomes, and Study Type
PRISMA: Preferred Reporting Items for Systematic Reviews and Meta-Analyses
PRISMA-S: Preferred Reporting Items for Systematic Reviews and Meta-Analyses Literature Search Extension
QM: model Q statistic
RCT: randomized controlled trial
SMD: standardized mean difference
