# Supplementary material for: The Effects of Digital Health Interventions on Motor Symptoms, Nonmotor Symptoms, and Quality of Life in Patients With Parkinson Disease: Systematic Review and Meta-Analysis of Randomized Controlled Trials
Source: J Med Internet Res. 2026 Mar 12;28:e79935. doi: 10.2196/79935 (PMC13147926; doi:10.2196/79935)
Supplement: Multimedia Appendix 3 [file jmir_v28i1e79935_app3.docx]

**Multimedia Appendix 2. Deviations from the original protocol in the final review**

|  | **Original registration** | **Deviation** | **Explanation** |
| --- | --- | --- | --- |
| control | Comparators are traditional, non-technological caregiving methods, including standard care programs, assistants, therapies, or drug treatments. Studies comparing different modes of digital health interventions will also be included. | Comparators are traditional, non-technological caregiving methods, including standard care programs, assistants, therapies, or drug treatments. Studies only comparing different modes of digital health interventions will be excluded. | During the data extraction process, we found that we needed non-DHI data to explore whether DHI could be a better option in the treatment and rehabilitation of PD patients. |
| Main outcome | Any health-related outcomes will be assessed in the current study including improvements in motor functions, cognitive functions, psychiatric symptoms, quality of life, and overall patient well-being, etc. | health-related outcomes including improvements in motor symptoms, cognitive functions, psychiatric symptoms, quality of life, and non-motor symptoms. | During the data extraction process, we classified the results of the majority of studies as the above five outcomes. |
| Strategy for data  synthesis | We will perform data synthesis after categorizing the digital healthy interventions into different groups according to their types or purposes. Heterogeneity among studies was evaluated using Cochran’s Q test and the I² statistic, with an I² less than 25%, 25% to 50%, and greater than 50% indicating low, moderate, and high heterogeneity, respectively. The fixed effect model (Mantel-Haenszel) was applied to calculate pooled estimates among studies when low and moderate heterogeneity exists. When substantial heterogeneity existed, the random-effect model was preferred. Sensitivity analysis was conducted to evaluate whether the overall results were affected statistically and significantly by one individual study. This study will employ funnel plots to present the results and Egger regression intercept to determine publication bias. | Pairwise meta-analyses were performed using standardized mean differences (SMDs, Hedges’ g) calculated from within-group differences (baseline to post-intervention). The random-effect model with DerSimonian-Laird estimator was applied for pooled estimates. Heterogeneity among studies was evaluated using Cochran’s Q test and the I² statistic, with an I² less than 25%, 25% to 50%, and greater than 50% indicating low, moderate, and high heterogeneity, respectively.. Univariate meta-regression with mixed-effect models was conducted to identify moderators of heterogeneity across intervention types (technology-based rehabilitation devices/online classes/databases). Subgroup analysis by intervention type and sensitivity analysis via the leave-one-out method were implemented. Funnel plots with Egger's test assessed publication bias. | The fixed-effect model (Mantel-Haenszel) was replaced with a random-effect model using the DerSimonian-Laird estimator to address anticipated substantial heterogeneity. Standardized mean differences (SMDs, Hedges’ g) derived from within-group baseline-to-post-intervention differences were formally adopted to standardize effect sizes. Univariate mixed-effects meta-regression was introduced to systematically identify moderators of heterogeneity, and subgroup analysis was explicitly stratified by intervention types (technology-based rehabilitation devices, online classes, digital databases). Sensitivity analysis was refined to the leave-one-out method for robustness validation. These adjustments aimed to enhance analytical rigor in handling variability inherent to diverse digital health interventions. |
