# Supplementary material for: The Effects of Digital Health Interventions on Motor Symptoms, Nonmotor Symptoms, and Quality of Life in Patients With Parkinson Disease: Systematic Review and Meta-Analysis of Randomized Controlled Trials
Source: J Med Internet Res. 2026 Mar 12;28:e79935. doi: 10.2196/79935 (PMC13147926; doi:10.2196/79935)
Supplement: Multimedia Appendix 4 [file jmir_v28i1e79935_app4.docx]

**Multimedia Appendix 3. Search strategies per database.**

| ***PUBMED*** |  | *"Dementia"[MeSH Terms] OR "Cognition Disorders"[MeSH Terms] OR "Neurocognitive Disorders"[MeSH Terms] OR "Alzheimer Disease"[MeSH Terms] OR "cognitive dysfunction"[MeSH Terms] OR "lewy body disease"[MeSH Terms] OR "**dementia, vascular"[MeSH Terms] OR "Dementia"[Title/Abstract] OR "Dementias"[Title/Abstract] OR "alzheimer s disease"[Title/Abstract] OR "alzheimer dementias"[Title/Abstract] OR "cognitive impairment"[Title/Abstract] OR "mild cognitive impairment"[Title/Abstract] OR "cognitive decline"[Title/Abstract] OR "neurocognitive disorder"[Title/Abstract] OR "cognitive disorder"[Title/Abstract] OR "parkinson s disease dementia"[Title/Abstract] OR "vascular dementia"[Title/Abstract] OR "Parkinson Disease"[MeSH Terms] OR "parkinson s disease"[Title/Abstract] OR "Parkinson Disease"[Title/Abstract] OR "Parkinson"[Title/Abstract] OR "Parkinsonism"[Title/Abstract] OR "paralysis agitans"[Title/Abstract] OR "PD"[Title/Abstract]* |
| --- | --- | --- |
|  | AND | *"technology"[MeSH Terms] OR "telemedicine"[MeSH Terms] OR "internet"[MeSH Terms] OR "robotics"[MeSH Terms] OR "virtual reality"[MeSH Terms] OR "mobile applications"[MeSH Terms] OR "exergaming"[MeSH Terms] OR "video games"[MeSH Terms] OR "Computers"[MeSH Terms] OR "Digital Technology"[MeSH Terms] OR "Smartphone"[MeSH Terms] OR "Software"[MeSH Terms] OR "Online Systems"[MeSH Terms] OR "Internet-Based Intervention"[MeSH Terms] OR "Medical Informatics Applications"[MeSH Terms] OR "Wearable Electronic Devices"[MeSH Terms] OR* *"computers, handheld"[MeSH Terms] OR "web-based"[Title/Abstract] OR "tele*"[Title/Abstract] OR "computer*"[Title/Abstract] OR "robo*"[Title/Abstract] OR "Smartphone"[Title/Abstract] OR "application*"[Title/Abstract] OR "app"[Title/Abstract] OR "apps"[Title/Abstract] OR "application*"[Title/Abstract] OR "iPad"[Title/Abstract] OR "game*"[Title/Abstract] OR "exergam*"[Title/Abstract] OR "digital"[Title/Abstract] OR "DTx"[Title/Abstract] OR "wearable"[Title/Abstract] OR "mhealth"[Title/Abstract] OR "ehealth"[Title/Abstract] OR "mobile"[Title/Abstract] OR "phone"[Title/Abstract] OR "message*"[Title/Abstract] OR "internet*"[Title/Abstract] OR "videoconferenc*"[Title/Abstract] OR "Software"[Title/Abstract] OR "online"[Title/Abstract] OR "tablet"[Title/Abstract] OR "television"[Title/Abstract] OR "TV"[Title/Abstract] OR "TV-based"[Title/Abstract] OR "home-based"[Title/Abstract] OR "technolog*"[Title/Abstract] OR "multimedia"[Title/Abstract] OR "sensor*"[Title/Abstract] OR "virtual"[Title/Abstract] OR "VR"[Title/Abstract] OR "video*"[Title/Abstract] OR "remote*"[Title/Abstract]* |
|  | AND | *"Randomized Controlled Trial"[Publication Type] OR "Randomized Controlled Trial"[ Title/Abstract] OR "Randomized"[ Title/Abstract] OR "Randomised"[ Title/Abstract] OR "Randomization"[ Title/Abstract] OR "Randomisation"[ Title/Abstract]* |
| ***EMBASE*** |  | *'dementia'/exp OR 'Alzheimer disease'/exp OR 'diffuse Lewy body disease'/exp OR 'mild cognitive impairment'/exp OR 'Parkinson disease'/exp OR 'parkinsonism'/exp OR cognitive impairment*:ti,ab,kw OR 'neurocognitive disorder':ti,ab,kw OR dementia*:ti,ab,kw OR alzheimer*:ti,ab,kw OR parkinson*:ti,ab,kw OR 'lewy body':ti,ab,kw* |
|  | *AND* | *'technology'/exp OR 'telemedicine'/exp OR 'Internet'/exp OR 'robotics'/exp OR 'virtual reality'/exp OR 'mobile application'/exp OR 'exergaming'/exp OR 'video game'/exp OR 'computer'/exp OR 'digital technology'/exp OR 'smartphone'/exp OR 'software'/exp OR 'online system'/exp OR 'web-based intervention'/exp OR 'wearable device'/exp OR 'personal digital assistant'/exp OR 'healthcare software'/exp OR 'telehealth'/exp OR 'mobile phone'/exp OR 'television'/exp OR 'sensor'/exp OR ' tele*':ti,ab,kw OR computer*:ti,ab,kw OR robo*:ti,ab,kw OR Smartphone:ti,ab,kw OR iPad:ti,ab,kw OR app:ti,ab,kw OR apps:ti,ab,kw OR exergam*:ti,ab,kw OR digital:ti,ab,kw OR DTx:ti,ab,kw OR wearable:ti,ab,kw OR mhealth:ti,ab,kw OR ehealth:ti,ab,kw OR mobile:ti,ab,kw OR phone:ti,ab,kw OR message:ti,ab,kw OR internet:ti,ab,kw OR videoconferenc*:ti,ab,kw OR Software:ti,ab,kw OR online:ti,ab,kw OR tablet:ti,ab,kw OR television OR TV-based:ti,ab,kw OR home-based:ti,ab,kw OR technology:ti,ab,kw OR virtual:ti,ab,kw OR remote:ti,ab,kw* |
|  | *AND* | *‘Randomized Controlled Trial’ :ti,ab,kw OR Randomized:ti,ab,kw OR Randomised:ti,ab,kw OR Randomization:ti,ab,kw OR Randomisation:ti,ab,kw* |
|  | *AND* | *[humans]/lim AND [embase]/lim* |
| ***Cochrane library trials*** |  | *MeSH descriptor: [Dementia] explode all trees OR MeSH descriptor: [Cognition Disorders] explode all trees OR MeSH descriptor: [Neurocognitive Disorders] explode all trees OR MeSH descriptor: [Alzheimer Disease] explode all trees OR MeSH descriptor: [Cognitive Dysfunction] explode all trees OR MeSH descriptor: [Lewy Body Disease] explode all trees OR MeSH descriptor: [Dementia, Vascular] explode all trees OR MeSH descriptor: [Parkinson Disease] explode all trees OR MeSH descriptor: [Parkinsonian Disorders] explode all trees OR* *cognitive NEXT impairment*:ti,ab,kw OR "neurocognitive disorder":ti,ab,kw OR dementia*:ti,ab,kw OR alzheimer*:ti,ab,kw OR parkinson*:ti,ab,kw OR "cognitive disorder":ti,ab,kw OR "paralysis agitans":ti,ab,kw OR "lewy body":ti,ab,kw* |
|  | AND | *MeSH descriptor: [Technology] explode all trees OR MeSH descriptor: [Telemedicine] explode all trees OR MeSH descriptor: [Internet] explode all trees OR MeSH descriptor: [Robotics] explode all trees OR MeSH descriptor: [Virtual Reality] explode all trees OR MeSH descriptor: [Mobile Applications] explode all trees OR MeSH descriptor: [Exergaming] explode all trees OR MeSH descriptor: [Video Games] explode all trees OR MeSH descriptor: [Computers] explode all trees OR MeSH descriptor: [Digital Technology] explode all trees OR MeSH descriptor: [Smartphone] explode all trees OR MeSH descriptor: [Software] explode all trees OR MeSH descriptor: [Online Systems] explode all trees OR MeSH descriptor: [Internet-Based Intervention] explode all trees OR MeSH descriptor: [Medical Informatics Applications] explode all trees OR MeSH descriptor: [Wearable Electronic Devices] explode all trees OR MeSH descriptor: [computers, handheld] explode all trees OR* *'tele*':ti,ab,kw OR computer*:ti,ab,kw OR robo*:ti,ab,kw OR Smartphone:ti,ab,kw OR iPad:ti,ab,kw OR “app”:ti,ab,kw OR “apps”:ti,ab,kw OR exergam*:ti,ab,kw OR digital:ti,ab,kw OR DTx:ti,ab,kw OR wearable:ti,ab,kw OR mhealth:ti,ab,kw OR ehealth:ti,ab,kw OR mobile:ti,ab,kw OR phone:ti,ab,kw OR message*:ti,ab,kw OR internet:ti,ab,kw OR videoconferenc*:ti,ab,kw OR Software:ti,ab,kw OR online:ti,ab,kw OR tablet:ti,ab,kw OR TV-based:ti,ab,kw OR television:ti,ab,kw OR home-based:ti,ab,kw OR technology:ti,ab,kw OR virtual:ti,ab,kw OR remote:ti,ab,kw* |
|  | AND | *MeSH descriptor: [Randomized Controlled Trial] explode all trees OR* *Randomized Controlled Trial:ti,ab,kw OR Randomized:ti,ab,kw OR Randomised:ti,ab,kw OR Randomization:ti,ab,kw OR Randomisation:ti,ab,kw* |
| ***Web of Science*** |  | *TS=( dementia* OR Alzheimer* OR Parkinson* OR "Lewy Body" OR "Cognition Disorder* "OR "cognitive impairment" OR "mild cognitive impairment" OR "vascular dementia" OR PD)* |
|  | AND | *TS=(technolog* OR tele* OR internet OR robo* OR virtual OR VR OR application* OR exergam* OR digital OR Smartphone OR Software OR Online OR Wearable OR computer* OR app OR apps OR iPad OR DTx OR mhealth OR ehealth OR mobile OR phone OR message OR messages OR videoconferenc* OR tablet OR home-based OR remote OR television)* |
|  | AND | *TS=( Randomized Controlled Trial OR Randomized OR Randomised OR Randomization OR Randomisation)* |
| ***CINAHL Plus with Full Text*** |  | *AB ( dementia* OR alzheimer’s disease* OR cognitive impairment OR cognitive decline OR neurocognitive disorder OR cognitive disorder OR Parkinson* OR Parkinson’s disease OR paralysis agitans OR PD ) OR TI ( dementia* OR alzheimer’s disease* OR cognitive impairment OR cognitive decline OR neurocognitive disorder OR cognitive disorder OR Parkinson* OR Parkinson’s disease OR paralysis agitans OR PD ) OR ((MH "Dementia") OR (MH "Dementia, Vascular") OR (MH "Lewy Body Disease") OR (MH "Dementia Patients") OR (MH "Alzheimer's Disease") OR (MH "Cognition Disorders") OR (MH "Mild Cognitive Impairment") OR (MH "Parkinson Disease"))* |
|  | *AND* | *AB (web-based OR tele* OR computer* OR robo* OR smartphone OR application* OR app* OR iPad OR health software OR game* OR exergam* OR digital OR DTx OR wearable OR mhealth OR ehealth OR mobile OR phone OR message* OR internet* OR videoconferenc* OR software OR health app* OR online OR tablet OR television OR TV* OR technolog* OR multimedia OR sensor* OR virtual OR VR OR video* OR electronic OR remote*) OR TI (web-based OR tele* OR computer* OR robo* OR smartphone OR application* OR app* OR iPad OR health software OR game* OR exergam* OR digital OR DTx OR wearable OR mhealth OR ehealth OR mobile OR phone OR message* OR internet* OR videoconferenc* OR software OR health app* OR online OR tablet OR television OR TV* OR technolog* OR multimedia OR sensor* OR virtual OR VR OR video* OR electronic OR remote*) OR ((MH "Technology") OR (MH "Telemedicine") OR (MH "Telehealth") OR (MH "Telerehabilitation") OR (MH "Internet") OR (MH "Internet-Based Intervention") OR (MH "Robotics") OR ((MH "Virtual Reality") OR (MH "Virtual Reality Exposure Therapy") OR (MH "Mobile Applications") OR (MH "Exergames") OR (MH "Video Games+") OR (MH "Computers, Portable") OR (MH "Digital Technology") OR (MH "Digital Health") OR (MH "Software") OR (MH "Online Systems") OR (MH "Text Messaging+") OR (MH "Computers, Hand-Held+") OR (MH "Medical Informatics"))* |
|  | *AND* | *(DE "Randomized Controlled Trials" OR DE "Randomized Clinical Trials") OR TI randomi* OR AB randomi** |
| *APA PsycInfo* |  | *(DE "Dementia" OR DE "Alzheimer's Disease" OR DE "Dementia with Lewy Bodies" OR DE "Vascular Dementia" OR DE "Cognitive Impairment" OR DE "Mild Cognitive Impairment" OR DE "Parkinson's Disease" OR DE "Parkinsonism" ) OR AB ( dementia* OR alzheimer’s disease* OR cognitive impairment OR cognitive decline OR neurocognitive disorder OR cognitive disorder OR Parkinson* OR Parkinson’s disease OR paralysis agitans OR PD ) OR TI ( dementia* OR alzheimer’s disease* OR cognitive impairment OR cognitive decline OR neurocognitive disorder OR cognitive disorder OR Parkinson* OR Parkinson’s disease OR paralysis agitans OR PD )* |
|  | *AND* | *(DE "Technology" OR DE "Information and Communication Technology" OR DE "Health Information Technology" OR DE "Mobile Technology" OR DE "Human Technology Interaction" OR DE "Touchscreen Technology" OR DE "Digital Technology" OR DE "Wireless Technologies" OR DE "Wearable Devices" OR DE "Telemedicine" OR DE "Online Therapy" OR DE "Teleconferencing" OR DE "Teleconsultation" OR DE "Telepsychiatry" OR DE "Telepsychology" OR DE "Telerehabilitation" OR DE "Internet" OR DE "Internet Usage" OR DE "Digital Interventions" OR DE "Online Therapy" OR DE "Digital Gaming" OR DE "Smartphones" OR DE "Mobile Applications" OR DE "Robotics" OR DE "Social Robotics" OR DE "Sensor Technology" OR DE "Human Robot Interaction" OR DE "Humanoid Robots" OR DE "Virtual Reality" OR DE "Virtual Reality Exposure Therapy" OR DE "Computer Games" OR DE "Computer Assisted Instruction" OR DE "Computer Usage" OR DE "Computer Software" OR DE "Digital Video" OR DE "Digital Technology" OR DE "Digital Computers" OR DE "Text Messaging" OR DE "Messages" OR DE "Electronic Communication")* |
|  | *AND* | *( DE "Randomized Controlled Trials" OR DE "Randomized Clinical Trials" ) OR TI randomi* OR AB randomi** |
