# Supplementary material for: The Effects of Digital Health Interventions on Motor Symptoms, Nonmotor Symptoms, and Quality of Life in Patients With Parkinson Disease: Systematic Review and Meta-Analysis of Randomized Controlled Trials
Source: J Med Internet Res. 2026 Mar 12;28:e79935. doi: 10.2196/79935 (PMC13147926; doi:10.2196/79935)
Supplement: Multimedia Appendix 5 [file jmir_v28i1e79935_app5.docx]

**Multimedia Appendix 4. Outcome measurement tools utilized in individual studies.**

|  | **Outcome** | | | | |
| --- | --- | --- | --- | --- | --- |
|  | **Motor symptoms** | **Cognition function** | **Psychiatric symptoms** | **non-motor symptoms** | **Quality of life** |
| Albert, 2023 | ^a^UPDRS part Ⅲ | - | - | - | - |
| Allen, 2017 | ^b^NHPT | ^c^MoCA | - | - | ^d^PDQ-39 |
| Bartolo, 2024 | UPDRS part Ⅲ | - | - | - | - |
| Beck, 2017 | UPDRS part Ⅲ | MoCA | ^e^GDS | UPDRS partⅠB | PDQ-39 |
| Bernini, 2019 | UPDRS part Ⅲ | MoCA | ^f^BDI | - | ^g^PDQ-8 |
| Bernini, 2021 | - | MoCA | - | - | - |
| Bogosian, 2022 | - | - | ^h^HADS-D | - | - |
| Calabrò, 2019 | ^i^BBS | - | - | - | - |
| Capecci, 2019 | UPDRS part Ⅲ | - | - | - | PDQ-39 |
| Carda, 2012 | UPDRS part Ⅲ | - | ^j^MCS-12 | - | - |
| Carpinella, 2016 | UPDRS part Ⅲ | - | - | - | PDQ-39 |
| Çetin, 2024 | UPDRS part Ⅲ | MoCA | - | - | PDQ-39 |
| Da Silva, 2022 | UPDRS part Ⅲ | - | - | - | PDQ-39 |
| Das, 2024 | UPDRS part Ⅲ | Trail Making test A | - | - | PDQ-39 |
| De Luca, 2019 | - | - | GDS | - | - |
| De, 2025 | Swipe-slide pattern task-Time | - | - | - | - |
| Del Pino, 2023 | UPDRS part Ⅲ | MoCA | - | UPDRS partⅠ | ^k^EQ5D Mobility |
| Dhamija, 2025 | UPDRS part Ⅲ | - | - | Non-Motor Symptoms Scale | PDQ-8 |
| Dobkin, 2020 | - | - | ^l^HAMD | - | - |
| Dobkin, 2021 | - | - | HAMD | - | - |
| Dorsey, 2010 | UPDRS part Ⅲ | MoCA | GDS | - | PDQ-39 |
| Dorsey, 2013 | UPDRS part Ⅲ | - | - | - | PDQ-39 |
| Eldemir, 2023 | UPDRS part Ⅲ | - | - | - | PDQ-8 |
| Ellis, 2019 | ^m^6WMT | - | - | - | PDQ-39 |
| Fellman, 2020 | - | Working Memory Questionnaire | GDS | - | - |
| Feng, 2019 | UPDRS part Ⅲ | - | - | - | - |
| Ferraz, 2018 | 6WMT | - | GDS | - | PDQ-39 |
| Flynn, 2021 | ^n^10WMT | - | - | - | - |
| Furnari, 2017 | UPDRS part Ⅲ | - | GDS | - | - |
| Galli, 2016 | UPDRS part Ⅲ | - | - | - | - |
| Gandolfi, 2017 | BBS | - | - | - | PDQ-8 |
| Giehl, 2020 | - | Working Memory (object identification performance) | - | - | - |
| Ginis, 2016 | UPDRS part Ⅲ | ^o^CTT-A | ^p^SF-36 mental health | - | - |
| Glicia Pedreira, 2013 | - | - | - | - | PDQ-39 |
| Goffredo, 2023 | UPDRS part Ⅲ | - | - | - | - |
| Gryfe, 2022 | UPDRS part Ⅲ | ^q^SCOPA-COG | HADS-D | - | PDQ-39 |
| Gulcan, 2023 | UPDRS part Ⅲ | - | - | - | - |
| Hajebrahimi, 2022 | UPDRS part Ⅲ | MoCA | GDS | - | PDQ-39 |
| Han, 2023 | BBS | - | - | - | - |
| Harpham, 2025 | UPDRS part Ⅲ |  |  |  |  |
| Hashemi, 2022 | NHPT | - | - | - | - |
| Heldman, 2017 | UPDRS part Ⅲ | - | - | UPDRS partⅠB | PDQ-39 |
| Isaacson, 2019 | UPDRS part Ⅲ | - | - | - | PDQ-39 |
| Jäggi, 2023 | ^r^TUG | Go/No-Go test | - | - | - |
| Johnson, 2024 | 6WMT | - | - | - | PDQ-39 |
| Jong-Hoon, 2020 | BBS | - | - | - | - |
| Kashif, 2022 | UPDRS part Ⅲ | - | - | - | - |
| Kashif, 2024 | UPDRS part Ⅲ | - | - | - | - |
| Kawashima, 2022 | UPDRS part Ⅲ | - | - | - | PDQ-39 |
| Khalil, 2017 | UPDRS part Ⅲ | - | - | - | - |
| Kim, 2022 | UPDRS part Ⅲ | - | - | UPDRS partⅠ | - |
| Kraepelien, 2020 | - | - | HADS-D | - | PDQ-8 |
| Lai, 2020 | 6WMT | - | - | - | - |
| Lakshminarayana, 2017 | - | - | HADS-D | ^s^NMSQuest | PDQ-39 |
| Liao, 2015 | ^t^FGA | - | - | - | - |
| Maas, 2024 | - | - | ^u^HADS | - | PDQ-39 |
| Maggio, 2018 | - | ^v^MMSE | - | - | - |
| Maggio, 2024 | - | MoCA | HAMD | - | - |
| Maggio, 2025 |  | MoCA |  |  | PDQ-8 |
| Manor, 2013 | ^w^SDQ | - | - | - | - |
| Maranesi, 2022 | ^x^POMA | - | MCS-12 | - | ^y^SF-12 |
| McGibbon, 2024 | 6WMT | Scales for Outcomes in Parkinson’s-Cognition | - | - | - |
| Meng-Che, 2016 | BBS | - | - | - | - |
| Nieuwboer, 2007 | a composite score of gait and balance UPDRS items | - | - | - | PDQ-39 |
| Nuvolini, 2025 | TUG | MoCA |  |  |  |
| Ophey, 2020 | UPDRS part Ⅲ | Verbal Working Memory | - | - | - |
| Özden, 2021 | BBS | - | SF-36 mental health | - | - |
| París, 2011 | - | MMSE | GDS | - | PDQ-39 |
| Pastana Ramos, 2023 | UPDRS part Ⅲ | - | - | - | PDQ-8 |
| Patel, 2017 | UPDRS part II | - | ^z^ISI | UPDRS partⅠb | PDQ-8 |
| Peacock, 2021 | - | - | - | - | PDQ-39 |
| Picelli, 2012 | UPDRS part Ⅲ | - | - | - | - |
| Picelli, 2013 | 10WMT | - | - | - | - |
| Picelli, 2015 | UPDRS part Ⅲ | - | - | - | - |
| Piers, 2023 | - | - | BDI | - | - |
| Pinto, 2025 |  |  | Generalized Anxiety Disorder 7-item scale |  | PDQ-8 |
| Pompeu, 2012 | UPDRS part II | MoCA | - | - | - |
| Qayyum, 2022 | ^aa^DGI | - | - | - | - |
| Raciti, 2022 | UPDRS part Ⅲ | - | - | - | - |
| Raglio, 2023 | 6WMT | - | - | - | McGill Quality of Life |
| Ribas, 2017 | BBS | - | - | - | - |
| Sale, 2013 | UPDRS part Ⅲ | - | - | - | - |
| Santos, 2019 | BBS | - | - | - | PDQ-39 |
| So, 2023 | - | - | - | Non-Motor Symptoms Scale | PDQ-39 |
| Song, 2018 | New Freezing of Gait Questionnaire | MoCA | - | - | - |
| Spina, 2021 | BBS | - | - | - | PDQ-39 |
| Svaerke, 2022 | - | ^ab^SDMT | HADS | - | PDQ-39 |
| Tayyebi, 2025 |  |  | HADS |  |  |
| Theodoros, 2015 | - | - | - | - | PDQ-39 |
| van Balkom, 2022 | - | MoCA | BDI | - | - |
| van de Weijer, 2020 | - | Global cognition | - | - | - |
| van den Heuvel, 2014 | UPDRS part Ⅲ | - | HADS-D | - | PDQ-39(mobility) |
| Wilkinson, 2016 | UPDRS part Ⅲ | - | GDS | - | - |
| Yang, 2016 | UPDRS part Ⅲ | - | - | - | PDQ-39 |
| Yen, 2011 | ^ac^SOT1 | - | - | - | - |
| Yuan, 2020 | BBS | - | SF-36 mental health | - | - |
| Zoetewei, 2024 | UPDRS part Ⅲ | - | - | - | - |

^a^UPDRS: Unified Parkinson’s Disease Rating Scale;

^b^NHPT: Nine-Hole Peg Test;

^c^MoCA: Montreal Overall Cognitive Assessment;

^d^PDQ-39: Parkinson Disease Questionnaire 39;

^e^GDS: Geriatric Depression Scale;

^f^BDI: Beck Depression Inventory;

^g^PDQ-8: 8-Item Parkinson’s Disease Questionnaire

^h^HADS-D: Hospital Anxiety and Depression Scale - Depression subscale

^i^BBS: Berg Balance Scale

^j^MCS-12: SF-12 mental component score

^k^EQ5D: Euro Quality of Life five-dimension

^l^HAMD: Hamilton Depression Scale

^m^6MWT: 6-Minute Walk Test

^n^10MWT: 10-Minute Walk Test

^o^CTT-A: Color Trail Test A

^p^SF-36: Short Form 36 Health Survey

^q^SCOPA-COG: Scales for Outcomes in Parkinson’s-Cognition

^r^TUG: timed up-and-go test

^s^NMSQuest: Non-Motor Symptoms Questionnaire

^t^FGA: Functional gait assessment

^u^HADS: Hospital Anxiety and Depression Scale

^v^MMSE: Mini-Mental State Examination

^w^SDQ: swallowing disturbances questionnaire

^x^POMA: Tinetti’s performance oriented mobility assessment

^y^SF-12: Short Form 12 Health Survey

^z^ISI: Insomnia Severity Index

^aa^DGI: Dynamic Gait Index

^ab^SDMT: Symbol Digit Modalities Test

^ac^SOT1: single task Sensory Organization Test 1
