# Supplementary material for: The Effects of Digital Health Interventions on Motor Symptoms, Nonmotor Symptoms, and Quality of Life in Patients With Parkinson Disease: Systematic Review and Meta-Analysis of Randomized Controlled Trials
Source: J Med Internet Res. 2026 Mar 12;28:e79935. doi: 10.2196/79935 (PMC13147926; doi:10.2196/79935)
Supplement: Multimedia Appendix 6 [file jmir_v28i1e79935_app6.docx]

**Multimedia Appendix 5: Characteristics of included studies.**

| **Study ID** | **Country** | **Country income level** | **Population (number of participants, age(**^a^**M), women%)** | **Population of** ^b^**IG (number of participants, age (M (**^c^**SD)), women%)** | **Population of** ^d^**CG (number of participants, age (M (SD)), women%)** | **Disease severity (Hoehn and Yahr stage)** | **Study design** | **Intervention** | **Delivery mode** | **Intervention type** | **Intervention purpose** | **Supervision mode** | **Setting** | **Duration (follow-up period)** | **Comparator** | **Outcome** |
| --- | --- | --- | --- | --- | --- | --- | --- | --- | --- | --- | --- | --- | --- | --- | --- | --- |
| Albert, 2023 | America | High income | 47, 69.11 ^e^yrs, 23.4% | 25, 70.0 (6.4) yrs, 12.0% | 22, 68.1 (5.9) yrs, 36.4% | 2.29 | parallel ^f^RCT | Dual-task Augmented Reality Treatment (DART) | ^g^VR and tablet | Technology-based rehabilitation therapy | Physical rehabilitation | Supervised | Home | 8^h^w (8w) | Active control | Motor symptoms |
| Allen, 2017 | Australia | High income | 38, 67.95 yrs, 39.5% | 19, 67.5 (7.3) yrs, 36.8% | 19, 68.4 (8.5) yrs, 42.1% | ^i^NR | parallel RCT | Exergames targeting the upper extremity (UE) | Tablet | Technology-based rehabilitation therapy | Physical rehabilitation | Unsupervised | Home | 12w (^j^NA) | Active control | Motor symptoms, Cognition function, Quality of life |
| Bartolo, 2024 | Italy | High income | 52, 71.65 yrs, 28.8% | 26, 73.0 (7.3) yrs, 34.6% | 26, 70.3(11.0) yrs, 30.0% | 2.1 | parallel RCT | Wearable system for visual cueing gait rehabilitation | Wearable sensors | Technology-based rehabilitation therapy | Physical rehabilitation | Supervised | Medical institution | 2w (12w) | Active control | Motor symptoms |
| Beck, 2017 | America | High income | 195, 66.4 yrs, 46.7% | 97, 65.9 (7.9) yrs, 50.5% | 98, 66.9 (8.5) yrs, 42.8% | NR | parallel RCT | Virtual house calls | Internet-enabled device | Online classes | Care aid | Remotely Supervised (Real-Time) | Home | 48w (NA) | Passive control | Motor symptoms, Cognition function, Psychiatric symptoms, Overall non-motor symptoms, Quality of life |
| Bernini, 2019 | Italy | High income | 35, 70.23 yrs, 51.4% | 17, 71.18 (7.04) yrs, 64.7% | 18, 69.33 (7.72) yrs, 38.9% | 2.85 | parallel RCT | Computer-based cognitive training (CCT) | Computer | Technology-based rehabilitation therapy | Cognitive training | Supervised | Medical institution | 4w (24w) | Active control | Motor symptoms, Cognition function, Psychiatric symptoms |
| Bernini, 2021 | Italy | High income | 30, 72.7 yrs, 36.7% | 18, 74.61 (5.68) yrs, 33.0% | 12, 69.83 (9.66) yrs, 0.4% | NR | parallel RCT | Computer-based cognitive training (CCT) | Computer | Technology-based rehabilitation therapy | Cognitive training | Supervised | Medical institution | 3w (NA) | Active control | Cognition function |
| Bogosian, 2022 | United Kingdom | High income | 60, 60.87 yrs, 50.0% | 30, 59.50 (11.12) yrs, 43.3% | 30, 62.23 (8.96) yrs, 56.7% | NR | parallel RCT | Mindfulness intervention delivered via videoconferencing | Computer | Online classes | Cognitive training | Remotely Supervised (Real-Time) | Home | 8w (12w) | Passive control | Motor symptoms, Psychiatric symptoms |
| Calabrò, 2019 | Italy | High income | 50, 71.5 yrs, 37.5% | 25, 70 (8) yrs, 45.0% | 25, 73 (8) yrs, 0.3% | 3 | parallel RCT | Rhythmic Auditory Stimulation (RAS) gait training | Walking treadmill equipped with rhythmic auditory stimulation | Technology-based rehabilitation therapy | Physical rehabilitation | Supervised | Medical institution | 8w (NA) | Active control | Motor symptoms |
| Capecci, 2019 | Italy | High income | 96, 67.6 yrs, 55.2% | 48, 68.1 (9.8) yrs, 60.0% | 48, 67.0 (7.6) yrs, 0.5% | 3 | parallel RCT | Robot-assisted gait training | Robot-assisted | Technology-based rehabilitation therapy | Physical rehabilitation | Supervised | Medical institution | 4w (NA) | Active control | Motor symptoms, Quality of life |
| Carda, 2012 | Italy | High income | 30, 67.89 yrs, 43.3% | 15, 67.87 (7.05) yrs, NR | 15, 66.93 (5.13) yrs, NR | 2.2 | parallel RCT | Robot-assisted gait training | Robot-assisted | Technology-based rehabilitation therapy | Physical rehabilitation | Supervised | Medical institution | 4w (24w) | Active control | Motor symptoms, Psychiatric symptoms |
| Carpinella, 2016 | Italy | High income | 37, 74.41 yrs, 37.8% | 17, 73.0 (7.1) yrs, 17.6% | 20, 75.6 (8.2) yrs, 55.0% | 2.81 | parallel RCT | System (Gamepad) for biofeedback rehabilitation | Wearable sensor | Technology-based rehabilitation therapy | Physical rehabilitation | Supervised | NR | 7w (4w) | Active control | Motor symptoms, Quality of life |
| Çetin, 2024 | Türkiye | Upper-middle Income | 20,69.25 yrs, 35.0% | 10, 68.5(NR) yrs, 30% | 10, 70.0(NR)yrs, 40% | 2.58 | parallel RCT | Physiotherapy game system | Exergame by touch screen | Technology-based rehabilitation therapy | Physical rehabilitation and Cognitive training | Supervised | Medical institution | 8w (NA) | Active control | Motor symptoms, Cognition function, Quality of life |
| Constantinescu, 2011 | Australia | High income | 34, 70.12 yrs, 20.6% | 17, 70.65 (10.26) yrs, 17.7% | 17, 69.59 (6.71) yrs, 23.6% | 1.56 | parallel RCT | Online delivery of the Lee Silverman Voice Treatment (LSVT) | Computer | Online classes | Physical rehabilitation | Supervised | Medical institution | 4w (NA) | Active control | None |
| Cubo, 2025 | Spain | High income | 50, 70.2 yrs, 50% | 25, 69.2 (9.4) yrs, 52% | 25, 71.1 (9.0) yrs, 48% | 2 | parallel RCT | Telemedicine | video-conference devices and wearable sensor | Technology-based rehabilitation therapy | Physical rehabilitation | Remotely Supervised (Asynchronous) | Home | 12w (12w) | Passive control | None |
| Da Silva, 2022 | Brazil | Upper-middle Income | 38, 65.79 yrs, 13.2% | 18, 63.33 (6.46) yrs, 22.2% | 20, 68.00 (10.02) yrs, 5.0% | NR | parallel RCT | Training based on Kinect Adventures games (exergame) | VR | Technology-based rehabilitation therapy | Physical rehabilitation | Supervised | Medical institution | 7w (4w) | Active control | Motor symptoms, Quality of life |
| Das, 2024 | United Kingdom | High income | 40, 70.5(13.3) yrs, 25.0% | 20, 71.5(10.74) yrs, 25.0% | 20, 71.5(10.74) yrs, 25.0% | 2.09 | parallel RCT | Visuo-cognitive training | Stroboscopic glasses and tablet | Technology-based rehabilitation therapy | Cognitive training | Remotely Supervised (Real-Time) | Home | 4w (NA) | Active control | Motor symptoms, Cognition function, Quality of life |
| De Luca, 2019 | Italy | High income | 60, 66.5 yrs, 48.3% | 30, 61.9 (11.5) yrs, 46.7% | 30, 63.2 (7.3) yrs, 50.0% | NR | parallel RCT | Computer-assisted cognitive rehabilitation (CACR) | Computer | Technology-based rehabilitation therapy | Cognitive training | Supervised | Medical institution | 8w (NA) | Active control | Psychiatric symptoms |
| de Melo, 2018 | Brazil | Upper-middle Income | 25, 60.64 yrs, 8.0% | 12, 60.25 (9.28) yrs, 8.3% | 13, 61 (10.72) yrs, 7.7% | 1.47 | parallel RCT | Gait training with virtual reality (VR) | VR | Technology-based rehabilitation therapy | Physical rehabilitation | Supervised | Medical institution | 4w (4w) | Active control | None |
| De, 2025 | Belgium | High income | 34, 69.4 yrs, 52.9% | 16, 69.9 (6.5) yrs, 56.2% | 18, 69 (8.0) yrs, 50% | 2.5 | parallel RCT | home-based, unsupervised tablet-task training of Swipe-Slide Pattern (SSP) task | Tablet | Technology-based rehabilitation therapy | Physical rehabilitation | Unsupervised | Home | 2w (4w) | Passive control | Motor symptoms |
| Del Pino, 2023 | Spain | High income | 20, 66.8 yrs, 30.0% | 10, 64.5 (7.9) yrs, 30.0% | 10, 69.1 (3.5) yrs, 0.3% | 2.1 | parallel RCT | Virtual coach and telerehabilitation (vCare system) | Television, wearable sensors and tablet | Technology-based rehabilitation therapy and digital databases | Care aid, physical rehabilitation  and cognitive training | Unsupervised | Home | 16w (NA) | Active control | Motor symptoms, Cognition function, Quality of life, Overall non-motor symptoms |
| Dhamija, 2025 | India | Upper-middle Income | 63, 61.8, 44.4% | 35, 62.80 (12.46) yrs, 45.7% | 28, 60.50 (7.08), 42.8% | 1.86 | parallel RCT | Telerehabilitation | mobile-based videocalling | Technology-based rehabilitation therapy | Physical rehabilitation | Remotely Supervised (Real-Time) | Home | 12w (NA) | Active control | Motor symptoms, Overall non-motor symptoms, Quality of life |
| Dobkin, 2020 | France | High income | 72, 65.22 yrs, 1.4% | 37, 65.62 (9.76) yrs, 54.1% | 35, 64.8 (9.62) yrs, 8.6% | NR | parallel RCT | Telephone-based cognitive-behavioral treatment (T-CBT) | Telephone | Online classes | Care aid and cognitive training | Supervised | NR | 10w (24w) | Active control | Psychiatric symptoms |
| Dobkin, 2021 | France | High income | 90, 66.84 yrs, 0.0% | 45, 67.27 (7.79) yrs, 0.0% | 45, 66.42 (9.51) yrs, 0.0% | NR | parallel RCT | Video-to-home cognitive-behavioral therapy (V-CBT) | Computer/smartphone | Online classes | Care aid and cognitive training | Remotely Supervised (Real-Time) | Home | 10w (24w) | Active control | Psychiatric symptoms |
| Dorsey, 2010 | America | High income | 10, 80.94 yrs, 60.0% | 6, 71.7 (7.9) yrs, 33.0% | 4, 69.8 (7.0) yrs, 1.0% | NR | parallel RCT | Telemedicine | Computer | Online classes | Care aid | Supervised | Medical institution | 24w (NA) | Active control | Motor symptoms, Cognition function, Psychiatric symptoms, Quality of life |
| Dorsey, 2013 | America | High income | 20, 65.45 yrs, 25.0% | 9, 66.6 (12) yrs, 27.3% | 11, 64.5 (11.3) yrs, 22.2% | 2.41 | parallel RCT | Web-based videoconferencing (telemedicine) | Computer | Online classes | Care aid | Remotely Supervised (Real-Time) | Home | 28w (NA) | Active control | Motor symptoms, Quality of life |
| Edwards, 2013 | America | High income | 87, 68.85 yrs, 37.9% | 44, 69.38 (7.81) yrs, 36.4% | 43, 68.17 (8.38) yrs, 39.5% | 2 | parallel RCT | Self-administered cognitive speed of processing training (SOPT)  using InSight software | NR | Technology-based rehabilitation therapy | Cognitive training | Unsupervised | Home | 12w (NA) | Passive control | None |
| Eldemir, 2023 | Türkiye | Upper-middle Income | 30, 59.64 yrs, 36.7% | 15, 57.87 (9.79) yrs, 33.3% | 15, 61.40 (7.29) yrs, 40.0% | 1.93 | parallel RCT | Task-oriented circuit training-based telerehabilitation (TOCT-TR) | Computer | Technology-based rehabilitation therapy | Physical rehabilitation | Remotely Supervised (Real-Time) | Home | 6w (NA) | Active control | Motor symptoms, Quality of life |
| Ellis, 2019 | America | High income | 51, 64.1 yrs, 45.1% | 26, 64.8 (8.5) yrs, 42.3% | 25, 63.3 (10.6) yrs, 48.0% | 2.1 | parallel RCT | Mobile health (mHealth)–mediated exercise program | Tablet | Technology-based rehabilitation therapy | Physical rehabilitation | Remotely Supervised (Asynchronous) | Home | 48w (NA) | Active control | Motor symptoms, Quality of life |
| Fellman, 2020 | Finland | High income | 52, 65.16 yrs, 65.4% | 26, 64.8 (6.2) yrs, 65.4% | 26, 65.5 (4.7) yrs, 65.4% | NR | parallel RCT | Home-based computerized training | Computer | Technology-based rehabilitation therapy | Cognitive training | Unsupervised | Home | 5w (NA) | Active control | Cognition function, Psychiatric symptoms |
| Feng, 2019 | China | Upper-middle Income | 28, 67.2 yrs, 53.3% | 14, 67.47 (4.79) yrs, 46.7% | 14, 66.93 (4.64) yrs, 60.0% | 3 | parallel RCT | Virtual reality rehabilitation | VR | Technology-based rehabilitation therapy | Physical rehabilitation | Supervised | Home | 12w (NA) | Active control | Motor symptoms |
| Ferraz, 2018 | Brazil | Upper-middle Income | 47, 68.7 yrs, 38.1% | 22, 70.6 (7.13) yrs, 50.0% | 25, 67 (1.60) yrs, 27.3% | 2.5 | parallel RCT | Kinect Adventures exergames | VR | Technology-based rehabilitation therapy | Physical rehabilitation | Supervised | Medical institution | 8w (NA) | Active control | Motor symptoms, Psychiatric symptoms, Quality of life |
| Flynn, 2021 | Australia | High income | 40, 72 yrs, 25.0% | 20, 72 (7.3) yrs, 25.0% | 20, 71 (6.6) yrs, 0.3% | 2.11 | parallel RCT | Home-based exercise program monitored using telehealth | NR | Digital databases | Physical rehabilitation | Unsupervised | Home | 10w (NA) | Active control | Motor symptoms |
| Furnari, 2017 | Italy | High income | 38, 77.6 yrs, 44.7% | 19, 71.5 (11.7) yrs, 42.0% | 19, 77.7 (8.3) yrs, 47.3% | 2.6 | parallel RCT | Robotic-assisted gait training | Robot-assisted | Technology-based rehabilitation therapy | Physical rehabilitation | Supervised | Medical institution | 4w (12w) | Active control | Motor symptoms, Psychiatric symptoms |
| Galli, 2016 | Italy | High income | 50, 67.6 yrs, 48.0% | 25, 68.8 (6.9) yrs, 44.0% | 25, 66.4 (9.7) yrs, 3(52% | NR | parallel RCT | Robotic-assisted gait training | Robot-assisted | Technology-based rehabilitation therapy | Physical rehabilitation | Supervised | Medical institution | 4w (NA) | Active control | Motor symptoms |
| Gandolfi, 2017 | Italy | High income | 76, 68.64 yrs, 32.9% | 38, 67.45 (7.18) yrs, 39.5% | 38, 69.84 (9.41) yrs, 26.3% | 2.5 | parallel RCT | Remotely supervised in-home VR balance training | VR | Technology-based rehabilitation therapy | Physical rehabilitation | Supervised | Home | 7w (4w) | Active control | Motor symptoms, Quality of life |
| Giehl, 2020 | Germany | High income | 41, 64.33 yrs, 6.3% | 19, 65.3 (8.9) yrs, 52.6% | 22, 63.5 (9.1) yrs, 40.9% | 2.05 | parallel RCT | Home-based digital working memory training | Computer | Technology-based rehabilitation therapy | Cognitive training | Unsupervised | Home | 5w (NA) | Passive control | None |
| Giehl, 2020 | Germany | High income | 72, 64.13 yrs, 45.8% | 36, 64.36 (8.51) yrs, 47.0% | 36, 63.90 (8.28) yrs, 44.0% | 2.05 | parallel RCT | Home-based digital working memory training | Computer | Technology-based rehabilitation therapy | Cognitive training | Unsupervised | Home | 5w (14w) | Passive control | Cognition function |
| Ginis, 2016 | Belgium | High income | 38, 66.76 yrs, 21.1% | 20, 67.30 (8.13) yrs, 15.0% | 18, 66.11 (8.07) yrs, 27.7% | NR | parallel RCT | Inertial measurement units combined with a smartphone application  (CuPiD-system) | Smartphone and  wearable sensor | Technology-based rehabilitation therapy | Physical rehabilitation | Remotely Supervised (Asynchronous) | Home | 6w (4w) | Active control | Motor symptoms, Cognition function, Psychiatric symptoms |
| Glicia Pedreira, 2013 | Brazil | Upper-middle Income | 32, 63.65 yrs, 29.0% | 16, 61.1 (8.2) yrs, 31.3% | 16, 66.2 (8.5) yrs, 26.7% | 2.45 | parallel RCT | Nintendo Wii training (Virtual games) | VR | Technology-based rehabilitation therapy | Physical rehabilitation | Supervised | Medical institution | 4w (NA) | Active control | Motor symptoms, Quality of life |
| Goffredo, 2023 | Italy | High income | 97, 68 yrs, 47.4% | 49, 67.8 (6.6) yrs, 44.9% | 48, 68.2 (5.8) yrs, 50.0% | 1.91 | parallel RCT | Non-immersive virtual reality-based telerehabilitation | VR | Technology-based rehabilitation therapy | Physical rehabilitation | Remotely Supervised (Asynchronous) | Home | 6-10w (NA) | Active control | Motor symptoms |
| Gryfe, 2022 | Canada | High income | 27, 69.21 yrs, 59.2% | 13, 67.6 (5.9) yrs, 69.2% | 14, 70.7 (7.3) yrs, 50.0% | 2 | parallel RCT | Bilateral exoskeleton (Exo) exercise | Robot-assisted | Technology-based rehabilitation therapy | Physical rehabilitation | Supervised | Medical institution | 8w (NA) | Active control | Motor symptoms, Cognition function, Psychiatric symptoms, Quality of life |
| Gulcan, 2023 | Türkiye | Upper-middle Income | 30, 60.3 yrs, 13.3% | 15, 61.4 (7.4) yrs, 13.3% | 15, 59.2 (13.1) yrs, 13.3% | 2.37 | parallel RCT | VR gait training | VR | Technology-based rehabilitation therapy | Physical rehabilitation | Supervised | Medical institution | 6w (NA) | Active control | Motor symptoms |
| Hajebrahimi, 2022 | Türkiye | Upper-middle Income | 24, 65.9 yrs, 20.8% | 11, 66.36 (8.04) yrs, 18.2% | 13, 65.53 (9.93) yrs, 23.1% | NR | parallel RCT | Virtual reality-based exergaming | VR | Technology-based rehabilitation therapy | Physical rehabilitation | Supervised | Medical institution | 4w (NA) | Active control | Motor symptoms, Cognition function, Psychiatric symptoms, Quality of life |
| Halpern, 2012 | America | High income | 16, 64.85 yrs, 50.0% | 8, 65.8 (8.4) yrs, 50.0% | 8, 63.3 (6.0) yrs, 0.5% | NR | parallel RCT | Intensive Voice Treatment (LSVT®LOUD) | Computer/  Intelligent device | Technology-based rehabilitation therapy | Care aid | Supervised | Medical institution/Home | 4w (24w) | Passive control | None |
| Han, 2023 | China | Upper-middle Income | 48, 57.22 yrs, 52.1% | 24, 58.17 (5.36) yrs, 50.0% | 24, 56.25 (4.82) yrs, 54.2% | 1.36 | parallel RCT | Visual feedback balance training | Computer | Technology-based rehabilitation therapy | Physical rehabilitation | Supervised | Medical institution | 4w (NA) | Active control | Motor symptoms |
| Harpham, 2025 | United Kingdom | High income | 13, 63.4 yrs, 38.5% | 7, 61.8 (9.4) yrs, 42.8% | 6, 65.2 (2.3) yrs, 33.3% | 1.58 | parallel RCT | Home-based high-intensity interval training | Smartphone and online devices | Technology-based rehabilitation therapy | Physical rehabilitation | Remotely Supervised (Asynchronous) | Home | 12w (NR) | Passive control | Motor symptoms |
| Hashemi, 2022 | Iran | Upper-middle Income | 30, 58.94 yrs, 30.0% | 15, 54.80 (10.51) yrs, 40.0% | 15, 61.07 (7.01) yrs, 0.2% | 2.17 | parallel RCT | Upper limb virtual reality exercises (ULVRE) | VR | Technology-based rehabilitation therapy | Physical rehabilitation | Supervised | Medical institution | 8w (8w) | Active control | Motor symptoms |
| Heldman, 2017 | America | High income | 18, 66.9 yrs, 33.3% | 9, 65.2 (10.1) yrs, 44.4% | 9, 68.6 (10.2) yrs, 22.2% | NR | parallel RCT | Motion sensor-based telehealth diagnostics | Wearable sensor and tablet | Technology-based rehabilitation therapy | Care aid | Remotely Supervised (Asynchronous) | Home | 28w (NA) | Active control | Motor symptoms, Overall non-motor symptoms, Quality of life |
| Isaacson, 2019 | America | High income | 39, 68.72 yrs, 56.4% | 19, 67.62 (9.77) yrs, 52.6% | 20, 69.76 (7.16) yrs, 60.0% | NR | parallel RCT | Wearable biosensors | Wearable sensor | Digital databases | Care aid | Remotely Supervised (Asynchronous) | Home | 12w (NA) | Active control | Motor symptoms, Quality of life |
| Jäggi, 2023 | Switzerland | High income | 40, 72.4 yrs, 32.5% | 19, 71.89 (9.09) yrs, 36.8% | 21, 72.86 (10.14) yrs, 28.6% | 3 | parallel RCT | Simultaneous cognitive-motor training in form of exergames | Exergaming device (Dividat Senso) | Technology-based rehabilitation therapy | Physical rehabilitation and Cognitive training | Supervised | Medical institution | 1w (NA) | Active control | Motor symptoms, Cognition function |
| Johnson, 2024 | America | High income | 14, NR, NR | 6, 74(NR) yrs, NR | 8，68(NR) yrs, NR | 1.86 | parallel RCT | Telerehabilitation platform and web-based video conference calls | Tablet, computer, or phone | Technology-based rehabilitation therapy | Physical rehabilitation | Remotely Supervised (Asynchronous) | Home | 4w (NA) | Active control | Motor symptoms, Quality of life |
| Jong-Hoon, 2020 | Korea | High income | 15, 62.8 yrs, 33.3% | 8, 63.38 (5.37) yrs, 37.5% | 7, 62.14 (5.55) yrs, 28.6% | 2.67 | parallel RCT | Balance training using a Wii Fit balance board | Computer/Wii Fit | Technology-based rehabilitation therapy | Physical rehabilitation | Supervised | Medical institution | 8w (NA) | Active control | Motor symptoms |
| Kashif, 2022 | Pakistan | Lower-middle Income | 44, 63.09 yrs, 43.2% | 22, 63.86 (4.57) yrs, 41.0% | 22, 62.32 (4.61) yrs, 5.5% | 2.18 | parallel RCT | Motor imagery combined with virtual reality techniques | VR | Technology-based rehabilitation therapy | Physical rehabilitation | Supervised | Medical institution | 12w (4w) | Active control | Motor symptoms |
| Kashif, 2024 | Pakistan | Lower-middle Income | 40, 62.58 yrs, 42.5% | 20, 63.20(4.85) yrs, 40.0% | 20，61.95(4.85) yrs, 45.5% | NR | parallel RCT | Virtual reality systems and routine physical training | VR | Technology-based rehabilitation therapy | Physical rehabilitation | Supervised | Medical institution | 12w (4w) | Active control | Motor symptoms |
| Kawashima, 2022 | Japan | High income | 15, 76.8 yrs, 73.3% | 7, 77.0 (4.4) yrs, 57.1% | 8, 76.6 (6.3) yrs, 87.5% | 1.4 | parallel RCT | home-based gait training using the wearable Stride Management Assist (SMA) exoskeleton | Robot-assisted | Technology-based rehabilitation therapy | Physical rehabilitation | Supervised | Home | 12w (NA) | Active control | Motor symptoms, Quality of life |
| Kegelmeyer, 2024 | America | High income | 45, 72 yrs, 26.7% | 23, 72(NR) yrs, 43.0% | 22, 72(NR) yrs, 43.0% | 2.14 | parallel RCT | Robotic Walking Device | Robot-assisted | Technology-based rehabilitation therapy | Physical rehabilitation | Supervised | Home | 8w (NA) | Passive control | None |
| Khalil, 2017 | Jordan | Lower-middle Income | 30, 59.5 yrs, 36.7% | 16, 58.4 (13.5) yrs, 25.0% | 14, 60.7 (15.4) yrs, 0.5% | 2.31 | parallel RCT | Home-based exercise program using DVD | DVD | Digital databases | Physical rehabilitation | Unsupervised | Medical institution/Home | 8w (NA) | Active control | Motor symptoms |
| Kim, 2022 | Korea | High income | 44, 68.1 yrs, 70.4% | 22, 68.7 (6.9) yrs, 73.0% | 22, 67.5 (9.3) yrs, 0.7% | 2.83 | parallel RCT | Robot-assisted gait training (RAGT) | Robot-assisted | Technology-based rehabilitation therapy | Physical rehabilitation | Unsupervised | Medical institution | 4w (4w) | Active control | Motor symptoms, Overall non-motor symptoms |
| Kluger, 2023 | America | High income | 379, 74 yrs, 0.8% | 179, 74.4(7.6) yrs, 38.0% | 180, 73.6 (9.1) yrs, 32.2% | NR | crossover RCT | Remote access to a palliative education team | Tablet/telephone | Online classes | Care aid | Remotely Supervised (Real-Time) | Home | 48w (NA) | Active control | None |
| Kraepelien, 2020 | Sweden | High income | 77, 66 yrs, 1.0% | 38, 65.9 (8.5) yrs, 63.0% | 39, 66.1 (9.8) yrs, 0.6% | NR | parallel RCT | Guided individually-tailored internet-based cognitive behavioral therapy (ICBT) | Computer/tablet/smartphone | Technology-based rehabilitation therapy | Cognitive training | Remotely Supervised (Asynchronous) | Home | 10w (NA) | Active control | Psychiatric symptoms, Quality of life |
| Kratz, 2025 | Austria | High income | 19, 68.45 yrs, 52.6% | 10, 68.19(8.43) yrs, 30% | 9, 66.58(7.33) yrs, 77.8% | 2 | parallel RCT | Onlie Lee Silverman Voice Treatment (LSVT®) | PC-based videoconference | Technology-based rehabilitation therapy | Care aid | Remotely Supervised (Real-Time) | Medical institution | 4w (NR) | Active control | None |
| Lai, 2020 | America | High income | 20, 67.1 yrs, 30.0% | 10, 63.4 (10.4) yrs, 30.0% | 10, 70.8 (7.1) yrs, 0.3% | 2.22 | parallel RCT | Tele-monitored home-exercise programmes | Tablet and wearable sensor | Technology-based rehabilitation therapy | Physical rehabilitation | Remotely Supervised (Real-Time) | Home | 8w (NA) | Active control | Motor symptoms |
| Lakshminarayana, 2017 | United Kingdom | High income | 201, 60.31 yrs, 39.3% | 94, 59.86 (9.13) yrs, 36.2% | 107, 60.71 (10.26) yrs, 42.1% | NR | parallel RCT | Smartphone-based Parkinson’s tracker app | Smartphone/tablet/computer | Digital databases | Care aid | Unsupervised | Home | 16w (NA) | Active control | Psychiatric symptoms, Overall non-motor symptoms, Quality of life |
| Lau, 2022 | America | High income | 18, 67.5 yrs, 33.3% | 9, 64 (9) yrs, 33.3% | 9, 71 (5) yrs, 33.3% | 1.95 | parallel RCT | Immersive technology for cognitive-motor training | VR | Technology-based rehabilitation therapy | Physical rehabilitation | Supervised | Medical institution | 4w (NA) | Active control | None |
| Li, 2022 | China | Upper-middle Income | 46, 64.87 yrs, 50.0% | 23, 64.090 (11.000) yrs, 47.8% | 23, 65.650 (8.716) yrs, 0.2% | 2.61 | parallel RCT | Music-based movement therapy (MMT) | Music player | Technology-based rehabilitation therapy | Physical rehabilitation | Supervised | Medical institution | 4w (NA) | Active control | None |
| Liao, 2015 | China | Upper-middle Income | 24, 66.2 yrs, 50.0% | 12, 67.3 (7.1) yrs, 50.0% | 12, 65.1 (6.7) yrs, 0.5% | 2 | parallel RCT | Virtual reality (VR)-based training | VR | Technology-based rehabilitation therapy | Physical rehabilitation | Supervised | Medical institution | 6w (4w) | Active control | Motor symptoms |
| Maas, 2024 | Netherlands | High income | 214, 68.14, 27.10% | 109, 67.4 (8.5), 29.4% | 105, 68.9 (8.5), 24.8% | 2.19 | parallel RCT | Remotely delivered speech therapy | Smartphone and tablet or computer | Online classes and technology-based rehabilitation therapy | Physical rehabilitation | Supervised | Home | 8w (NA) | Active control | Psychiatric symptoms and Quality of life |
| Maggio, 2018 | Italy | High income | 20, 69.4 yrs, 50.0% | 10, 69.9 (6.3) yrs, 40.0% | 10, 68.9 (10.05) yrs, 0.6% | 2.2 | parallel RCT | Virtual reality training with BTS Nirvana (BTS-N) system | VR | Technology-based rehabilitation therapy | Cognitive training | Supervised | Medical institution | 8w (NA) | Active control | Cognition function |
| Maggio, 2024 | Italy | High income | 22, 62.93, 22.73% | 12, 59.7(9.7), 33.3% | 10, 66.8(6.5), 10.0% | NR | parallel RCT | Cognitive apps | Smartphone | Technology-based rehabilitation therapy | Cognitive training | Unsupervised | Home | 6w (12w) | Active control | Cognition function, Psychiatric symptoms |
| Maggio, 2025 | Italy | High income | 20, 63.7 yrs, 40% | 10, 63.6 (2.6) yrs, 40% | 10, 63.8 (2.0) yrs, 40% | 2.2 | parallel RCT | cognitive tele-rehabilitation plus virtual reality | VR | Technology-based rehabilitation therapy | Cognitive training | Remotely Supervised (Real-Time) | Home | 8w (NR) | Active control | Cognition function, Quality of life |
| Manor, 2013 | Israel | High income | 42, 68.76 yrs, 57.1% | 21, 67.66 (8.26) yrs, NR | 21, 69.86 (9.7) yrs, NR | 2.2 | parallel RCT | Video-assisted swallowing therapy | Video | Digital databases | Physical rehabilitation | Supervised | Medical institution | 4w (4w) | Active control | Motor symptoms |
| Maranesi, 2022 | Italy | High income | 30, 74 yrs, 50.0% | 16, 75.5 (5.4) yrs, 62.5% | 14, 72.7 (6.3) yrs, 35.8% | 2.14 | parallel RCT | Non-immersive virtual reality exergames | VR | Technology-based rehabilitation therapy | Physical rehabilitation | Supervised | Medical institution | 5w (NA) | Active control | Motor symptoms, Psychiatric symptoms, Quality of life |
| McGibbon, 2024 | Canada | High income | 27, 69.2(6.7) yrs, 59.3% | 13, 67.6(5.9), 69.2% | 14, 70.7(7.3), 50.0% | 1.72 | parallel RCT | Keeogo Rehab™ exoskeleton | Robotic exoskeleton | Technology-based rehabilitation therapy | Physical rehabilitation | Supervised | Medical institution | 8w (NA) | Active control | Motor symptoms, Cognition function |
| Meng-Che, 2016 | China | Upper-middle Income | 20, 68.2 yrs, 20.0% | 10, 67.5 (9.96) yrs, 10.0% | 10, 68.8 (9.67) yrs, 0.3% | 1.5 | parallel RCT | Balance-based exergaming intervention using the Kinect sensor | VR | Technology-based rehabilitation therapy | Physical rehabilitation | Supervised | Medical institution | 8w (NA) | Active control | Motor symptoms |
| Mirelman, 2016 | Israel | High income | 282, 73.8 yrs, 35.5% | 146, 74.2 (6.9) yrs, 33.0% | 136, 73.3 (6.4) yrs, 0.4% | NR | parallel RCT | Intervention combining treadmill training with non-immersive virtual reality (VR) | VR | Technology-based rehabilitation therapy | Physical rehabilitation | Supervised | Medical institution | 6w (24w) | Active control | None |
| Nieuwboer, 2007 | Belgium | High income | 153, 67.5 yrs, 84.4% | 76, 67.0 (7.9) yrs, 36.8% | 77, 68.1 (7.9) yrs, 48.0% | 2.75 | crossover RCT | Home‐based cueing programme | Computer/  Intelligent device | Technology-based rehabilitation therapy | Physical rehabilitation | Supervised | Home | 3w (NA) | Passive control | Motor symptoms, Quality of life |
| Nuvolini, 2025 | Brazil | Upper-middle Income | 38, 66.0 yrs, 13.2% | 19, 62.7 (6.8) yrs, 21.0% | 19, 69.2 (7.8), 5.3% | 2.08 | parallel RCT | training based on Kinect exergames | VR | Technology-based rehabilitation therapy | Physical rehabilitation | Supervised | Medical institution | 7w (4w) | Active control | Motor symptoms, Cognition function |
| Ophey, 2020 | Germany | High income | 75, 63.98 yrs, 46.7% | 37, 64.09 (8.56) yrs, 48.6% | 38, 63.88 (8.39) yrs, 44.7% | 2.1 | parallel RCT | Computerized working memory training (WMT) | Computer | Technology-based rehabilitation therapy | Cognitive training | Unsupervised | Home | 5w (12w) | Passive control | Motor symptoms, Cognition function |
| Özden, 2021 | Italy | High income | 51, 71 yrs, 31.4% | 25, 72 (7) yrs, 28.0% | 26, 70 (10) yrs, 34.6% | NR | parallel RCT | Virtual reality (VR) rehabilitation programme | VR | Technology-based rehabilitation therapy | Physical rehabilitation | Supervised | Medical institution | 6w (NA) | Active control | Motor symptoms, Psychiatric symptoms |
| París, 2011 | Spain | High income | 28, 65.04 yrs, 50.0% | 16, 64.75 (9.19) yrs, 56.2% | 12, 65.42 (9.60) yrs, 41.7% | 2.32 | parallel RCT | Cognitive training using multimedia software | Computer | Technology-based rehabilitation therapy | Cognitive training | Supervised | Home | 4w (NA) | Active control | Cognition function, Psychiatric symptoms, Quality of life |
| Pastana Ramos, 2023 | Brazil | Upper-middle Income | 19, 59.82 yrs, 47.4% | 8, 58.6 (9.3) yrs, 50.0% | 11, 60.7 (19.4) yrs, 45.5% | 1.89 | parallel RCT | Telerehabilitation program | Smartphone/tablet/computer | Online classes | Physical rehabilitation | Remotely Supervised (Real-Time) | Home | 4w (4w) | Active control | Motor symptoms, Quality of life |
| Patel, 2017 | America | High income | 28, 63.9 yrs, 42.9% | 14, 63.1 (6.8) yrs, 21.4% | 14, 64.7 (9.5) yrs, 64.3% | NR | parallel RCT | Computerized CBT for insomnia (CCBT-I) | Computer | Technology-based rehabilitation therapy | Cognitive training | Unsupervised | NR | 6w (NA) | Active control | Motor symptoms, Psychiatric symptoms, Overall non-motor symptoms, Quality of life |
| Peacock, 2021 | Canada | High income | 25, 70 yrs, 52.0% | 13, 70 (6) yrs, 46.0% | 12, 71 (7) yrs, 0.6% | NR | parallel RCT | Wearable systems and telehealth | Computer and wearable sensor | Online classes | Care aid | Remotely Supervised (Asynchronous) | Medical institution | 6w (NA) | Active control | Quality of life |
| Picelli, 2012 | Italy | High income | 34, 68.3 yrs, 41.2% | 17, NR, NR | 17, NR, NR | 3.45 | parallel RCT | Robot-assisted gait training | Robot-assisted | Technology-based rehabilitation therapy | Physical rehabilitation | Supervised | Medical institution | 4w (4w) | Active control | Motor symptoms |
| Picelli, 2013 | Italy | High income | 40, 68.65 yrs, 62.5% | 20, 68.50 (10.10) yrs, 55.0% | 20, 68.80 (7.72) yrs, 0.7% | 3 | parallel RCT | Robot-assisted gait training | Robot-assisted | Technology-based rehabilitation therapy | Physical rehabilitation | Supervised | Medical institution | 4w (12w) | Active control | Motor symptoms |
| Picelli, 2015 | Italy | High income | 66, 68.9 yrs, 27.3% | 33, 68.2 (9.2) yrs, 21.2% | 33, 69.7 (7.2) yrs, 33.3% | NR | parallel RCT | Robot-assisted gait training | Robot-assisted | Technology-based rehabilitation therapy | Physical rehabilitation | Supervised | Medical institution | 4w (4w) | Active control | Motor symptoms |
| Piers, 2023 | America | High income | 12, 61.3 yrs, 66.7% | 6, 58.5 (8.1) yrs, 66.7% | 6, 64.1 (5.9) yrs, 66.7% | NR | parallel RCT | Telehealth transdiagnostic cognitive behavioral therapy (CBT) | Computer | Online classes | Cognitive training | Remotely Supervised (Real-Time) | NR | 12w (NA) | Passive control | Psychiatric symptoms |
| Pinto, 2025 | United Kingdom | High income | 44, 66.2 yrs, 31.8% | 25, 51.0 (7.6) yrs, 32.0% | 19, 63.8 (8.0) yrs, 31.6% | NR | parallel RCT | A web application (PACT app) based on Acceptance and Commitment Therapy | smartphones and tablets | Online classes | Care aid | Unsupervised | Home | 4w (NR) | Active control | Psychiatric symptoms, Quality of life |
| Pompeu, 2012 | Brazil | Upper-middle Income | 32, 67.4 yrs, 46.9% | 16, 68.6 (8.0) yrs, NR | 16, 66.2 (8.3) yrs, NR | NR | parallel RCT | Nintendo Wii™-based motor cognitive training | VR | Technology-based rehabilitation therapy | Physical rehabilitation | Supervised | Medical institution | 7w (8w) | Active control | Motor symptoms, Cognition function |
| Qayyum, 2022 | Pakistan | Lower-middle Income | NR | NR | NR | NR | parallel RCT | Virtual based games | VR | Technology-based rehabilitation therapy | Physical rehabilitation | NR | NR | 8w (NA) | Active control | Motor symptoms |
| Raciti, 2022 | Italy | High income | 24, 64.6 yrs, NR | 15, 65.7 (7) yrs, NR | 9, 62.7 (10.1) yrs, NR | 2.37 | parallel RCT | Semi-autonomous exercises using an upper extremity exoskeleton | Robot-assisted | Technology-based rehabilitation therapy | Physical rehabilitation | Supervised | Medical institution | 8w (NA) | Active control | Motor symptoms |
| Raglio, 2023 | Italy | High income | 19, 72.42, 26.32% | 10, 74.6(5.6), 20% | 9, 70(6.3), 33.3% | NR | parallel RCT | Gait training program with movement sonification | headphone and computer | Technology-based rehabilitation therapy | Physical rehabilitation | NR | Medical institution | 7w (4w) | Active control | Motor symptoms, Quality of life |
| Ribas, 2017 | Brazil | Upper-middle Income | 20, 61 yrs, 60.0% | 10, 61.70 (6.83) yrs, 60.0% | 10, 60.20 (11.29) yrs, 0.6% | 1.47 | parallel RCT | Exergaming | VR | Technology-based rehabilitation therapy | Physical rehabilitation | Supervised | Medical institution | 12w (8w) | Active control | Motor symptoms |
| Sale, 2013 | Italy | High income | 20, 69.34 yrs, 45.0% | 10, 70.27 (9.81) yrs, 40.0% | 10, 68.42 (9.41) yrs, 50.0% | 3 | parallel RCT | End-effector robot training | Robot-assisted | Technology-based rehabilitation therapy | Physical rehabilitation | Supervised | Medical institution | 4w (NA) | Active control | Motor symptoms |
| Santos, 2019 | Brazil | Upper-middle Income | 27, 63.1 yrs, 18.5% | 13, 61.7 (7.3) yrs, 15.4% | 14, 64.5 (9.8) yrs, 21.4% | 1.4 | parallel RCT | Combination of Nintendo Wii with Conventional Exercises (CE) | VR | Technology-based rehabilitation therapy | Physical rehabilitation | Supervised | Medical institution | 8w (NA) | Active control | Motor symptoms, Quality of life |
| Sekimoto, 2019 | Japan | High income | 20, 53.5 yrs, 30.0% | 10, NR, 30.0% | 10, NR, 0.3% | 2 | crossover RCT | Video-based telemedicine system delivered via a tablet | Tablet | Online classes | Care aid | Remotely Supervised (Real-Time) | Home | 24w (NA) | Passive control | None |
| So, 2023 | Korea | High income | 50, 59.52 yrs, 52.0% | 25, 59.16 (6.75) yrs, 56.0% | 25, 59.88 (7.57) yrs, 58.0% | 2.5 | parallel RCT | Home-based self-management intervention | Telephone smartphone and wearable device | Digital databases, online classes and technology-based rehabilitation therapy | Care aid and physical rehabilitation | Unsupervised | Home | 16w (NA) | Active control | Overall non-motor symptoms, Quality of life |
| Song, 2018 | Australia | High income | 60, 66.55 yrs, 60.0% | 31, 68 (7) yrs, 52.0% | 29, 65 (7) yrs, 0.7% | NR | parallel RCT | Home-based step training using videogame technology | Computer | Technology-based rehabilitation therapy | Physical rehabilitation | Unsupervised | Home | 12w (NA) | Passive control | Motor symptoms, Cognition function |
| Spina, 2021 | Italy | High income | 22, 67.635 yrs, 40.9% | 11, 68 (6.9) yrs, 45.5% | 11, 67.27 (4.85) yrs, 36.4% | 1.68 | parallel RCT | Tailored robotic platform training | Robot-assisted | Technology-based rehabilitation therapy | Physical rehabilitation | Supervised | Medical institution | 4w (4w) | Active control | Motor symptoms, Quality of life |
| Svaerke, 2022 | Denmark | High income | 20, 65.15 yrs, 62.5% | 10, 65.8 (9.9) yrs, 62.5% | 10, 64.5 (11.0) yrs, 62.5% | 1.85 | parallel RCT | Computer-based cognitive rehabilitation (CBCR) | Computer | Online classes | Cognitive training | Unsupervised | Home | 8w (NA) | Passive control | Cognition function, Psychiatric symptoms, Quality of life |
| Tagliente, 2025 | Italy | High income | 25, 69.32 yrs, 28% | 15, 67.53 (7.26) yrs, NR | 10, 72 (6.58) yrs, NR | 2.52 | crossover RCT | Home-Based Computerized Cognitive Training (HB-CCT) | Computer | Technology-based rehabilitation therapy | Cognitive training | Remotely Supervised (Real-Time) | Home | 5w (NR) | Passive control | None |
| Tayyebi, 2025 | Iran | Upper-middle Income | 90, NR, 46.7% | 45, NR, 46.7% | 45, NR, 46.7% | NR | parallel RCT | Virtual reality-based cognitive behavioral group therapy (VR-CBGT) | VR | Technology-based rehabilitation therapy | Cognitive training | Supervised | Medical institution | 12w (NR) | Passive control | Psychiatric symptoms |
| Theodoros, 2015 | Australia | High income | 31, 71.77 yrs, 45.2% | 15, 71.1 (7.809) yrs, 16.7% | 16, 72.375 (10.032) yrs, 33.3% | 1.81 | parallel RCT | Intensive speech treatment delivered via telerehabilitation to the home | Computer | Online classes | Physical rehabilitation | Remotely Supervised (Real-Time) | Medical institution | NA | Active control | Quality of life |
| van Balkom, 2022 | Netherlands | High income | 136, 62.9 yrs, 9.7% | 68, 62.9 (8.1) yrs, 49.0% | 68, 62.9 (7.0) yrs, 0.3% | 2.18 | parallel RCT | Computerized cognitive training | Computer | Technology-based rehabilitation therapy | Cognitive training | Unsupervised | Home | 8w (24w) | Active control | Cognition function |
| van de Weijer, 2020 | Netherlands | High income | 41, 64.32 yrs, NR | 20, 64.65 (7.40) yrs, NR | 21, 64.01 (7.41) yrs, NR | 1.88 | parallel RCT | Home-based gamified cognitive training | NR | Technology-based rehabilitation therapy | Cognitive training | Unsupervised | Home | 12w (12w) | Passive control | Cognition function |
| van den Heuvel, 2014 | Netherlands | High income | 33, 67.51 yrs, 39.4% | 17, 66.3 (6.39) yrs, 29.4% | 16, 68.8 (9.68) yrs, 50.0% | 2.5 | parallel RCT | Balance training program using augmented visual feedback | Wearable sensors | Technology-based rehabilitation therapy | Physical rehabilitation | Supervised | Medical institution | 6w (NA) | Active control | Motor symptoms, Psychiatric symptoms, Quality of life |
| Wilkinson, 2016 | America | High income | 36, 69.05 yrs, NR | 18, 67.2 (9.8) yrs, NR | 18, 70.9 (8.4) yrs, NR | 2.4 | parallel RCT | Telehealth | Telehealth specialty carts and webcams | Digital databases | Care aid | Remotely Supervised (Real-Time) | Home | 48w (NA) | Active control | Motor symptoms, Psychiatric symptoms |
| Yang, 2016 | China | Upper-middle Income | 23, 73.95 yrs, 39.1% | 11, 72.5 (8.4) yrs, 36.4% | 12, 75.4 (6.3) yrs, 41.7% | 3 | parallel RCT | Home-based virtual reality balance training | VR | Technology-based rehabilitation therapy | Physical rehabilitation | Supervised | Home | 6w (2w) | Active control | Motor symptoms, Quality of life |
| Yen, 2011 | China | Upper-middle Income | 28, 70.25 yrs, 16.7% | 14, 70.4 (6.5) yrs, 16.7% | 14, 70.1 (6.9) yrs, 16.7% | 2.5 | parallel RCT | Virtual reality-augmented balance training | VR | Technology-based rehabilitation therapy | Physical rehabilitation | Supervised | Medical institution | 6w (4w) | Active control | Motor symptoms |
| Yuan, 2020 | China | Upper-middle Income | 24, 67.15 yrs, 54.2% | 12, 67.8 (5.5) yrs, 3.3% | 12, 66.5 (8.8) yrs, 25.0% | 2.04 | crossover RCT | Customized interactive video game-based (IVGB) training | Computer | Technology-based rehabilitation therapy | Physical rehabilitation | Supervised | Medical institution | 6w (NA) | Passive control | Motor symptoms, Psychiatric symptoms |
| Zoetewei, 2024 | Belgium | High income | 63, 68.19, 30.16% | 32, 67.7 (7.97), 34.38% | 31, 68.7 (7.31), 25.81% | NR | parallel RCT | On-Demand Cueing for Freezing of Gait | Smartphone and earphone | Technology-based rehabilitation therapy | Physical rehabilitation | Unsupervised | Home | 4w (NA) | Active control | Motor symptoms |

^a^IG: Intervention Group

^b^M: Mean

^c^SD: Standard Deviation

^d^CG: Control Group

^e^yrs: years

^f^VR: Virtual Reality

^g^NR: Not Reported
