# Supplementary material for: The Effects of Digital Health Interventions on Motor Symptoms, Nonmotor Symptoms, and Quality of Life in Patients With Parkinson Disease: Systematic Review and Meta-Analysis of Randomized Controlled Trials
Source: J Med Internet Res. 2026 Mar 12;28:e79935. doi: 10.2196/79935 (PMC13147926; doi:10.2196/79935)
Supplement: Multimedia Appendix 7 [file jmir_v28i1e79935_app7.docx]

**Multimedia Appendix 6. Risk of bias for included effect estimates based on the Cochrane risk-of-bias tool for randomized trials (RoB2).**

|  |  | **Domain** | | | | | |
| --- | --- | --- | --- | --- | --- | --- | --- |
| **Study ID** | **Outcome** | **Randomization process** | **Deviations from intended interventions** | **Missing outcome data** | **Measurement of the outcome** | **Selection of the reported result** | **Overall Bias** |
| Albert 2023 | motor symptoms | Low | Low | Low | Low | Low | Low |
| Allen 2017 | motor symptoms | Low | Some concerns | Some concerns | Low | Low | Some concerns |
| Allen 2017 | cognitive performance | Low | Some concerns | Some concerns | Low | Low | Some concerns |
| Allen 2017 | quality of life | Low | Some concerns | Some concerns | Low | Low | Some concerns |
| Bartolo, 2024 | motor symptoms | Low | Low | Low | Low | Low | Low |
| Beck 2017 | motor symptoms | Low | Some concerns | Low | Low | Low | Some concerns |
| Beck 2017 | cognitive performance | Low | Some concerns | Low | Low | Low | Some concerns |
| Beck 2017 | psychiatric symptoms | Low | Some concerns | Low | Low | Low | Some concerns |
| Beck 2017 | overall non-motor symptoms | Low | Some concerns | Low | Low | Low | Some concerns |
| Beck 2017 | quality of life | Low | Some concerns | Low | Low | Low | Some concerns |
| Bernini 2019 | motor symptoms | Low | High | Some concerns | Low | Some concerns | High |
| Bernini 2019 | cognitive performance | Low | High | Some concerns | Low | Some concerns | High |
| Bernini 2019 | psychiatric symptoms | Low | High | Some concerns | Low | Some concerns | High |
| Bernini 2021 | cognitive performance | Low | Low | Low | Low | Low | Low |
| Bogosian 2022 | psychiatric symptoms | Low | Some concerns | High | Low | Low | High |
| Calabrò 2019 | motor symptoms | Some concerns | Low | Low | Low | Some concerns | Some concerns |
| Capecci 2019 | motor symptoms | Low | Some concerns | Low | Low | Some concerns | Some concerns |
| Capecci 2019 | quality of life | Low | Some concerns | Low | Low | Some concerns | Some concerns |
| Carda 2012 | motor symptoms | Low | Some concerns | Low | Low | Low | Some concerns |
| Carda 2012 | psychiatric symptoms | Low | Some concerns | Low | Low | Low | Some concerns |
| Carpinella 2016 | quality of life | Some concerns | High | Low | Low | Some concerns | High |
| Carpinella 2016 | motor symptoms | Some concerns | High | Low | Low | Some concerns | High |
| Çetin, 2024 | motor symptoms | Low | Some concerns | Low | Low | Low | Some concerns |
| Çetin, 2024 | cognitive performance | Low | Some concerns | Low | Low | Low | Some concerns |
| Çetin, 2024 | quality of life | Low | Some concerns | Low | Low | Low | Some concerns |
| Da Silva 2022 | motor symptoms | Low | Low | Some concerns | Low | Low | Some concerns |
| Da Silva 2022 | quality of life | Low | Low | Some concerns | Low | Low | Some concerns |
| Das, 2024 | motor symptoms | Low | Some concerns | Low | Low | Some concerns | Some concerns |
| Das, 2024 | cognitive performance | Low | Some concerns | Low | Low | Some concerns | Some concerns |
| Das, 2024 | quality of life | Low | Some concerns | Low | Low | Some concerns | Some concerns |
| De Luca 2019 | psychiatric symptoms | Low | Low | Low | Low | Some concerns | Some concerns |
| De, 2025 | motor symptoms | Low | Low | Low | Low | Low | Some concerns |
| Del Pino 2023 | motor symptoms | Some concerns | Some concerns | Low | Low | Some concerns | Some concerns |
| Del Pino 2023 | cognitive performance | Some concerns | Some concerns | Low | Low | Some concerns | Some concerns |
| Del Pino 2023 | overall non-motor symptoms | Some concerns | Some concerns | Low | Low | Some concerns | Some concerns |
| Del Pino 2023 | quality of life | Some concerns | Some concerns | Low | Low | Some concerns | Some concerns |
| Dhamija, 2025 | motor symptoms | Low | Low | Low | Low | Some concerns | Some concerns |
| Dhamija, 2025 | overall non-motor symptoms | Low | Low | Low | Low | Some concerns | Some concerns |
| Dhamija, 2025 | quality of life | Low | Low | Low | Low | Some concerns | Some concerns |
| Dobkin 2020 | psychiatric symptoms | Low | Some concerns | Low | Low | Low | Some concerns |
| Dobkin 2021 | psychiatric symptoms | Low | Some concerns | High | Low | High | High |
| Dorsey 2010 | motor symptoms | Some concerns | Low | Low | High | Low | High |
| Dorsey 2010 | cognitive performance | Some concerns | Low | Low | High | Low | High |
| Dorsey 2010 | psychiatric symptoms | Some concerns | Low | Low | High | Low | High |
| Dorsey 2010 | quality of life | Some concerns | Low | Low | High | Low | High |
| Dorsey 2013 | motor symptoms | Some concerns | Low | Low | High | Low | High |
| Dorsey 2013 | quality of life | Some concerns | Low | Low | High | Low | High |
| Eldemir 2023 | quality of life | Low | Low | Low | Low | Low | Low |
| Eldemir 2023 | motor symptoms | Low | Low | Low | Low | Low | Low |
| Ellis 2019 | quality of life | Low | Low | Some concerns | Low | High | High |
| Ellis 2019 | motor symptoms | Low | Low | Some concerns | Low | High | High |
| Fellman 2022 | psychiatric symptoms | Some concerns | Some concerns | Low | Low | Some concerns | Some concerns |
| Fellman 2022 | cognitive performance | Some concerns | Some concerns | Low | Low | Some concerns | Some concerns |
| Feng 2019 | motor symptoms | Some concerns | Low | Low | Low | Some concerns | Some concerns |
| Ferraz 2018 | motor symptoms | Low | Some concerns | Low | Low | High | High |
| Ferraz 2018 | quality of life | Low | Some concerns | Low | Low | High | High |
| Ferraz 2018 | psychiatric symptoms | Low | Some concerns | Low | Low | High | High |
| Flynn 2021 | motor symptoms | Some concerns | Some concerns | Low | Low | Low | Some concerns |
| Furnari 2017 | psychiatric symptoms | Low | Low | Low | Low | High | High |
| Furnari 2017 | motor symptoms | Low | Low | Low | Low | High | High |
| Galli 2016 | motor symptoms | Low | Low | Low | Low | Some concerns | Some concerns |
| Gandolfi 2017 | quality of life | Low | Low | Low | High | Some concerns | High |
| Gandolfi 2017 | motor symptoms | Low | Low | Low | High | Some concerns | High |
| Giehl 2020 | cognitive performance | Low | Some concerns | Low | Low | High | High |
| Ginis 2016 | motor symptoms | Low | Low | Low | Low | Some concerns | Some concerns |
| Ginis 2016 | cognitive performance | Low | Low | Low | Low | Some concerns | Some concerns |
| Ginis 2016 | psychiatric symptoms | Low | Low | Low | Low | Some concerns | Some concerns |
| Glicia Pedreira 2013 | quality of life | Low | High | Some concerns | Low | Some concerns | High |
| Goffredo 2023 | motor symptoms | Some concerns | Low | Low | Low | Low | Some concerns |
| Gryfe 2022 | motor symptoms | Low | Low | Low | Low | Low | Low |
| Gryfe 2022 | quality of life | Low | Low | Low | Low | Low | Low |
| Gryfe 2022 | psychiatric symptoms | Low | Low | Low | Low | Low | Low |
| Gryfe 2022 | cognitive performance | Low | Low | Low | Low | Low | Low |
| Gulcan 2023 | motor symptoms | Some concerns | Some concerns | Low | Low | Some concerns | Some concerns |
| Hajebrahimi 2022 | psychiatric symptoms | Low | Some concerns | Some concerns | Low | Some concerns | Some concerns |
| Hajebrahimi 2022 | cognitive performance | Low | Some concerns | Some concerns | Low | Some concerns | Some concerns |
| Hajebrahimi 2022 | motor symptoms | Low | Some concerns | Some concerns | Low | Some concerns | Some concerns |
| Hajebrahimi 2022 | quality of life | Low | Some concerns | Some concerns | Low | Some concerns | Some concerns |
| Han 2023 | motor symptoms | Low | Some concerns | Low | Low | Some concerns | Some concerns |
| Harpham, 2025 | motor symptoms | Low | Some concerns | Low | Low | Low | Some concerns |
| Hashemi 2022 | motor symptoms | Low | Low | Low | Low | Low | Low |
| Heldman 2017 | motor symptoms | Some concerns | Some concerns | Low | Low | Some concerns | High |
| Heldman 2017 | overall non-motor symptoms | Some concerns | Some concerns | Low | Low | Some concerns | High |
| Heldman 2017 | quality of life | Some concerns | Some concerns | Low | Low | Some concerns | High |
| Isaacson 2019 | quality of life | Some concerns | Some concerns | Low | Low | Some concerns | Some concerns |
| Isaacson 2019 | motor symptoms | Some concerns | Some concerns | Low | Low | Some concerns | Some concerns |
| Jäggi 2023 | motor symptoms | Low | Some concerns | Low | Some concerns | Low | Some concerns |
| Jäggi 2023 | cognitive performance | Low | Some concerns | Low | Some concerns | Low | Some concerns |
| Johnson, 2024 | motor symptoms | High | Some concerns | Low | Some concerns | Low | High |
| Johnson, 2024 | quality of life | High | Some concerns | Low | Some concerns | Low | High |
| Jong-Hoon 2020 | motor symptoms | Some concerns | Low | Low | Low | Some concerns | Some concerns |
| Kashif 2022 | motor symptoms | Some concerns | Some concerns | Low | Low | Low | Some concerns |
| Kashif, 2024 | motor symptoms | Low | Some concerns | Low | Low | Low | Some concerns |
| Kawashima 2022 | motor symptoms | Some concerns | Some concerns | Low | Some concerns | Low | Some concerns |
| Kawashima 2022 | quality of life | Some concerns | Some concerns | Low | Some concerns | Low | Some concerns |
| Khalil 2017 | motor symptoms | Low | Some concerns | Low | Low | Some concerns | Some concerns |
| Kim 2022 | motor symptoms | Some concerns | Low | Low | Low | Low | Some concerns |
| Kim 2022 | overall non-motor symptoms | Some concerns | Low | Low | Low | Low | Some concerns |
| Kluger 2023 | psychiatric symptoms | Some concerns | Low | Low | Low | Low | Some concerns |
| Kluger 2023 | quality of life | Some concerns | Low | Low | Low | Low | Some concerns |
| Kraepelien 2020 | quality of life | Low | Low | Low | Low | Low | Low |
| Kraepelien 2020 | psychiatric symptoms | Low | Low | Low | Low | Low | Low |
| Lai 2020 | motor symptoms | Some concerns | High | Some concerns | Low | Some concerns | High |
| Lakshminarayana 2017 | psychiatric symptoms | Low | Some concerns | Some concerns | Low | Some concerns | Some concerns |
| Lakshminarayana 2017 | overall non-motor symptoms | Low | Some concerns | Some concerns | Low | Some concerns | Some concerns |
| Lakshminarayana 2017 | quality of life | Low | Some concerns | Some concerns | Low | Some concerns | Some concerns |
| Liao 2015 | motor symptoms | Low | Low | Low | Low | Some concerns | Some concerns |
| Maas, 2024 | quality of life | Low | Some concerns | Low | Low | Low | Some concerns |
| Maas, 2024 | psychiatric symptoms | Low | Some concerns | Low | Low | Low | Some concerns |
| Maggio 2018 | cognitive performance | Some concerns | Low | Low | Low | Some concerns | Some concerns |
| Maggio, 2024 | psychiatric symptoms | Low | Some concerns | Some concerns | Low | Some concerns | Some concerns |
| Maggio, 2024 | cognitive performance | Low | Some concerns | Some concerns | Low | Some concerns | Some concerns |
| Maggio, 2025 | cognitive performance | Low | Low | Low | Low | Low | Low |
| Maggio, 2025 | quality of life | Low | Low | Low | Low | Low | Low |
| Manor 2013 | motor symptoms | Some concerns | Low | Low | Low | Some concerns | Some concerns |
| Maranesi 2022 | motor symptoms | Low | Some concerns | Low | Low | Some concerns | Some concerns |
| Maranesi 2022 | psychiatric symptoms | Low | Some concerns | Low | Low | Some concerns | Some concerns |
| Maranesi 2022 | quality of life | Low | Some concerns | Low | Low | Some concerns | Some concerns |
| McGibbon, 2024 | cognitive performance | Some concerns | Some concerns | Some concerns | Low | Low | Some concerns |
| McGibbon, 2024 | motor symptoms | Some concerns | Some concerns | Some concerns | Low | Low | Some concerns |
| Meng-Che 2016 | motor symptoms | Low | Some concerns | Low | Some concerns | Low | Some concerns |
| Nieuwboer 2007 | motor symptoms | Low | Low | Low | Low | Some concerns | Some concerns |
| Nieuwboer 2007 | quality of life | Low | Low | Low | Low | Some concerns | Some concerns |
| Nuvolini, 2025 | motor symptoms | Low | Low | High | Low | Some concerns | High |
| Nuvolini, 2025 | cognitive performance | Low | Low | High | Low | Some concerns | High |
| Ophey2020 | motor symptoms | Low | Low | Low | Low | Low | Low |
| Ophey2020 | cognitive performance | Low | Low | Low | Low | Low | Low |
| Özden 2021 | motor symptoms | Low | Low | Low | Low | Low | Low |
| Özden 2021 | psychiatric symptoms | Low | Low | Low | Low | Low | Low |
| París 2011 | psychiatric symptoms | Some concerns | Some concerns | Low | Low | Some concerns | Some concerns |
| París 2011 | quality of life | Some concerns | Some concerns | Low | Low | Some concerns | Some concerns |
| París 2011 | cognitive performance | Some concerns | Some concerns | Low | Low | Some concerns | Some concerns |
| Pastana Ramos 2023 | quality of life | Low | Low | Low | Low | High | High |
| Pastana Ramos 2023 | motor symptoms | Low | Low | Low | Low | High | High |
| Patel 2017 | motor symptoms | Low | High | High | Low | Some concerns | High |
| Patel 2017 | quality of life | Low | High | High | Low | Some concerns | High |
| Patel 2017 | overall non-motor symptoms | Low | High | High | Low | Some concerns | High |
| Patel 2017 | psychiatric symptoms | Low | High | High | Low | Some concerns | High |
| Peacock 2021 | quality of life | Low | Some concerns | High | Low | Some concerns | High |
| Picelli 2012 | motor symptoms | Low | Some concerns | Low | Low | Some concerns | Some concerns |
| Picelli 2013 | motor symptoms | Low | Low | Low | Low | Some concerns | Some concerns |
| Picelli 2015 | motor symptoms | Low | Low | Low | Low | Some concerns | Some concerns |
| Piers 2023 | psychiatric symptoms | Low | High | Low | Low | Some concerns | High |
| Pinto, 2025 | psychiatric symptoms | Low | Low | Some concerns | Low | Low | Some concerns |
| Pinto, 2025 | quality of life | Low | Low | Some concerns | Low | Low | Some concerns |
| Pompeu, 2012 | motor symptoms | Low | Low | Low | Low | Low | Low |
| Pompeu, 2012 | cognitive performance | Low | Low | Low | Low | Low | Low |
| Qayyum 2022 | motor symptoms | Low | Low | Low | Some concerns | Some concerns | Some concerns |
| Raciti 2022 | motor symptoms | Low | Some concerns | Some concerns | Low | High | High |
| Raglio, 2023 | motor symptoms | Low | Low | Some concerns | Low | Low | Some concerns |
| Raglio, 2023 | quality of life | Low | Low | Some concerns | Low | Low | Some concerns |
| Ribas 2017 | motor symptoms | Low | Low | Low | Low | Low | Low |
| Sale 2013 | motor symptoms | Low | Low | Low | Low | High | High |
| Santos 2019 | motor symptoms | Some concerns | Some concerns | Low | Low | Low | Some concerns |
| Santos 2019 | quality of life | Some concerns | Some concerns | Low | Low | Low | Some concerns |
| So 2023 | quality of life | Some concerns | Low | High | Low | Some concerns | High |
| So 2023 | overall non-motor symptoms | Some concerns | Low | High | Low | Some concerns | High |
| Song 2018 | motor symptoms | Low | Low | Low | Low | Low | Low |
| Song, 2018 | cognitive performance | Low | Low | Low | Low | Low | Low |
| Spina 2021 | quality of life | Low | Low | Low | Low | Some concerns | Some concerns |
| Spina 2021 | motor symptoms | Low | Low | Low | Low | Some concerns | Some concerns |
| Svaerke 2022 | quality of life | Low | Some concerns | Some concerns | Low | Low | Some concerns |
| Svaerke 2022 | psychiatric symptoms | Low | Some concerns | Some concerns | Low | Low | Some concerns |
| Svaerke 2022 | cognitive performance | Low | Some concerns | Some concerns | Low | Low | Some concerns |
| Tayyebi, 2025 | psychiatric symptoms | Low | Some concerns | High | Low | Low | Some concerns |
| Theodoros 2016 | quality of life | Low | Low | Low | Low | Some concerns | Some concerns |
| van Balkom 2022 | cognitive performance | Some concerns | Low | Low | Low | Some concerns | Some concerns |
| van Balkom 2022 | psychiatric symptoms | Some concerns | Low | Low | Low | Some concerns | Some concerns |
| van de Weijer 2020 | cognitive performance | Some concerns | Low | Low | Low | Some concerns | Some concerns |
| van den Heuvel 2014 | quality of life | Low | Low | Low | Low | Low | Low |
| van den Heuvel 2014 | motor symptoms | Low | Low | Low | Low | Low | Low |
| van den Heuvel 2014 | psychiatric symptoms | Low | Low | Low | Low | Low | Low |
| Wilkinson 2016 | motor symptoms | Low | Some concerns | Some concerns | Low | Some concerns | Some concerns |
| Wilkinson 2016 | psychiatric symptoms | Low | Some concerns | Some concerns | Low | Some concerns | Some concerns |
| Yang 2016 | quality of life | Some concerns | Some concerns | Low | Low | High | High |
| Yang 2016 | motor symptoms | Some concerns | Some concerns | Low | Low | High | High |
| Yen 2011 | motor symptoms | Low | Low | Low | Low | Low | Low |
| Yuan 2020 | motor symptoms | High | Low | Low | Low | Low | High |
| Yuan 2020 | psychiatric symptoms | High | Low | Low | Low | Low | High |
| Zoetewei, 2024 | motor symptoms | Low | Some concerns | Some concerns | Low | Low | Some concerns |
