# Supplementary material for: The Effects of Digital Health Interventions on Motor Symptoms, Nonmotor Symptoms, and Quality of Life in Patients With Parkinson Disease: Systematic Review and Meta-Analysis of Randomized Controlled Trials
Source: J Med Internet Res. 2026 Mar 12;28:e79935. doi: 10.2196/79935 (PMC13147926; doi:10.2196/79935)
Supplement: Multimedia Appendix 8 [file jmir_v28i1e79935_app8.docx]

**Multimedia Appendix 7. Pairwise Comparisons of Intervention Type Subgroups.**

| Outcome | Comparison | Effect Size (^a^SMD [95%^b^CI]) | P Value |
| --- | --- | --- | --- |
| Motor symptoms | Technology-based rehabilitation devices vs Online classes | -0.17 [-0.64 to 0.31] | 0.4874 |
|  | Technology-based rehabilitation devices vs Digital databases | 0.40 [-0.40 to 1.20] | 0.3311 |
|  | Online classes vs Digital databases | 0.57 [-0.31 to 1.44] | 0.2049 |
| Psychiatric symptoms | Technology-based rehabilitation devices vs Online classes | -0.06 [-0.72 to 0.60] | 0.8563 |
| Cognitive symptoms | Technology-based rehabilitation devices vs Online classes | 0.39 [0.01 to 0.77] | 0.0499 |
| Quality of life | Technology-based rehabilitation devices vs Online classes | 0.11 [-0.47 to 0.68] | 0.7147 |

^a^SMD: Standardized Mean Difference

^b^CI: Confidence Interval
