# Supplementary material for: The Effects of Digital Health Interventions on Motor Symptoms, Nonmotor Symptoms, and Quality of Life in Patients With Parkinson Disease: Systematic Review and Meta-Analysis of Randomized Controlled Trials
Source: J Med Internet Res. 2026 Mar 12;28:e79935. doi: 10.2196/79935 (PMC13147926; doi:10.2196/79935)
Supplement: Multimedia Appendix 9 [file jmir_v28i1e79935_app9.docx]

**Multimedia Appendix 8. Meta-analysis of digital health interventions' efficacy on follow-up motor symptoms, psychiatric symptoms, cognitive function, and quality of life.**


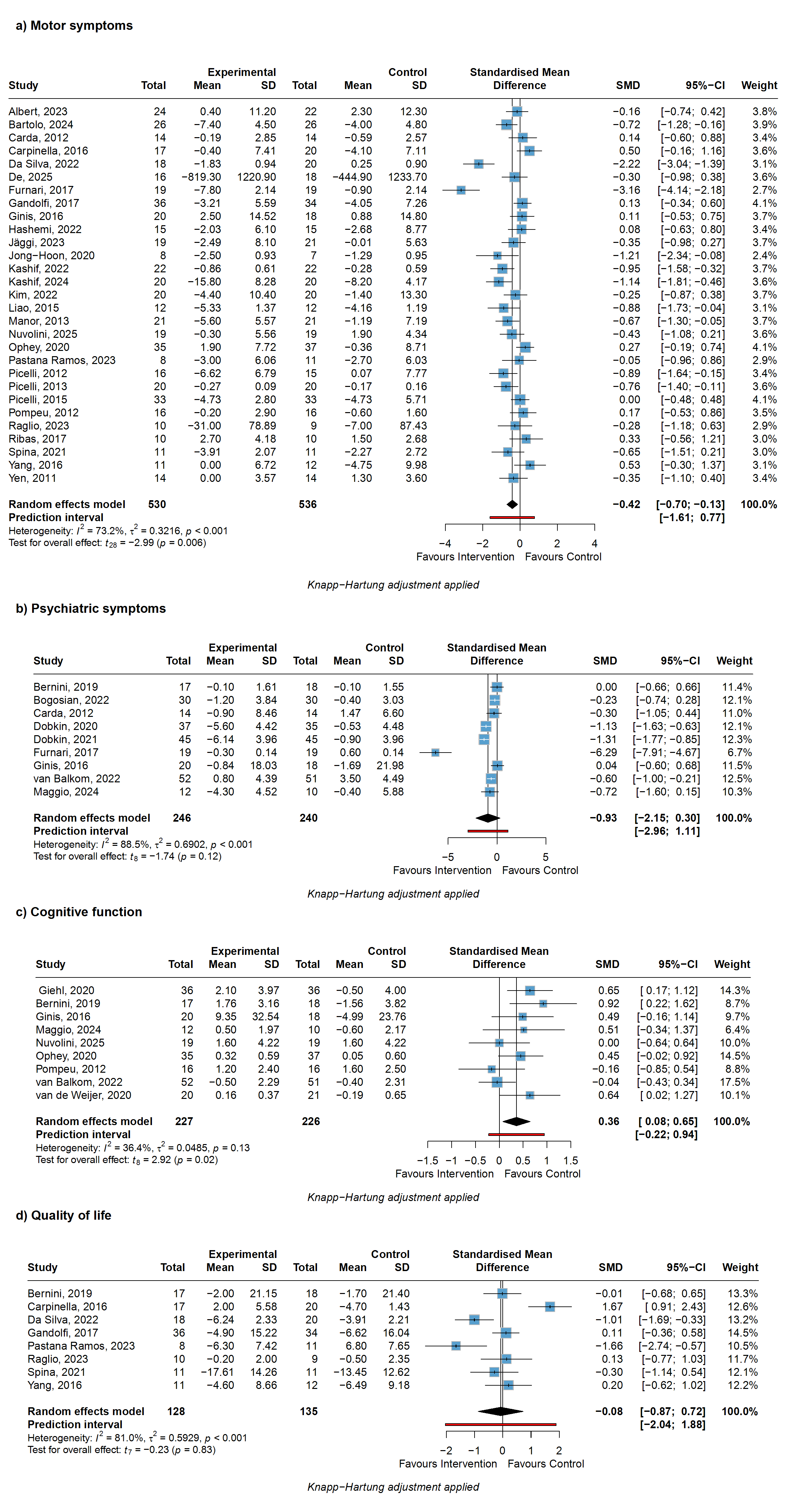


Note. Forest plots. Standardized Mean Differences (SMDs) with 95% confidence intervals (95% CI) were calculated using a random-effects model. Negative SMD values favor the experimental intervention of motor symptoms, psychiatric symptoms, and quality of life, negative SMD values favor the experimental intervention. The square shape represents the lowest accuracy in each study. The rhombus shape represents the pooled estimates of the lowest accuracy in all studies. p p-value.
