# Supplementary material for: The Effects of Digital Health Interventions on Motor Symptoms, Nonmotor Symptoms, and Quality of Life in Patients With Parkinson Disease: Systematic Review and Meta-Analysis of Randomized Controlled Trials
Source: J Med Internet Res. 2026 Mar 12;28:e79935. doi: 10.2196/79935 (PMC13147926; doi:10.2196/79935)
Supplement: Multimedia Appendix 10 [file jmir_v28i1e79935_app10.docx]

**Multimedia Appendix 9. Publication bias analyses – Funnel plots and trim and fill analyses at post-intervention assessments on motor symptoms, psychiatric symptoms, cognitive function, overall non-motor symptoms and quality of life.**


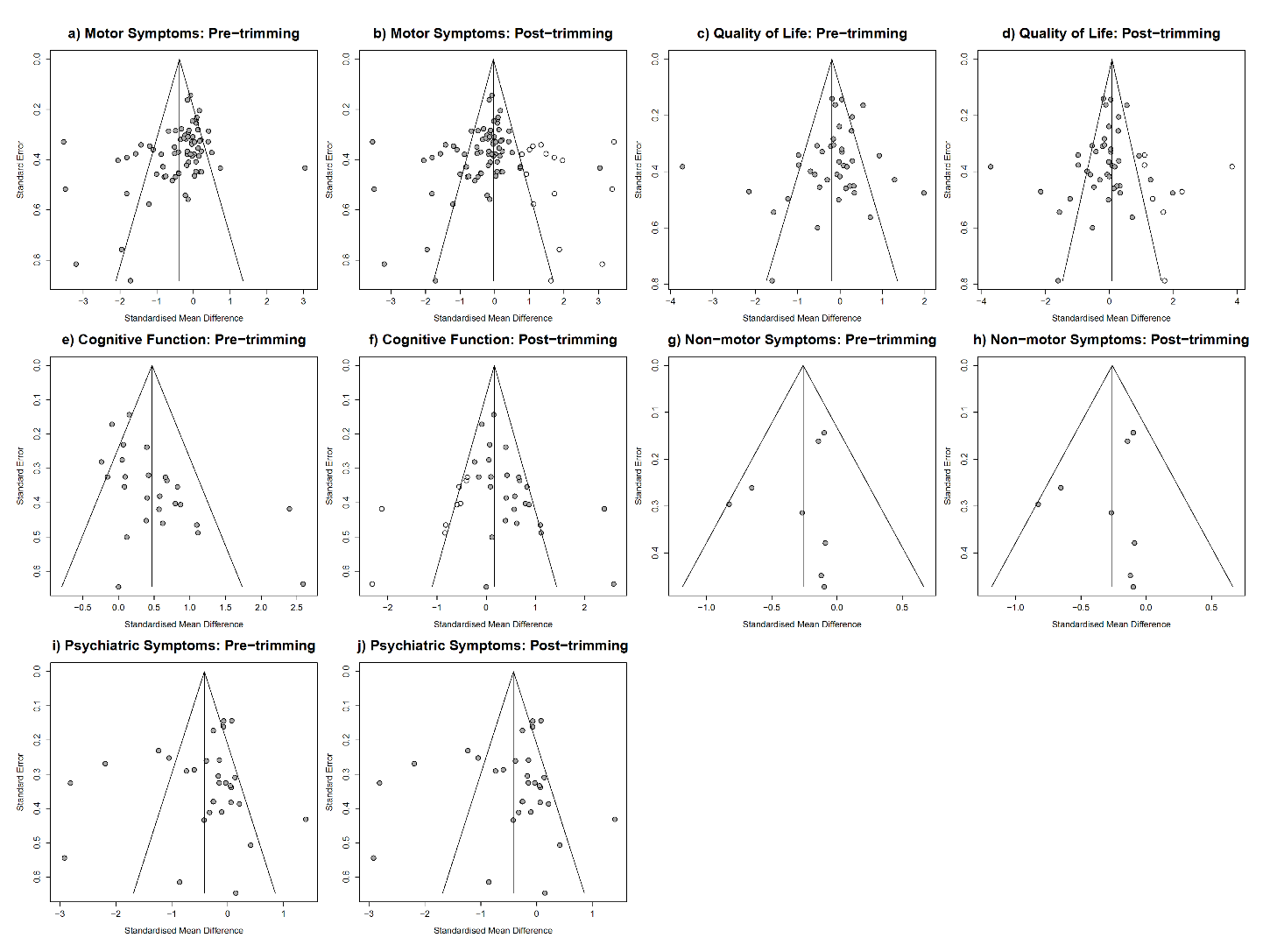


Note. Each circle represents an individual study, with the x-axis depicting the standardized mean difference (SMD) and the y-axis displaying the standard error (SE) of the SMD. The vertical dashed line indicates the overall effect size. The plots of quality of life, non-motor symptoms and psychiatric symptoms show a relatively symmetrical distribution of studies around the central effect estimate
