# Supplementary material for: The Effects of Digital Health Interventions on Motor Symptoms, Nonmotor Symptoms, and Quality of Life in Patients With Parkinson Disease: Systematic Review and Meta-Analysis of Randomized Controlled Trials
Source: J Med Internet Res. 2026 Mar 12;28:e79935. doi: 10.2196/79935 (PMC13147926; doi:10.2196/79935)
Supplement: Multimedia Appendix 11 [file jmir_v28i1e79935_app11.docx]

**Multimedia Appendix 10. Meta-regression analyses of the effect on motor symptoms, quality of life, cognitive function and psychiatric symptoms.**

| **Motor symptoms** | **^a^β (95% CI)** | **^b^QM** | **P value** | **^c^R^2^** |
| --- | --- | --- | --- | --- |
| mean age | -0.05 (-0.27, 0) | 4.22 | 0.04 | 2.96% |
| percentage of females | -1.08 (-2.5, 0.34) | 2.29 | 0.14 | 0.00% |
| disease severity | -0.14 (-0.67, 0.38) | 0.30 | 0.59 | 0.00% |
| publication years | 0 (-0.05, 0.05) | 0.00 | 0.98 | 0.00% |
| intervention duration | -0.01 (-0.03, 0.01) | 0.48 | 0.49 | 0.00% |
| Country income level | 0.25 (-0.88, 2.62) | 1.02 | 0.37 | 0.00% |
| intervention setting | 0.02 (-0.42, 0.45) | 0.01 | 0.94 | 0.00% |
| type of control | 0.26 (-0.43, 0.95) | 0.56 | 0.46 | 0.00% |
| intervention type | - | 0.42 | 0.74 | 0.00% |
| intervention purpose | - | 0.69 | 0.60 | 0.00% |
| supervision | - | 1.50 | 0.22 | 3.96% |
| **Psychiatric symptoms** | **β (95% CI)** | **QM** | **P value** | **R^2^** |
| mean age | 0 (-0.08, 0.07) | 0.01 | 0.92 | 0.00% |
| percentage of females | 1.38 (-0.33, 3.1) | 2.73 | 0.11 | 7.42% |
| disease severity | 0.64 (-1.34, 2.61) | 0.50 | 0.49 | 0.00% |
| publication years | -0.08 (-0.16, 0.01) | 3.39 | 0.08 | 4.93% |
| intervention duration | 0.01 (-0.02, 0.04) | 0.30 | 0.59 | 0.00% |
| Country income level | -0.29 (-1.25, 0.68) | 0.38 | 0.54 | 2.64% |
| intervention setting | 0.02 (-0.71, 0.75) | 0.00 | 0.95 | 0.00% |
| type of control | -0.18 (-1.01, 0.65) | 0.20 | 0.66 | 0.00% |
| intervention type | - | 0.23 | 0.87 | 0.00% |
| intervention purpose | - | 1.44 | 0.25 | 11.19% |
| supervision | - | 1.08 | 0.37 | 0.00% |
| **Cognitive function** | **β (95% CI)** | **QM** | **P value** | **R^2^** |
| mean age | 0.01 (-0.06, 0.09) | 0.08 | 0.78 | 0.49% |
| percentage of females | -0.2 (-1.43, 1.04) | 0.11 | 0.74 | 0.00% |
| disease severity | 0.18 (-0.99, 1.35) | 0.11 | 0.74 | 0.00% |
| publication years | 0.04 (-0.02, 0.11) | 1.96 | 0.17 | 0.00% |
| intervention duration | -0.01 (-0.03, 0.02) | 0.43 | 0.52 | 0.00% |
| Country income level | 0.13 (-0.61, 0.87) | 0.14 | 0.72 | 0.00% |
| intervention setting | 0.27 (-0.24, 0.77) | 1.16 | 0.29 | 8.88% |
| type of control | -0.04 (-0.61, 0.53) | 0.02 | 0.90 | 0.00% |
| intervention type | - | 0.55 | 0.58 | 0.00% |
| intervention purpose | - | 1.05 | 0.40 | 0.00% |
| supervision | - | 0.43 | 0.74 | 0.00% |
| **Quality of life** | **β (95% CI)** | **QM** | **P value** | **R^2^** |
| mean age | -0.03 (-0.1, 0.04) | 0.90 | 0.35 | 0.64% |
| percentage of females | 0.72 (-1.12, 3.56) | 0.63 | 0.43 | 0.00% |
| disease severity | 0.53 (-0.64, 0.75) | 0.02 | 0.88 | 0.00% |
| publication years | -0.03 (-0.1, 0.03) | 1.12 | 0.30 | 0.00% |
| intervention duration | 0 (-0.03, 0.03) | 0.01 | 0.94 | 0.00% |
| Country income level | 0.09 (-0.58, 0.76) | 0.08 | 0.78 | 0.00% |
| intervention setting | -0.07 (-0.7, 0.55) | 0.05 | 0.82 | 0.00% |
| type of control | 0.17 (-0.87, 1.22) | 0.11 | 0.74 | 0.00% |
| intervention type | - | 0.34 | 0.88 | 0.00% |
| intervention purpose | - | 0.16 | 0.85 | 0.00% |
| supervision | - | 1.41 | 0.25 | 10.38% |

^a^β (95% CI): standardized regression coefficient with 95% confidence interval (indicates direction and magnitude of association)

^b^QM: Cochran’s Q-test for model significance (assesses whether predictors explain heterogeneity)

^c^R²: proportion of between-study variance accounted for by predictors
