# Supplementary figures and images for: The Effects of Digital Health Interventions on Motor Symptoms, Nonmotor Symptoms, and Quality of Life in Patients With Parkinson Disease: Systematic Review and Meta-Analysis of Randomized Controlled Trials
Source: J Med Internet Res. 2026 Mar 12;28:e79935. doi: 10.2196/79935 (PMC13147926; doi:10.2196/79935)

**Multimedia Appendix 11. Bubble plots for the continuous variables in meta-regression.**


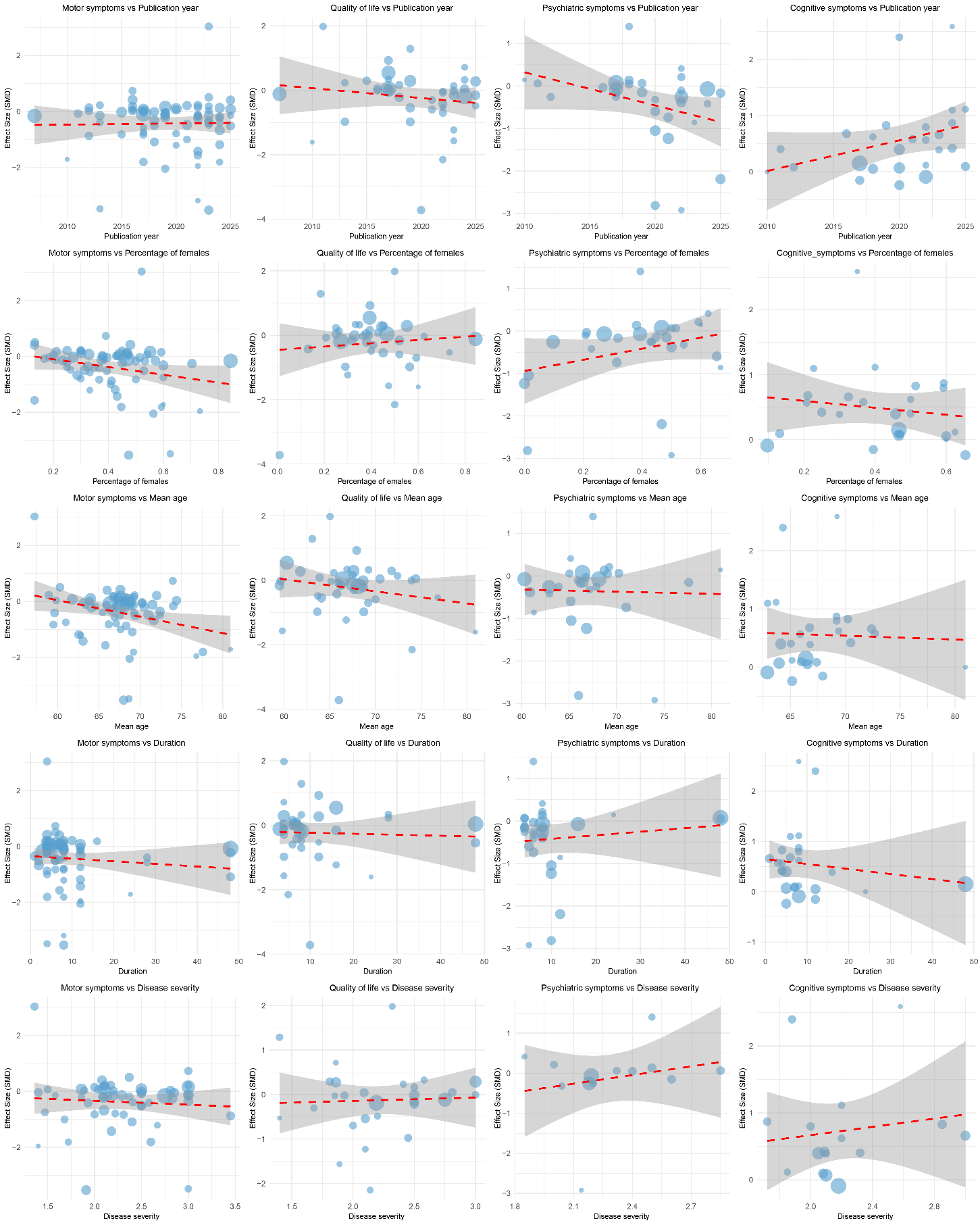

Supplement: Multimedia Appendix 12 [file jmir_v28i1e79935_app12.docx]
