# Supplementary material for: The Effects of Digital Health Interventions on Motor Symptoms, Nonmotor Symptoms, and Quality of Life in Patients With Parkinson Disease: Systematic Review and Meta-Analysis of Randomized Controlled Trials
Source: J Med Internet Res. 2026 Mar 12;28:e79935. doi: 10.2196/79935 (PMC13147926; doi:10.2196/79935)
Supplement: Multimedia Appendix 13 [file jmir_v28i1e79935_app13.docx]

**Multimedia Appendix 12. Reach, adoption, drop-out rate, and feasibility of included interventions.**

| **Study ID** | **Percentage reach (Randomly assigned proportion)** | **Adoption** | **Drop-out rate** | **Feasibility** |
| --- | --- | --- | --- | --- |
| Albert, 2023 | 50.9% (52.9%) | 100% | 11.1% | ^a^NR; reported high retention, adherence, usability, and satisfaction. |
| Allen, 2017 | 32.2% (50.0%) | 97% | 21.1% | feasible based on that participants were able to learn to use the system and play the games within two home visits, progressing to more difficult levels of gameplay, and enjoying playing the exergames. |
| Bartolo, 2024 | 38.8% (50%) | 100% | 7.7% | NR; reported that most subjects described the intervention as simple, motivating, and easily usable, possibly suitable for home use. |
| Beck, 2017 | 46.2% (49.7%) | 91% | 3.1% | feasible based on a high percentage of users who completed at least one virtual visit (98%), completed as scheduled (91%); reported high satisfaction (97%), and preferred virtual visits. |
| Bernini, 2019 | 29.9% (56.1%) | NR | 26.1% | NR; reported only effectiveness. |
| Bernini, 2021 | NR (39.6%) | NR | 14.3% | NR; reported only effectiveness. |
| Bogosian, 2022 | 33.0% (50%) | 26.67% | 26.7% | feasible based on high interest, high percentage of eligible participants who consented to take part, high adaption (73.3% attended 4 or more of the 8 sessions), high retention rates; and convenience. |
| Calabrò, 2019 | 37.3% (50%) | NR | 0.0% | NR; reported only effectiveness. |
| Capecci, 2019 | 37.3% (54.5%) | NR | 20.0% | NR; reported only effectiveness. |
| Carda, 2012 | 4.5% (50%) | NR | 6.7% | NR; the intervention was safe and well-accepted by patients. |
| Carpinella, 2016 | 40.7% (52.4%) | NR | 22.7% | feasible based on high effectiveness and acceptance; intervention was considered reliable, easy to use, safe, and comfortable. |
| Çetin, 2024 | 35.3% (52.2%) | NR | 16.7% | NR |
| Constantinescu, 2011 | 50% (50%) | 100% | 0.0% | NR; reported high satisfaction and non-inferiority. |
| Cubo, 2025 | 94.3%(50%) | NR | 16% | NR;reported no significant adverse events. |
| Da Silva, 2022 | 28.8% (46.7%) | 100% | 14.3% | NR; the intervention is feasible based on good adherence and safety with no serious adverse events. |
| Das, 2024 | 29.8% (50%) | 98% | 0.0% | feasible based on a good recruitment rate, high retention rate, high adherence, and no safety or usability issues. |
| De Luca, 2019 | NR (50%) | NR | 0.0% | NR; reported only effectiveness. |
| de Melo, 2018 | 24.1% (46.4%) | NR | 7.7% | NR; reported only effectiveness. |
| De, 2025 | 94.7%(50%) | NR | 2.80% | all patients indicated that the training dose was feasible and 88% of patients thought the training was fun and interesting. 56% perceived the training as suitable. |
| Del Pino, 2023 | NR (50%) | NR | 0.0% | NR; reported effectiveness, high adherence, cost-effectiveness, usefulness, and novelity. |
| Dhamija, 2025 | 48.8%(56.2%) | 71.4% of intervention group and 82.1% of control group completed at least 75% of the total sessions. | 1.60% | NR;reported no adverse event or fall. |
| Dobkin, 2020 | 46.8% (51.4%) | NR | 10.8% | NR; reported only effectiveness. |
| Dobkin, 2021 | 37.2% (50%) | 86.8% | 13.3% | NR; reported high feasibility, high satisfaction, and a high adherence rate. |
| Dorsey, 2010 | 42.8% (42.8%) | 100% | 0.0% | feasible based on high adoption (100%/92%), that 13 of all 14 participants opted to receive their PD care via telemedicine; and reported high satisfaction. |
| Dorsey, 2013 | 45% (45%) | 93% | 0.0% | feasible based on a high percentage of users who completed 27 scheduled telemedicine visits (93%), with no harm or unintended effects. None of the individuals randomized to telemedicine required an in-person visit; and reported increased flexibility, convenience, and effectiveness. |
| Edwards, 2013 | 47.3% (50.6%) | 69% | 27.3% | NR; feasible based effectiveness without requiring extensive personnel time or costs. |
| Eldemir, 2023 | 9.5% (50%) | NR | 6.2% | NR; reported only effectiveness. |
| Ellis, 2019 | 16.8% (51.0%) | NR | 11.5% | NR; reported favorable satisfaction ratings; interventions were well tolerated and acceptable with no serious adverse intervention-related events; 82% of participants wanted to continue, and 100% would have recommended it to others. |
| Fellman, 2020 | 40.6% (48.1%) | NR | 0.0% | feasible based on high adherence, cost-effectiveness, and effectiveness. |
| Feng, 2019 | 45.2% (50%) | NR | 0.0% | NR; reported only effectiveness. |
| Ferraz, 2018 | 28.9% (30.6%) | NR | 9.1% | NR; reported only effectiveness. |
| Flynn, 2021 | 17.2% (50%) | 84% | 5.0% | feasible based on high adherence and no adverse event; all the participants reported that the intervention was helpful and would recommend it to others. |
| Furnari, 2017 | 35.2% (50%) | NR | 0.0% | NR; reported only effectiveness. |
| Galli, 2016 | 14.2% (50%) | 100% | 0.0% | NR; reported only effectiveness. |
| Gandolfi, 2017 | 28.1% (50%) | NR | 5.3% | NR; is a feasible alternative to reduce postural instability, and has a relatively low cost. |
| Giehl, 2020 | 27.0% (47.9%) | NR | 17.4% | NR |
| Giehl, 2020 | 43.5% (48.7%) | NR | 13.5% | NR; reported only positive effects. |
| Ginis, 2016 | 41.5% (55%) | NR | 9.1% | feasible based on high adherence and user-friendliness; participants were very positive about the intervention, and some of them were very enthusiastic about it. |
| Glicia Pedreira, 2013 | 31.0% (50%) | NR | 27.3% | NR; reported effectiveness, high participant interactivity, and satisfaction. |
| Goffredo, 2017 | 34.6% (51.4%) | NR | 9.3% | NR; feasible based on low drop-out rate; reported effectiveness. |
| Gryfe, 2022 | 27.6% (31.7%) | 97.60% | 0.0% | NR; reported high adherence, high safety with fewer adverse events, and effectiveness. |
| Gulcan, 2023 | 35.4% (50%) | NR | 11.8% | NR; reported only effectiveness. |
| Hajebrahimi, 2022 | 37.5% (50%) | NR | 26.7% | NR; reported only beneficial effects. |
| Halpern, 2012 | 40% (50%) | NR | 0.0% | feasible based on effectiveness, and that participants were able to independently use the Companion at home, and rated the device as very helpful. |
| Han, 2023 | NR (50%) | NR | 6.7% | NR; reported only effectiveness. |
| Harpham, 2025 | 81.2%(53.8%) | 78.40% | 7.70% | feasible based on mild adverse events and high engagement. |
| Hashemi, 2022 | 53.6% (66.7%) | NR | 0.0% | NR; reported only effectiveness. |
| Heldman, 2017 | 42.8% (50%) | 95.7% | 0.0% | NR; reported high compliance and usability. |
| Isaacson, 2019 | 35.1% (50%) | NR | 10.5% | NR; reported only effectiveness. |
| Jäggi, 2023 | 36.8% (50%) | 96.50% | 9.5% | feasible based on usability, high adherence, low attrition rate, and high safety. |
| Johnson, 2024 | 44.4% (60%) | NR | 0.0% | feasible based on recruitment success, high satisfaction, high safety, and effectiveness. |
| Jong-Hoon, 2020 | 50% (50%) | NR | 0.0% | NR; reported only effectiveness. |
| Kashif, 2022 | 44% (50%) | NR | 9.1% | NR; reported only effectiveness. |
| Kashif, 2024 | 30.3% (33.3%) | NR | 0.0% | NR |
| Kawashima, 2022 | 41.2% (46.7%) | NR | 28.6% | NR; reported no serious adverse events and considerable acceptability. |
| Kegelmeyer, 2024 | 21.5% (51.1%) | NR | 13.0% | NR; reported safety. |
| Khalil, 2017 | 14.8% (53.3%) | 77% | 6.2% | feasible based on high recruitment, retention, and compliance rates and low adverse events. |
| Kim, 2022 | 23.2% (50%) | NR | 9.1% | NR; reported only effectiveness. |
| Kluger, 2023 | NR (49.9%) | NR | NR | NR; intervention is feasible and may improve QOL and advance care planning; no adverse events. |
| Kraepelien, 2020 | 14.6% (49.4%) | 89% | 10.5% | feasible based on high satisfaction, high adherence (89% completed at least the first four modules), and high self-rated benefits. |
| Kratz, 2025 | 39.2%(10%) | NR | 5.00% | feasible based on that all teletherapy sessions proceeded without technical complications and with adequate audio quality for intervention delivery, and participants rated the usability as good to excellent. |
| Lai, 2020 | 33.3% (50%) | 99.2% | 0.0% | NR; reported high adherence. |
| Lakshminarayana, 2017 | 14.4% (49.3%) | 72% | 35.8% | NR; reported higher medication adherence and higher quality of clinical consultation. |
| Lau, 2022 | 40.9% (50%) | 94.4% | 0.0% | feasible based on high adherence (94.4%), no drop-out and no adverse events. |
| Lee, 2025 | 76.7%(69.6%） | 79.36% | NR | feasible based on high adherence. |
| Li, 2022 | 21.4% (33.3%) | NR | 14.8% | NR; reported only effectiveness. |
| Liao, 2015 | 27.9% (33.3%) | NR | 0.0% | NR; reported only effectiveness. |
| Maas, 2024 | 26.1% (50%) | NR | 9.2% | NR; reported only effectiveness. |
| Maggio, 2018 | NR (50%) | NR | 0.0% | NR; reported only effectiveness. |
| Maggio, 2024 | NR (70.6%) | NR | NR | NR; reported only effectiveness. |
| Maggio, 2025 | NR(50%) | NR | 0.0% | feasible based on no adverse events and no drop-out. |
| Manor, 2013 | NR (50%) | NR | 0.0% | NR; reported effectiveness and high satisfaction (confidence in the intervention, adequate provision of explanation on the treatment, spending enough time, and putting the patient's needs first). |
| Maranesi, 2022 | 50% (50%) | NR | 0.0% | NR; reported only effectiveness. |
| McGibbon, 2024 | NR (48.1%) | NR | NR | NR; reported only effectiveness. |
| Meng-Che, 2016 | 22.9% (50%) | NR | 9.1% | NR; reported only effectiveness. |
| Mirelman, 2016 | 23.3% (51.0%) | 92.30% | 18.2% | NR; reported effectiveness, safety (few adverse events), high retention rate (81%), fidelity of the approach used, feasibility, and broad applicability. |
| Nieuwboer, 2007 | 26.3% (49.7%) | NR | 0.0% | NR; reported only effectiveness. |
| Nuvolini, 2025 | 73.4%(42.5%) | NR | 19.10% | NR;reported only effectiveness. |
| Ophey, 2020 | 43.5% (48.7%) | 97.1% | 5.4% | feasible based on high adherence(all participants completed the training, and they attended 24.27 ± 1.59 out of max. 25 training sessions), high levels of motivation, and satisfaction. |
| Özden, 2021 | NR (49.0%) | NR | 0.0% | NR; reported only effectiveness. |
| París, 2011 | 39.1% (54.5%) | NR | 11.1% | NR; reported only effectiveness. |
| Pastana Ramos, 2023 | 30.8% (42.1%) | 100% | 12.5% | feasible based on high adherence (all participants completed all the training) and high safety(no adverse event). |
| Patel, 2017 | 48.3% (50%) | NR | 42.9% | NR; reported effectiveness and a high drop-out rate. |
| Peacock, 2021 | NR (53.3%) | NR | 18.8% | NR |
| Picelli, 2012 | 26.2% (50%) | NR | 5.9% | NR; reported only effectiveness. |
| Picelli, 2013 | 20.6% (33.3%) | NR | 0.0% | NR; reported no adverse events and effectiveness. |
| Picelli, 2015 | 31.4% (50%) | NR | 0.0% | NR; reported no drop-out and no adverse events. |
| Piers, 2023 | 42.1% (57.1%) | NR | 25.0% | feasible based on high adherence, high treatment fidelity, and positive feedback. |
| Pinto, 2025 | 85.1%(66.7%) | 87.90% | 22.80% | feasible based on high participant adherence, with over 80% logging into the app and completing most sessions, and high acceptability, with 80% of users rating the app as acceptable and 76% agreed that the PACT app improved their well-being. |
| Pompeu, 2012 | 32% (50%) | NR | 0.0% | NR; reported effectiveness, no drop-out, and no adverse events. |
| Qayyum, 2022 | NR (50%) | NR | NR | NR; reported only effectiveness. |
| Raciti, 2022 | 45.4% (50%) | NR | 0.0% | NR; reported only effectiveness. |
| Raglio, 2023 | NR (52.6%) | NR | NR | NR; reported no adverse effects. |
| Ribas, 2017 | 33.3% (50%) | NR | 0.0% | NR; reported effectiveness, no drop-out, and no adverse events. |
| Sale, 2013 | 14.7% (50%) | 100% | 0.0% | feasible based on high adherence (100%) and high acceptability. |
| Santos, 2019 | 45.4% (66.6%) | NR | 6.7% | NR; reported only effectiveness. |
| Sekimoto, 2019 | NR (100%) | NR | 0.0% | feasible based on high satisfaction and high safety (no adverse events). |
| So, 2023 | 27.2% (50%) | 80%–100% | 32.0% | feasible based on high satisfaction and high attendance; participants expressed that the intervention was helpful and beneficial. |
| Song, 2018 | 24.6% (51.7%) | 86% | 19.4% | NR |
| Spina, 2021 | 19.0% (50.0%) | NR | 0.0% | NR; reported only effectiveness. |
| Svaerke, 2022 | NR (66.7%) | 62.5% | 20.0% | feasible based on effectiveness; reported few adverse events. |
| Tagliente, 2025 | 78.9%(50%) | 100% | 16.70% | NR;reported only effectiveness. |
| Tayyebi, 2025 | 68.2%(50%) | NR | 0.0% | NR;reported only effectiveness. |
| Theodoros, 2015 | 30.5% (69.2%) | NR | 0.0% | NR; reported only noninferiority and validity. |
| van Balkom, 2022 | 26.7% (50%) | NR (Compliance was excellent with the majority of participants completing 100% of the intervention) | 4.4% | NR; reported high compliance. |
| van de Weijer, 2020 | NR (51.2%) | 68.2% | 14.3% | feasible based on high adherence (the average training completion was 68.2% and high satisfaction, all participants understood the goal of the game, and 75% of participants would recommend this game to others, but the technical issues may hindered adherence rates and therapy compliance. |
| van den Heuvel, 2014 | 28.8% (51.5%) | NR (median (interquartile range) number of attended sessions was (8.0–10.0)) | 0.0% | feasible based on high adherence and no adverse events, the intervention was well received, suitable for use, and could be operated independently. |
| Wilkinson, 2016 | 62% (50%) | NR (average number of visits was 2.7) | 61.1% | NR; reported high satisfaction, effectiveness, and improved healthcare utilization. |
| Yang, 2016 | 40.7% (47.8%) | NR | 9.1% | NR; feasible based effectiveness. |
| Yen, 2011 | 20.9% (33.3%) | NR | 14.3% | NR; reported only effectiveness. |
| Yuan, 2020 | NR (50.0%) | NR | NR | NR; reported no adverse events and effectiveness. |
| Zoetewei, 2024 | 42.7% (50.8%) | NR | 19.4% | NR; reported only effectiveness. |

^a^NR: Not Reported
